# Protective Signature of IFNγ-Stimulated Microglia Relies on miR-124-3p Regulation From the Secretome Released by Mutant APP Swedish Neuronal Cells

**DOI:** 10.3389/fphar.2022.833066

**Published:** 2022-05-10

**Authors:** Gonçalo Garcia, Adelaide Fernandes, Frank Stein, Dora Brites

**Affiliations:** ^1^ Neuroinflammation, Signaling and Neuroregeneration Laboratory, Research Institute for Medicines (iMed.ULisboa), Faculty of Pharmacy, Universidade de Lisboa, Lisbon, Portugal; ^2^ Department of Pharmaceutical Sciences and Medicines, Faculty of Pharmacy, Universidade de Lisboa, Lisbon, Portugal; ^3^ Central Nervous System, Blood and Peripheral Inflammation, Research Institute for Medicines (iMed.ULisboa), Faculty of Pharmacy, Universidade de Lisboa, Lisbon, Portugal; ^4^ Proteomics Core Facility, European Molecular Biology Laboratory (EMBL), Heidelberg, Germany

**Keywords:** neuronal miR-124-3p mimic/inhibitor, IFNγ-primed CHME3 microglia, SH-SY5Y APPSwedish cell, inflammatory gene expression, mir-124-dependent microglia proteomic changes, MMP-2/MMP-9 deactivation, secretome/exosome (sEVs) paracrine signaling, miRNA-depleted microglia with siDicer1

## Abstract

Microglia-associated inflammation and miRNA dysregulation are key players in Alzheimer’s disease (AD) pathophysiology. Previously, we showed miR-124 upregulation in APP Swedish SH-SY5Y (SWE) and PSEN1 iPSC-derived neurons and its propagation by the secretome (soluble and exosomal fractions). After modulation with miR-124 mimic/inhibitor, we identified common responsive mechanisms between such models. We also reported miR-124 colocalization with microglia in AD patient hippocampi. Herein, we determined how miR-124 modulation in SWE cells influences microglia polarized subtypes in the context of inflammation. We used a coculture system without cell-to-cell contact formed by miR-124 modulated SWE cells and human CHME3 microglia stimulated with interferon-gamma (IFNγ-MG), in which we assessed their adopted gene/miRNA profile and proteomic signature. The increase of miR-124 in SWE cells/secretome (soluble and exosomal) was mimicked in IFNγ-MG. Treatment of SWE cells with the miR-124 inhibitor led to RAGE overexpression and loss of neuronal viability, while the mimic caused RAGE/HMGB1 downregulation and prevented mitochondria membrane potential loss. When accessing the paracrine effects on microglia, SWE miR-124 inhibitor favored their IFNγ-induced inflammatory signature (upregulated RAGE/HMGB1/iNOS/IL-1β; downregulated IL-10/ARG-1), while the mimic reduced microglia activation (downregulated TNF-α/iNOS) and deactivated extracellular MMP-2/MMP-9 levels. Microglia proteomics identified 113 responsive proteins to SWE miR-124 levels, including a subgroup of 17 proteins involved in immune function/inflammation and/or miR-124 targets. A total of 72 proteins were downregulated (e.g., MAP2K6) and 21 upregulated (e.g., PAWR) by the mimic, while the inhibitor also upregulated 21 proteins and downregulated 17 (e.g., TGFB1, PAWR, and EFEMP1). Other targets were associated with neurodevelopmental mechanisms, synaptic function, and vesicular trafficking. To examine the source of miR-124 variations in microglia, we silenced the RNase III endonuclease Dicer1 to block miRNA canonical biogenesis. Despite this suppression, the coculture with SWE cells/exosomes still raised microglial miR-124 levels, evidencing miR-124 transfer from neurons to microglia. This study is pioneer in elucidating that neuronal miR-124 reshapes microglia plasticity and in revealing the relevance of neuronal survival in mechanisms underlying inflammation in AD-associated neurodegeneration. These novel insights pave the way for the application of miRNA-based neuropharmacological strategies in AD whenever miRNA dysregulated levels are identified during patient stratification.

## 1 Introduction

Alzheimer’s disease (AD) is the most prevalent neurodegenerative disorder in developed countries. The most common molecular hallmarks are extracellular β-amyloid (Aβ) plaques and neurofibrillary tangles of hyperphosphorylated tau protein. Besides, alterations in the neuroinflammatory status caused by dysregulated microglia are also reported in AD (Leng and Edison, 2021). Initially assumed to be excessively activated in response to amyloid deposition and neuronal dysfunction, evidence indicates that microglia actively participate in AD pathogenesis since disease early stages (Fan et al., 2017). The identification of new potential therapeutic microglial targets (Sierksma et al., 2020), as well as the classification of several microglial subtypes in health and disease (Uriarte Huarte et al., 2021), created new opportunities for immunomodulation-based strategies in opposition to previous approaches based on counteracting or inducing a unique phenotype (Deczkowska et al., 2018). However, the lack of translation from the bench to the clinic is still one of the most limiting factors for the success of drug development in AD (Vitek et al., 2020). Many studies rely on specific animal models, such as rodents, which do not entirely share the human disease pathophysiology. Despite not fully recapitulating the human disease and/or brain inflammation in a dish, human cell models are considered important steps toward AD mechanistic studies (Drummond and Wisniewski, 2017). In addition, though still controversial, another key point is to define which approach should be used in AD pathogenicity to reprogram microglia into protective and pro-regenerative phenotypes and in which context, disease stage, and patient subtypes should be applied.

miRNAs have gained increasing interest in the field of AD, emerging as potential biomarkers in several diseases, as their expression is altered in different patients, stages, and models (Fernandes et al., 2018; Kumar and Reddy, 2018; Brites, 2020; Garcia et al., 2021). By regulating hundreds of several targets in different cells and tissues, miRNA modulation may be a key strategy to counteract multifactorial and complex disorders, including neurodegenerative and cancer diseases (Saito and Saito, 2012). Specific miRNAs are known to regulate essential functions that are compromised in AD, such as neurite outgrowth and synaptic plasticity (Hu and Li, 2017; McGowan et al., 2018; Garcia et al., 2021), revealing their potential also as therapeutic targets. In particular, miR-124, as one of the most predominant cerebral miRNAs, was shown to be critically involved in multiple neuronal mechanisms, such as differentiation, axonal growth, synaptic function, and homeostasis maintenance (Sun et al., 2015; Xue et al., 2016; Yardeni et al., 2018), and to be dysregulated upon stress conditions (Sun et al., 2015). Though miR-124 is almost exclusively expressed by neurons (Åkerblom et al., 2012), we found miR-124 colocalization with microglia in hippocampal slices from Braak stage IV AD patients, together with a general miR-124 overexpression in whole hippocampal lysates that revealed significantly upregulated levels in Braak stage III patient postmortem samples (Brites, 2020). In contrast, accumulating studies demonstrate consistent benefits of miR-124 in microglia by triggering their pro-regenerative function (Ponomarev et al., 2011; Yu et al., 2017; Fumagalli et al., 2018). Together, these reports confirmed that miR-124 is closely involved in AD pathological processes and may play an important regulator role in microglial function. However, it is still unclarified if microglia are stimulated to overexpress miR-124 in a specific context or suffer the influence of miR-124 in the cell microenvironment.

The role of miR-124 in AD is still a matter of debate, with contradicting reports demonstrating increased (Wang et al., 2018) and decreased (An et al., 2017) levels in the disease and different perspectives about its *modus operandi*. It seems clear that miR-124 is a key player in AD (Sonntag et al., 2012), though its function may critically depend on the specific requirements of cell metabolism and the model used to recapitulate the condition (Garcia et al., 2021). Despite being able to control different AD hallmarks in SH-SY5Y cells constitutively expressing the APP Swedish form (SWE) and in neurons differentiated from induced pluripotent stem cells (iPSCs) generated from a patient with the PSEN1ΔE9 mutation, in such study, we showed that miR-124 was upregulated in both cell models and their derived exosomes (EXOs). Such findings support the potential of the SWE cell model to further explore the miR-124 dynamics in neuron-microglia cultures and investigate how it may contribute to microglia polarization/depolarization under an inflammatory condition. For instance, miR-124-loaded EXOs were revealed to attenuate microglia activation by cocaine (Chivero et al., 2020), supporting miRNA-based strategies as new delivery systems for therapeutic intervention in AD. Such small extracellular vesicles, herein identified as EXOs, are recognized as Aβ seeders and propagators of AD pathological mediators between brain regions (Eitan et al., 2016; Yuyama and Igarashi, 2017). Moreover, EXOs are believed to act as paracrine vectors in miRNA specialized delivery to recover target cell function (Shirazi et al., 2021; Zheng et al., 2021). In that regard, we have previously shown that neuronal-derived EXOs carrying miR-124 modify microglia function and their immune properties (Pinto et al., 2017; Fernandes et al., 2018). This finding is not without precedent, since multiple therapeutic approaches using EXO-based delivery of miR-124 have been recently developed (Lee et al., 2017; Yang et al., 2017; Jiang et al., 2020).

In this study, we first validated increased miR-124 levels in the SWE cells when compared to SH-SY5Y (SH) counterparts. Then, we investigated how the miR-124 release by AD neuronal cells influenced microglia activation when both cells were in coculture. For that, we stimulated the human CHME-3 microglia with interferon-gamma (IFNγ), a well-known pro-inflammatory cytokine, reported to induce microglia activation in the AD context (Abbas et al., 2002; Mastrangelo et al., 2009; Belkhelfa et al., 2014). After establishing and characterizing the SWE-CHME3 cell coculture, we transfected SWE cells with the miR-124 inhibitor or the mimic to reduce or increase its expression levels, respectively. We monitored the consequences of downregulating and upregulating miR-124 in each of the cocultured cells and their secretome, with emphasis on inflammatory gene expression signature and the microglial proteomic profile. Finally, we assessed the neuron-microglia trafficking of miR-124, either in the coculture experiments or when incubating IFNγ-stimulated CHME-3 microglia with SWE-derived exosomes. Our results provide novel insights on neuronal miR-124 as a powerful neuro-immune regulator of microglia gene expression signature and highlight its modulation as representing a promising double-edge sword strategy in the AD field targeting both neurons and microglia.

## 2 Materials and Methods

### 2.1 Culture and Differentiation of Human SH-SY5Y Neuronal Cell Lines

SH and SWE cells were a gift from Professor Anthony Turner (Belyaev et al., 2010). Cells were maintained in Dulbecco’s Modified Eagle’s Medium (DMEM) (Gibco, Thermo Fisher Scientific, Waltham, MA, United States), supplemented with 10% fetal bovine serum (FBS) and 2% AB/AM in T75 flasks under a humidified atmosphere with 5% CO_2_, at 37°C, as we previously described (Fernandes et al., 2018; Garcia et al., 2021). For experiments, cells were seeded onto 12-well plates coated with poly-d-lysine (100 μg/ml, Sigma-Aldrich, St. Louis, MO, United States) and laminin (4 μg/ml, Gibco) at a final concentration of 5 × 10^4^ cells per well, and differentiated with retinoic acid (RA) at the concentration of 10 μM, administered every day in fresh medium, until day 7 (Korecka et al., 2013; Garcia et al., 2021). Only for immunocytochemistry, cells were plated onto 12-well plates containing HCl-washed coverslips with the same poly-d-lysine/laminin coating described above.

### 2.2 Culture and Stimulation of Human CHME3 Microglia Cell Line

Human CHME3 microglial cells, also known as HMC3, were kindly provided by Professor Marc Tardieu (Janabi et al., 1995). Cells were cultured in T75 culture flasks in DMEM supplemented with 10% FBS, 2% AB/AM (Sigma-Aldrich) and 1% l-glutamine (L-glu) (Sigma-Aldrich) in a humidified atmosphere containing 5% CO_2_, at 37°C, as usual in our lab (Fernandes et al., 2018). Medium was changed every other day. For experiments, cells were seeded onto 12-well non-coated plates, at a final concentration of 5 × 10^4^ cells per well. To mimic microglia phenotypes in an inflammatory milieu we stimulated the cells with human IFNγ (BACHEM, Bubendorf, Switzerland) at 50 ng/ml during 2, 12, and 24 h.

### 2.3 Evaluation of Cell Viability by the Nexin Assay

In order to determine the viability of either neuroblastoma or microglial cells, both floating and adherent cells detached with trypsin were collected, mixed, and spun down at 500 g for 5 min. Then, pellets were resuspended in 1% bovine serum albumin (BSA) in PBS and stained with phycoerythrin-conjugated annexin V (V-PE) and 7-amino-actinomycin D (7-AAD), using the Guava Nexin Reagent^®^ (Merck Millipore, Burlington, MA, United States). Stained cells were analyzed using a flow cytometer (Guava easyCyte 5 HT, Merck-Millipore), operated by Guava Nexin Software. Four cellular populations were distinguished: viable cells (annexin V-PE and 7-AAD double-negative), early apoptotic cells (annexin V-PE positive and 7-AAD negative), late apoptotic cells (annexin V-PE and 7-AAD double-positive), and necrotic cells/cellular debris (annexin V-PE negative and 7-AAD positive).

### 2.4 Isolation and Characterization of EXOs

Cells were cultured for 24 h in FBS-free medium to ensure sufficient EXO release and prevent the influence of FBS-associated EXOs. Cell culture media were collected from at least three independent experiments, and EXOs were isolated using the differential ultracentrifugation, as we previously described (Fernandes et al., 2018; Garcia et al., 2021). Briefly, equal volumes of cell media were promptly centrifuged at 1,000 g for 10 min to pellet cell debris. The supernatants were transferred into new tubes and centrifuged at 16,000 g for 1 h to pellet and discard large extracellular vesicles. The remaining supernatant containing EXOs was filtered through a 0.22 μm pore size membrane and centrifuged at 100,000 g for 2 h in an Ultra L-XP100 centrifuge (Beckman Coulter, Brea, CA, United States). The pellet was resuspended/washed in PBS and centrifuged once again at 100,000 g for 2 h. The pellet of EXOs was suspended in a 200 μl lysis buffer for RNA extraction with the miRCURY Isolation Kit-Cell to determine miRNA content (Exiqon, Gill StreetWoburn, MA, United States). For the characterization of EXO markers by western blot, EXOs isolated from three independent experiments of 40 ml supernatants were pooled, suspended in 50 µl cell lysis buffer (Cell Signaling, Danvers, MA, United States), transferred into microtubes, snap-frozen, and stored at −80°C until analysis. Three characteristic markers of EXOs (ALIX, CD63, and flotillin) were assessed by western blot. For transmission electron microscopy (TEM), freshly isolated EXOs were suspended and kept in ice-cold PBS during 1–2 days until analysis. Then, equal volumes of freshly isolated EXO suspensions were dried onto freshly “glow discharged” 300 mesh formvar/carbon-coated TEM grids (Ted Pella, Redding, CA, United States), negatively stained with 2% aqueous uranyl acetate and observed under a JEOL JEM 1400 transmission electron microscope (JEOL Ltd., Tokyo, Japan) at an accelerating voltage of 120 kV. Images were digitally recorded using a Gatan SC 1100 ORIUS CCD camera (Gatan Inc., Warrendale, PA, United States). Round cup-shaped structures, ranging from 50 to 200 nm size, were considered as EXOs.

### 2.5 Determination of Soluble miRNAs and Cytokines/Chemokines

EXO-free cell media (depleted in EXOs after differential ultracentrifugation) were used to evaluate soluble miRNAs and cytokine/chemokine content. For miRNA determination, total RNA was extracted from the media using the miRNeasy Serum/Plasma kit (Qiagen, Venlo, Netherlands) according to the manufacturer’s instructions and processed for RT-qPCR as detailed below. Concerning cytokine release, multiple cytokines and chemokines, including IL-1β, IL-8, IL-10, IL-18, TNF-α, and IL-6, were evaluated using the LEGENDplex multiplex immunoassay (BioLegend, San Diego, CA, United States), according to the manufacturer’s instructions. Data were recorded on a Guava easyCyte 5 HT flow cytometer (Merck Millipore), operated by Guava Nexin Software, and further processed by LEGENDplex™ Data Analysis Software V8.0 (BioLegend, San Diego, California).

### 2.6 RNA Extraction and RT-qPCR

Total RNA was extracted from neuroblastoma and CHME3 cells using TRIzol^®^ reagent (Life Technologies, Carlsbad, CA, United States), according to the manufacturer’s instructions. Total RNA obtained from the cells, EXOs, and EXO-free cell media was quantified in Nanodrop^®^ ND-100 Spectrophotometer (NanoDrop Technologies, Wilmington, DE, United States). In order to determine miRNA expression, equal amounts of RNA were reverse-transcribed into cDNA using the Universal cDNA Synthesis Kit (Qiagen). Then, miRNA expression was determined by RT-qPCR using the miRCURY LNA™ Universal RT miRNA PCR kit (Qiagen) with predesigned primers (Supplementary Table S1). Running conditions consisted of polymerase activation/denaturation and well-factor determination at 95°C for 10 min, followed by 50 amplification cycles at 95°C for 10 s and 60°C for 1 min (ramp-rate 1.6°C/s). In order to determine mRNA levels (gene expression), equal amounts of total RNA were reverse-transcribed into cDNA using the GRS cDNA Synthesis Master Mix kit (GRiSP, Porto, Portugal), and RT-qPCR was performed using Xpert Fast Sybr Blue (GRiSP) as a master mix with specific predesigned primers (Supplementary Table S1). Running conditions were as follows: 50°C for 2 min followed by 95°C for 2 min and finally 40 cycles at 95°C for 5 s and 62°C for 30 s. Both miRNA and mRNA RT-qPCRs were performed on a QuantStudio 7 Flex Real-Time PCR System (Applied Biosystems, Waltham, MA, United States). A melt-curve analysis was performed to verify amplification specificity immediately after the amplification protocol. Non-specific PCR products were not found. miRNA/gene expression data of at least four independewas inconsistentnt experiments were processed using the ΔΔCT method. Glyceraldehyde 3-phosphate dehydrogenase (GAPDH) was used as a reference gene for mRNAs because the β-actin expression was inconsistent upon miRNA modulation. For miRNA expression, U6 and spike-in were used as a reference miRNA and an internal standard, respectively. Results were normalized and expressed as 2^−ΔΔCT^ (fold-change) and/or log_2_-transformed, as appropriate. All samples were quantified in duplicate and compared to respective controls, depending on the performed analysis.

### 2.7 Quantification of Nitrite Levels

Nitric oxide (NO) levels in CHME3 microglia culture supernatants were estimated by determining the concentration of nitrites (NO_2_), the stable end-product generated from NO metabolism, using the Griess method, as previously published (Silva et al., 2011). Briefly, cell media was centrifuged at 15,000 *g* for 10 min to pellet cellular debris and mixed with the Griess reagent (1:1) in the 96-well tissue culture plates for 10 min, in the dark and at RT. A calibration curve was used for each assay. The absorbance at 540 nm was measured in duplicate samples, using a microplate reader, and the mean value used in the quantification.

### 2.8 Immunofluorescence, Image Acquisition, and Analysis

Cells were plated onto coverslips and cultured for the established periods of time. Then, cells were fixed with paraformaldehyde (4% w/v in PBS) for 20 min, washed with PBS, and permeabilized with Triton-X100 0.2% in PBS for 10 min. Blocking was performed with BSA at 3% in PBS for 30 min. F-actin was stained using AlexaFluor® 594 Phalloidin (1:100 in BSA 1% diluted in PBS, Thermo Fisher). As primary antibodies, mouse anti-iNOS (1:150) and/or mouse anti-MAP-2 (1:100) were used in separate experiments. As a secondary antibody, we used goat anti-mouse Alexa Fluor 488 (1:1,000). All antibodies were diluted in PBS containing 1% BSA. Then, coverslips were dipped in PBS for washing and incubated for 2 min with Hoechst 33258 dye diluted at 1:1,000 (BSA 1% in PBS) to stain nuclei. Excessive dye was removed by another PBS wash before coverslips were immersed in methanol and mounted on a glass slide with DPX mounting media. Fluorescence was recorded using an AxioScope A1 fluorescent microscope with an adapted camera AxioCam HRm (Zeiss, Oberkochen, Germany). Fluorescence images of at least ten representative random microscopic fields were acquired (original magnification: ×400). A iNOS fluorescence intensity per cell, from a total of 600 cells, (iNOS) was assessed with Fiji software tools (Schindelin et al., 2012). Dendrite length of individual neurons was measured from the immunofluorescence images using MAP-2 dendritic marker and the NeuronJ plugin included in Fiji software, as indicated before (Garcia et al., 2021).

### 2.9 MitoTracker Active Mitochondria Labeling

In order to stain active mitochondria, cells were incubated for 30 min at 37°C with 500 nM of MitoTracker Red CMXRos, according to the manufacturer’s instructions (Thermo Fisher Scientific). Then, they were fixed with 4% (w/v) paraformaldehyde, as described before (Garcia et al., 2021). Nuclei were stained with Hoechst 33,258 dye. Images were acquired, and total fluorescence intensity (FI) of the MitoTracker Red was assessed using Fiji software tools. Briefly, FI and cell area were automatically measured using (Analyze > Analyze Particles) with the options “area” and “integrated intensity” selected from the menu “set measurements.” Then, the FI of MitoTracker Red intensity of more than 300 cells of each condition was normalized to the respective cell area.

### 2.10 Modulation of miR-124 Levels in Neuroblastoma SWE Cells

After 7 days of RA differentiation, SWE cells were changed to Optimem media (Gibco, Thermo Fisher) and transfected pre-miR-124-3p (mimic) and anti-miR-124-3p (inhibitor) (Ambion, Austin, TX, United States), each at 15 nM/well, in the presence of the transfection agent X-tremeGENE (100 µM) (Sigma-Aldrich), and cultured overnight. Mock transfected and negative controls (Scramble sequences provided by Ambion) for both mimic and inhibitor were equally performed in parallel under the same conditions. Transfection efficiency was ensured in each experiment after and before coculture. Because we observed that mock and negative controls produced identical results, only mock control was used as a reference to compare the effects of the mimic and the inhibitor in the next assays. For the isolation of EXOs, SWE cells were cultured in EXO-free FBS media for 24 h after transfection, before collection and secretome processing.

### 2.11 Neuroblastoma-Microglia Coculture

Both cells were initially cultured in separated 12-well plates at a final concentration of 5 × 10^4^ cells per well to establish a coculture of SWE neurons with CHME3 microglia, followed by differentiation and miR-124 modulation with mimic and inhibitor (only in SWE cells), or IFNγ stimulation (only in CHME3 microglia). In the case of CHME3 microglia, cells were plated in wells containing HCl-washed coverslips with four small paraffin spacers on the outlined border and transferred into the 12-well plates containing the SWE cells to establish cocultures, as previously described by us (Fernandes et al., 2018). Because the CHME3 and SWE cells were separated by the paraffin spacers, this type of coculture setup avoids cell physical contact, but it still allows intercellular communication through the cell media. Similar approaches have been used in other studies for isolating different cell types from the same coculture showing no signs of cross-contamination (Phatnani et al., 2013; Wasilewski et al., 2022). Then, both cell types were cocultured during 2, 12, and 24 h in DMEM (Gibco, Thermo Fisher) with 1% FBS, 1% l-glutamine, and 2% AB/AM. Both CHME3-containing coverslips and SWE cells were separately processed for RNA/protein extraction immediately after each coculture period and selected time. The secretome (common to both cell types in the coculture) was immediately used for NO and matrix metalloproteinases (MMPs) determination, and the remaining media were frozen for further analysis.

### 2.12 Proteomics

#### 2.12.1 Mass Spectrometric (MS) Analysis

Cellular lysates of three experimental conditions were collected in triplicate and analyzed for proteomic analysis: 1) CHME-3 microglia cocultured with the non-modulated (mock control) SWE cells; 2) CHME-3 microglia cocultured with the SWE cells treated with the miR-124 inhibitor; and 3) CHME-3 microglia cocultured with the SWE cells treated with the miR-124 mimic. The cellular lysates were collected in TRIzol^®^ reagent (Life Technologies), and the protein fraction (denser) was isolated separately and immediately stored at −80°C. Total protein precipitation was performed by adding 10% trichloroacetic acid (TCA) to acetone, followed by three washing cycles with acetone containing 20 mM dithiothreitol (DTT) and centrifuged at 15,000 g for 10 min. The protein pellet was dissolved in a buffer containing 8 M urea, 1% SDS (1:1), and protease inhibitor (1:25) and resuspended by sonication, followed by centrifugation at 3,200 g for 10 min to remove insoluble particles. Thereafter, protein samples were sent for analysis at EMBL Proteomics Core Facility in Heidelberg, Germany. There, samples were subjected to an in-solution tryptic digest using a modified version of the Single-Pot Solid-Phase-enhanced Sample Preparation (SP3) protocol (Moggridge et al., 2018). Samples were added to Sera-Mag Beads (Thermo Fisher) suspended in 40 µl of a solution of 15% formic acid and ethanol solution (1:3, respectively). Protein binding was achieved by 15 min of shaking at RT, followed by SDS removal by washing four times with 200 µl of 70% ethanol. For digestion, proteins were left overnight at RT with 0.4 µg of sequencing grade modified trypsin (Promega, Madison, WI, United States) in 40 µl Hepes/NaOH, pH 8.4 in the presence of 1.25 mM TCEP and 5 mM chloroacetamide (Sigma-Aldrich). Beads were separated and washed with 10 µl of a 2% DMSO aqueous solution until the combined eluates were dried. Digested peptides were reconstituted in 10 µl of H_2_O and reacted for 1 h at RT with a TMT10plex (Thermo Fisher) label reagent (80 µg dissolved in 4 µl of acetonitrile) as previously described (Moggridge et al., 2018). TMT-labeled peptides were dried and reconstituted in 100 µl of 0.1% formic acid in H2O. After that, 5 µl (corresponding to 5% of the volume) was mixed with 5 µl of the other samples. Each sample was subjected to a reverse-phase clean-up step (OASIS) and then measured on our Lumos system using a 1 h gradient. Calculated TMT ratios were used to adjust sample volumes to achieve a 1:1 ratio (mock controls: 127L, 127H; miR-124 inhibitor: 128H, 129L, and miR-124 mimic: 130L, 130H). The combined samples were subjected to a high pH offline fractionation yielding 12 fractions (Hughes et al., 2014), each of those analyzed on a 2 h gradient on Orbitrap Fusion Lumos mass spectrometer (Thermo Fisher). Peptides were separated using an UltiMate 3000 nano RSLC system (Dionex, Thermo Fisher) equipped with a trapping cartridge (Precolumn; C18 PepMap 100, 5 mm, 300 µm i.d. × 5 μm, 100 A°) and an analytical column (ACCLAIM PEPMAP100 C18, 3 μm, 100 Å, 75 μm i.d. × 15 cm) connected to a nanospray-Flex ion source. The peptides were loaded onto the trap column (30 µl per min) using solvent A (0.1% formic acid) and eluted using a gradient from 2% to 40% Solvent B (0.1% formic acid in acetonitrile) during 2 h (0.3 µl per min). The Orbitrap Fusion Lumos was operated in a positive ion mode with a spray voltage of 2.4 kV and capillary temperature of 275°C to analyze the peptides. MS spectra with a mass range of 375–1.500 m/z were acquired in profile mode using a resolution of 120,000 [maximum fill time of 50 ms or a maximum of 4 × 10^5^ ions (automatic gain control, AGC)]. Fragmentation was triggered for 3 s cycle time for peptide-like features with charge states of 2–7 on the MS scan (data-dependent acquisition). Precursors were isolated using the quadrupole with a window of 0.7 m/z and fragmented with a normalized collision energy of 38. Fragment mass spectra were acquired in profile mode and a resolution of 30,000 in profile mode. The maximum fill time was set to 64 ms or an AGC target of 1 × 10^5^ ions. Dynamic exclusion was set to 45 s.

#### 2.12.2 Raw MS Data Processing and Analysis

MS data were analyzed using IsobarQuant (Franken et al., 2015), Mascot V2.4 (Matrix Science, London, United Kingdom) and a reverse UniProt FASTA Homo sapiens (UP000005640) database, including common contaminants. The following modifications were considered: carbamidomethyl (C, fixed), TMT10plex (K, fixed), acetyl (N-term, variable), oxidation (M, variable,) and TMT10plex (N-term, variable). Mass error tolerance for full scan MS spectra was set to 10 ppm and for MS/MS spectra to 0.02 Da. A maximum of two missed cleavages were allowed. A minimum of two unique peptides with a peptide length of at least seven amino acids and a false discovery rate below 0.01 was required on the peptide and protein level, as described (Savitski et al., 2015). From a total of 5,731 identified proteins, 3,785 proteins were quantified. The raw output files of IsobarQuant were processed using the R programming language (R Core Team, 2020). As a quality filter, we only considered proteins quantified with at least two unique peptides. Raw TMT reporter ion intensities (“signal_sum”-columns) were first corrected for batch effects using the “removeBatchEffect” function of the limma package (PMID: 25605792) and further normalized using variance stabilization normalization with the “vsn2” function of the vsn package (Huber et al., 2002). Proteins were tested for differential expression using the limma package (Supplementary Datasheet 1). The replicate information was added as a factor in the design matrix given as an argument to the “lmFit” function of limma. The *t*-value output of limma’s “topTable” function was used as an input to the “fdrtool” function of the fdrtool package (PMID 18441,000) in order to estimate *p*-values and false discovery rates (fdr) (*q*-values were used). Data normalization for each replicate can be found in Supplementary Figure S1, while the relative expression profile of the full proteome dataset is presented in Supplementary Figure S2 and listed in Supplementary Datasheet 2. Proteins were annotated as a hit with an fdr smaller 0.05 and a fold-change of at least 100% and as a candidate with an fdr below 20% and a fold-change of at least 50% (most significant results are presented in Supplementary Table S2). The full proteome dataset was classified based on cell component, biological process, and molecular function using Gene Ontology (GO) bioinformatic annotation tool PANTHER with the Overrepresentation Test (version 16.0, release 2020-12-01) (Supplementary Datasheet 3). Then, a second GO bioinformatic annotation was performed using the same parameters, but only for the most significant proteins (Supplementary Table S3). Homo sapiens GO database (DOI: 10.5281/zenodo.4495804) was used as a reference (Mi et al., 2021), with Fisher’s exact test and Bonferroni correction for multiple testing. MS proteomics raw data have been deposited in the ProteomeXchange Consortium *via* the PRIDE database^1^ (Perez-Riverol et al., 2019) partner repository with the dataset identifier PXD030315.

#### 2.12.3 Construction of the miR-124 Targeting Networks

To create the inflammatory targeting network of miR-124, the subset of 17 differently expressed proteins in microglia, identified in the proteomic analysis and classified as associated with inflammation and innate immunity, were interrogated using the online platform miRNet (Chang et al., 2020). This tool is an open-source platform that comprises eleven miRNA-target prediction databases, including miRTarBase, TarBase, miRecords, SM2mir, Pharmaco-mir, mir2Disease, PhenomiR, StarBase, Epimir, miRDB, and miRanda, mainly focusing on miRNA-target interactions. After defining the interaction tables with hsa-miR-124-3p in the Network Builder menu, the target network was generated and adjusted in the Network Viewer menu, and an image of the generated network was saved. An integrative analysis was also performed in the miRnet.ca platform, using the full proteome dataset with the KEGG database for pathway enrichment through the hypergeometric algorithm for both miRNAs and proteomic data (Supplementary Figure S3).

### 2.13 Determination of MMP-2 and MMP-9 Activity by Gelatin Zymography

MMP-2 and MPP-9 activities were determined in the extracellular media of either SWE cells and CHME3 microglia monocultures or those of cocultures by performing an SDS-PAGE zymography using 0.1% gelatin-10% acrylamide gels, under non-denaturing conditions, as previously described (Cunha et al., 2016). After electrophoresis, gels were washed for 1 h with 50 mM Tris pH 7.4, containing 2.5% Triton-X100, 5 mM CaCl_2_, and 1 µM ZnCl_2_, to remove SDS and renature the MMP species. In order to promote gelatin digestion, gels were incubated in the developing buffer (50 mM Tris pH 7.4, 5 mM CaCl_2_, and 1 µM ZnCl_2_) at 37°C overnight. Then, gels were stained with 0.5% Coomassie Brilliant Blue R-250 (Sigma-Aldrich) and destained using 30% ethanol/10% acetic acid in H_2_O (v/v) to measure enzyme activity. Image acquisition of white bands on a blue background was performed in a ChemiDoc Imaging System (Bio-Rad, Hercules, CA, United States), and further relative quantification by Image Lab analysis software (Bio-Rad). Results are representative of at least three independent experiments.

### 2.14 Silencing Dicer1 in CHME3 Microglia With siRNAs

A pool of siRNAs targeting human Dicer1 was used to silence the Dicer1 expression in CHME3 microglia (SMARTpool: ON-TARGETplus DICER1 siRNA), purchased from Dharmacon®, Thermo Fisher. Briefly, Dicer1 siRNA and the transfection factor (X-tremeGENE, Sigma-Aldrich) were gently mixed in Optimem media (Gibco, Thermo Fisher) and incubated 10 min at RT until transfection. Then, the mixture was added to the cells to perform a final concentration of 50 nM for siRNAs and 100 µM for X-tremeGENE. Timepoints of 12, 24, 48, and 72 h after transfection were tested to select the best results. Dicer-1 inhibition was successfully achieved and confirmed by western blot, and miR-124 silencing validated by RT-qPCR.

### 2.15 Protein Extraction, Quantification, and Western Blot

For western blot analysis, cells and/or EXOs were suspended in cell lysis buffer (Cell Signaling), snap-frozen, and stored at −80°C until use. Protein concentration was measured using BCA Protein Assay Kit (Pierce Biotechnology, Waltham, MA, United States), and equal amounts of protein (30 µg for EXOs and 40 µg for cells) were separated using Tris-Tricine gel, transferred into nitrocellulose membranes (Amersham, Health, Buckinghamshire, United Kingdom), and incubated in blocking buffer [5% (w/v) non-fat dried milk in Tween 20 (0.1%) tween-tris buffer saline (T-TBS)] at RT for 1 h. Membranes were incubated at 4°C overnight with the following primary antibodies diluted in blocking buffer: mouse anti-ALIX (1:1,000, Cell Signaling); goat anti-CD63 (1:1,000, Santa Cruz Biotechnology, Dallas, TX, United States); mouse anti-flotillin-1 (1:1,000, BD Biosciences); rabbit anti-Dicer (1:1,000, Cell Signaling); and mouse anti-β-actin (1:2,000, Sigma-Aldrich). Then, membranes were incubated with respective HRP-conjugated secondary antibodies also diluted in blocking buffer at room temperature for 1 h: goat anti-mouse (1:2,000); rabbit anti-goat (1:2,000); and goat-anti-rabbit (1:2,000) all from Santa Cruz Biotechnology. WesternBright Sirius® (Advansta, San Jose, CA, United States) was used as chemiluminescent substrate and signal acquired in ChemiDoc Imaging System (Bio-Rad). For relative densitometric analysis of protein bands, the Image Lab analysis software (Bio-Rad) was used.

### 2.16 EXO-Labeling With PKH67 and Incubation With CHME3 Microglia

Freshly isolated EXOs were labeled with the PKH67 Fluorescent Linker Kit (Sigma-Aldrich) to monitor the uptake of SWE-derived EXOs by CHME3 microglia, according to the manufacturer's instructions as we published previously (Pinto et al., 2017). CHME3 microglia were incubated for 24 h with PKH67-labeled EXOs suspended in fresh DMEM supplemented with 1% AB/AM. Cell fixation, additional staining, and fluorescence acquisition/analysis were performed as described above.

### 2.17 Statistical Analysis

Results of at least three independent experiments are presented as mean ± SD. Single comparisons between two conditions were done by Student’s *t*-test. In contrast, comparisons between multiple cells/conditions were accomplished by one-way ANOVA, with Bonferroni *post hoc* for multiple comparisons test. Statistical analysis was performed using GraphPad Prism 9 (GraphPad Software Inc., San Diego, CA, United States). Only *p-*values lower than 0.05 were considered statistically significant.

## 3 Results

### 3.1 Upregulation of miR-124 in SWE Cells/EXOs/EXO-Free Secretome Has No Consequences on Cell Viability or Release of Inflammatory miRNAs and Cytokines

**FIGURE 1:**
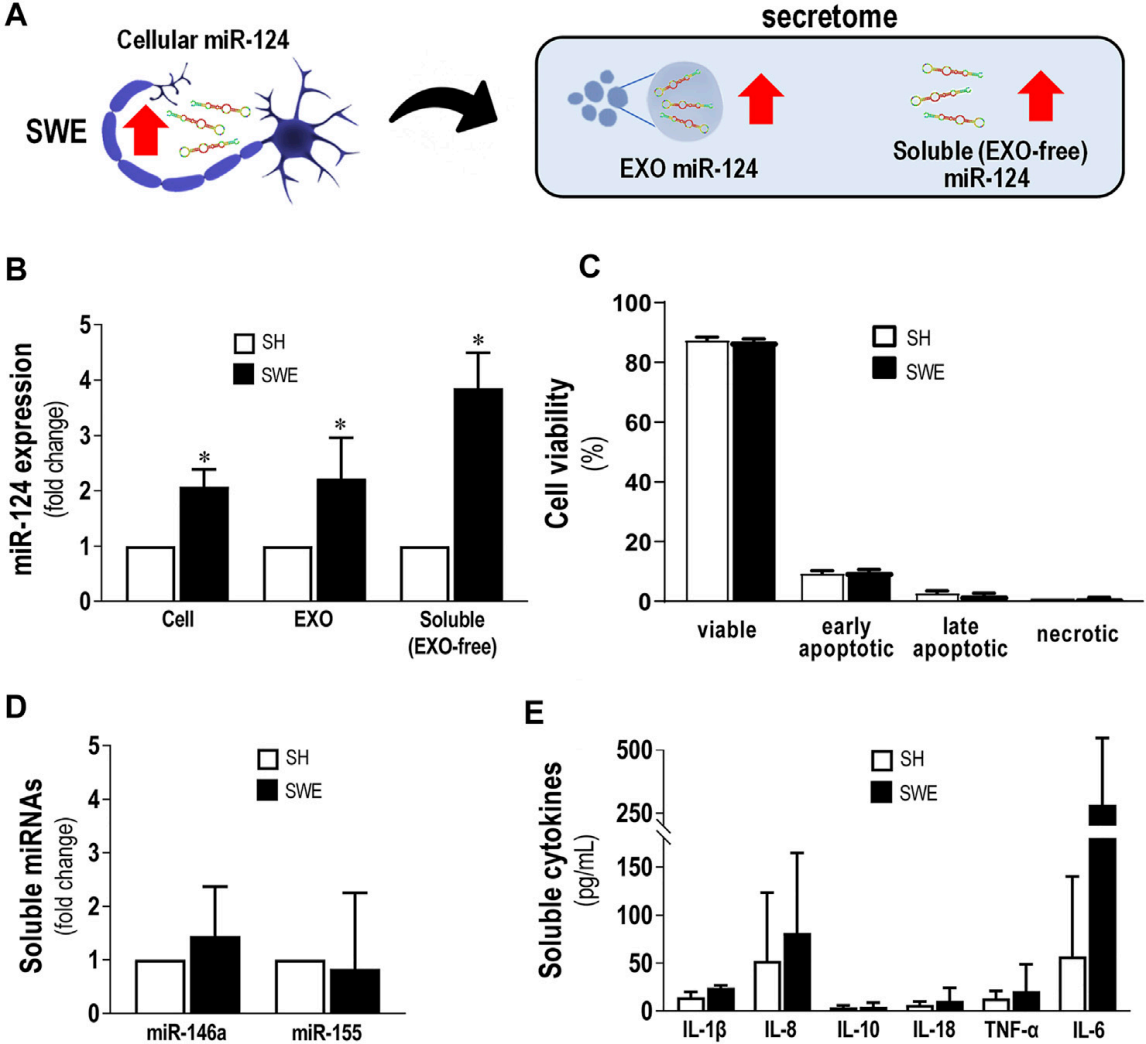
Upregulated levels of miR-124 in SWE cells do not modify cell viability or secretion of inflammatory miRNAs and cytokines. Cells were differentiated using 10 µM retinoic acid for 7 days before experiments, as described in the *Materials and Methods* section. **(A)** Schematic representation of miR-124 increase in SWE cells and their secretome, including both exosomal (EXOs) and exosome-free (EXO-free, soluble) fractions. **(B)** Cellular miR-124 expression in SH and SWE cells and their respective EXOs and EXO-free secretome. **(C)** SH and SWE cell viability by the Nexin flow cytometry assay. Four cell populations were distinguished: viable (V-PE and 7-AAD double-negative), early apoptotic (V-PE positive and 7-AAD negative), late apoptotic (V-PE and 7-AAD double-positive), and necrotic cells/cellular debris (V-PE negative and 7-AAD positive). **(D)** Levels of miR-146a and miR-155 in secretomes from SH and SWE cells. **(E)** Profile of inflammatory-associated cytokine levels in the EXO-free secretomes from SH and SWE cells using the LEGENDplex flow cytometry assay. Results are mean ± SD from at least three independent experiments. *p < 0.05 *vs*. SH levels, two-tailed Student’s *t*-test. miR, miRNA; SH, human SH-SY5Y wild-type neurons; SWE, human SH-SY5Y expressing the APP695 Swedish mutant protein; EXO, Exosomes; IL, interleukin; TNF-α, tumor necrosis factor α.

We started this study by isolating EXOs from both SH and SWE cells after 24 h incubation, by sequential ultracentrifugation, as we previously described (Fernandes et al., 2018; Garcia et al., 2021). After confirming their round shape surface morphology by TEM, and the presence of the typical EXO-associated markers ALIX, CD63, and flotillin-1 by western blot (Supplementary Figure S5), we compared the levels of miR-124 in both SH and SWE cells, as well as in their EXOs and EXO-free secretomes, as schematized in Figure 1A. The SWE cells revealed upregulated miR-124, in either cells or in their secretome fractions (*p* < 0.05; Figure 1B). Curiously, such increased miR-124 levels in SWE cells, leading to dissemination by the secretome fractions, was not reflected in changes of neuronal cell viability when compared to SH controls (Figure 1C), confirming our previous studies (Fernandes et al., 2018). Likewise, no changes were produced, in either inflammatory-associated miRNAs (inflamma-miRNAs), such as miR-146a-5p and miR-155-5p (Figure 1D), or inflammatory cytokines, such as IL-1β, IL-8, IL-10, IL-18, TNF-α, and IL-6, despite the non-significant elevation observed (Figure 1E). In summary, from all the evaluated cellular and extracellular markers, only miR-124 was consistently altered in SWE cells and in their secretome compared to SH counterparts.

Considering the reports on miR-124 properties over microglia immune function (Ponomarev et al., 2011; Yu et al., 2017; Fumagalli et al., 2018), we became interested in investigating whether such increased miR-124 release by SWE cells would have beneficial or harmful effects on the neuro-immune properties of activated microglial cells. For that purpose, we next used the human CHME3 microglia cell line stimulated with IFNγ and evaluated their inflammatory signature, in order to later assess changes induced upon the SWE-CHME3 cocultures.

### 3.2 IFNγ-Treated Microglia (IFNγ-MG) Show a Typical Pro-Inflammatory Profile With Increased Nitrosative Activity and Upregulation of Inflammatory Genes/miRNAs

**FIGURE 2:**
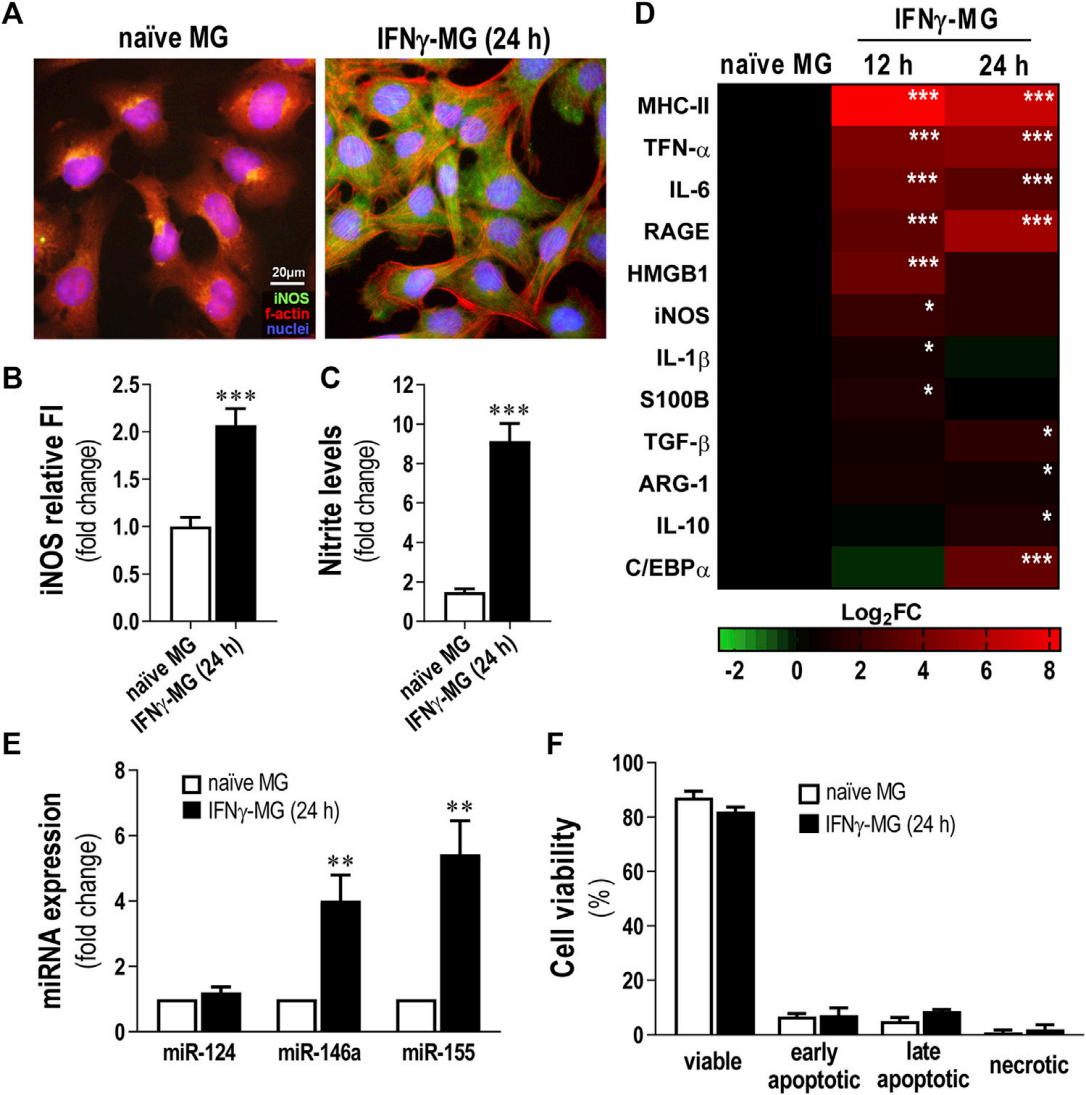
Reactive profile of CHME3 human microglia upon stimulation with IFNγ. Human microglia CHME3 were either non-treated (naïve) or stimulated with IFNγ for 12 h or 24 h and assessed for several biomarkers, as indicated in the *Materials and Methods* Section. **(A)** Representative fluorescence images of microglial iNOS (green) and f-actin (red). Cell nuclei are stained with Hoechst 33258 dye (blue). **(B)** Quantification of iNOS relative cell fluorescence intensity (FI). **(C)** Evaluation of nitrite accumulation in the cell media. **(D)** Heatmap representation of inflammatory-associated genes in microglia after 12 and 24 h of IFNγ stimulation. **(E)** Expression levels of the miRNAs associated with inflammation. **(F)** Microglia viability by Nexin flow cytometry assay. Four cell populations were distinguished: viable (V-PE and 7-AAD double-negative), early apoptotic (V-PE positive and 7-AAD negative), late apoptotic (V-PE and 7-AAD double-positive), and necrotic cells/cellular debris (V-PE negative and 7-AAD positive). Results are mean ± SD from at least three independent experiments. **p < 0.01 and ***p < 0.001 *vs*. naïve MG, two-tailed Student’s *t*-test. MG; CHME3 human microglia cells; IFNγ-MG, IFNγ-stimulated CHME3 human microglia cells; f-actin, filamentous-actin; iNOS, inducible nitric oxide synthase; miR, miRNA; h, hours; MHC-II, major histocompatibility complex–class II; TNF-α, tumor necrosis factor α; IL, interleukin; RAGE, receptor for advanced glycation end products; HMGB1, high mobility group box protein 1; S100B, S100 calcium-binding protein B; TGF-β, transforming growth factor β; ARG-1, arginase-1; C/EBPα, CCAAT enhancer binding protein α.

Significant IFNγ levels were previously identified in AD patients, from mild to severe stages (Belkhelfa et al., 2014). IFNγ has been indicated to be involved in microglia reactivity to Aβ (Meda et al., 1995) and to cause the production of inflammatory cytokines (Abbas et al., 2002; Rock et al., 2005). Herein, we established a human model of activated microglia using the CHME3 human microglia cell line and the stimulation with 50 ng/ml of IFNγ (IFNγ-MG) for 12 and 24 h, as previously described (Spencer et al., 2016). Next, we evaluated iNOS immunostaining, monitored nitrite release, and analyzed inflammatory gene expression (Figures 2A–D). We also assessed the expression levels of miR-124, as well as of miR-146a and miR-155 in IFNγ-MG, considering their neuro-immune relevance that we previously explored (Fernandes et al., 2018; Garcia et al., 2021) (Figure 2E). Evaluation of microglia viability upon IFNγ stimulus was also examined (Figure 2F). We confirmed the release of EXOs into the secretome and the presence of their usual protein markers after incubation with IFNγ (Supplementary Figure S5). Our results showed that IFNγ promotes inflammatory polarization of CHME3 microglia, noticed by the increase in iNOS immunofluorescence intensity (*p* < 0.001, Figure 2B) and the six-fold elevation of nitrite accumulation in the cell media at the same timepoint (*p* < 0.001, Figure 2C).

Regarding inflammatory gene expression, IFNγ induced significant overexpression of Major histocompatibility complex (MHC)-II, TNF-α, IL-6, and RAGE (*p* < 0.001), either after 12 or 24 h of stimulation (Figure 2D). Other inflammatory genes such as HMGB1 and iNOS peaked after 12 h of treatment with IFNγ (*p* < 0.001 and *p* < 0.05, respectively). On the contrary, genes involved in the pro-regenerative microglial polarization, as TGF-β and IL-10, only peaked (*p* < 0.01 and *p* < 0.05, respectively) after 24 h of IFNγ stimulation. In addition, we evaluated the transcription of C/EBPα, which, apart from being a marker of disease-associated microglia contributing to caspase activation and apoptosis (Pan et al., 2013), also constitutes a target of miR-124 in microglia (Ponomarev et al., 2011). We found that C/EBPα was overexpressed after 24 h of IFNγ treatment (*p* < 0.001), suggesting such a timepoint as the most adequate to further evaluate the effects of the SWE-derived miR-124 in coculture. Concerning the miRNA signature, while miR-146a and miR-155 were highly boosted (*p* < 0.01 for both) in IFNγ-MG, miR-124 levels did not change from those found in naïve microglia (Figure 2E). Despite the pro-inflammatory profile of IFNγ-MG, our results demonstrated that even after 24 h of IFNγ stimulation, CHME3 viability was not significantly affected (Figure 2F), validating the use of such stimulation period in subsequent studies.

### 3.3 Elevated miR-124 Levels in SWE Cells Determine their Enrichment in IFNγ-MG and Reduce Microglial Nitrosative Activity and Neuronal Mitochondrial Membrane Potential

**FIGURE 3:**
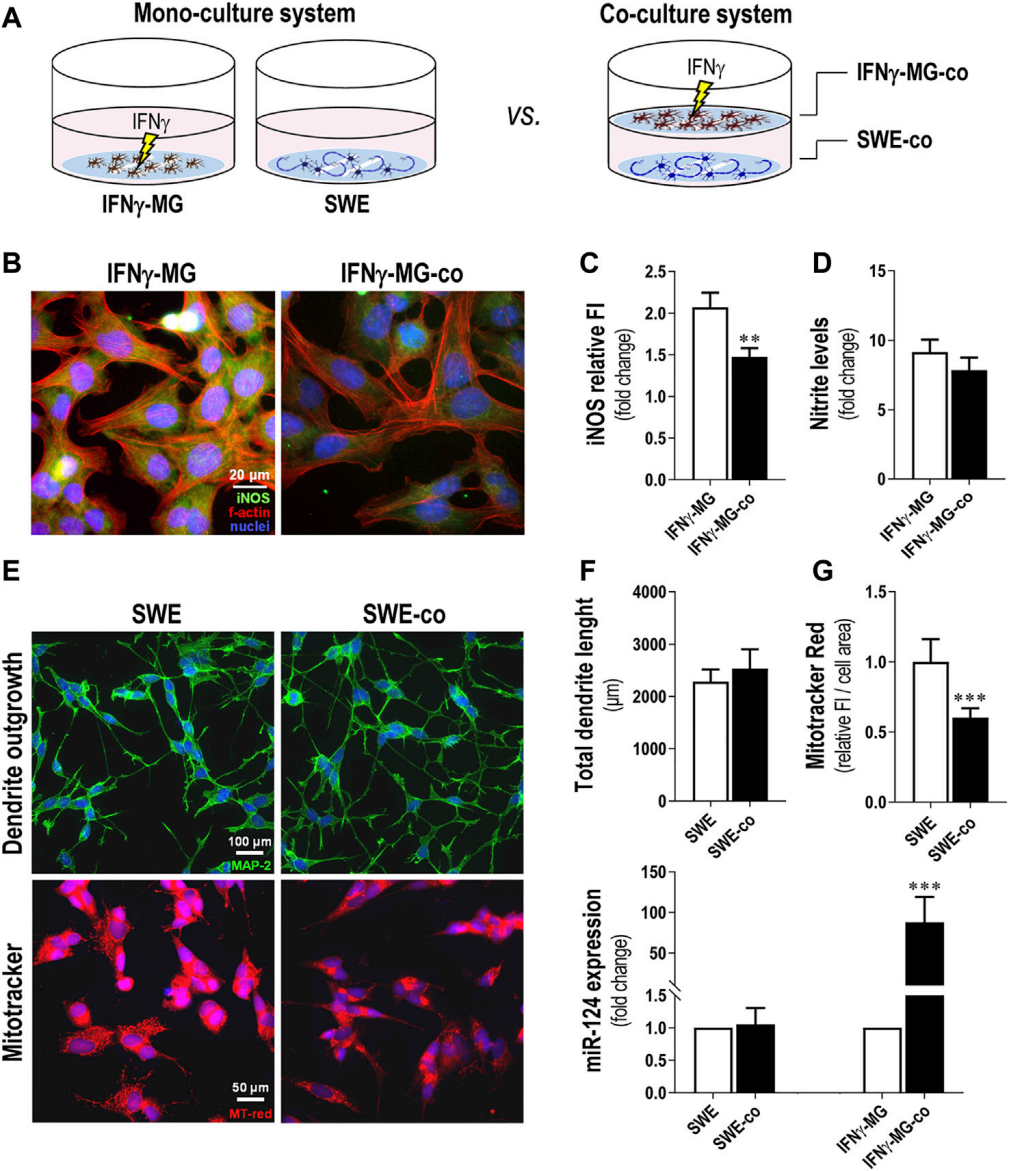
Coculture of IFNγ-MG with SWE cells reduces neuronal MitoTracker intensity, while increasing microglial iNOS immunostaining and miR-124 expression levels. SWE cells were differentiated using 10 µM retinoic acid for 7 days before experiments, and CHME3 microglia were pretreated with IFN-γ for 24 h before coculture, as detailed in the *Materials and Methods* section. **(A)** Schematic representation of the coculture establishment. **(B)** Representative fluorescence images of iNOS (green) and f-actin (red) in MG monocultures and cocultures with SWE cells. Cell nuclei were stained with Hoechst 33258 dye (blue). **(C)** Comparison of MG-iNOS relative fluorescence intensity (FI) and **(D)** nitrite accumulation in the cell media when in the monocultures and coculture system with the SWE cells. **(E)** Representative fluorescence images of MAP-2 (green) and MitoTracker (red) in MG monocultures and cocultures with SWE cells. **(F)** Comparison of total dendrite length and **(G)** MitoTracker fluorescence intensity when in the monocultures and the coculture system with the SWE cells. **(H)** Comparison of the miR-124 expression levels in SWE and CHME3 cells monocultures *vs*. cocultures. Results are mean ± SD, obtained from at least three independent experiments. **p < 0.01 and ***p < 0.001 *vs*. respective monocultured cells, two-tailed Student’s *t*-test. MG, CHME3 human microglia cells; IFNγ-MG, IFNγ-stimulated CHME3 human microglia cells; SWE, human SH-SY5Y cells expressing the APP695 Swedish mutant protein; co, cocultures; iNOS, inducible nitric oxide synthase; miR, miRNA.

After characterization of the microglia response to IFNγ, we established a neuronal-microglia coculture with SWE neuroblastoma cells and IFNγ-MG, using the same procedure we previously described (Fernandes et al., 2018). The IFNγ stimulation of microglia was performed during the monoculture and before the coculture with the SWE cells. Therefore, we compared the effects of the cocultures *versus* the monocultures for each cell type, that is SWE and IFNγ-MG cells, as schematized in Figure 3A. We noticed that iNOS immunostaining in the IFNγ-MG after the coculture with the SWE cells (IFNγ-MG-co) was significantly inhibited (*p* < 0.01) when compared with their monocultures (Figures 3B,C). Even so, such an effect was not enough to be translated into a significant reduction in the nitrite accumulation, only slightly reduced (Figure 3D). SWE cells were also affected by the presence of IFNγ-MG. Despite no alterations in the total dendrite length (Figure 3E, top panels, Figure 3F), we observed a marked decline (*p* < 0.001) in MitoTracker Red intensity when SWE cells were cocultured with IFNγ-MG (SWE-co) relatively to those in monoculture (Figure 3E, bottom panels, Figure 3G), suggesting a metabolic shift by the presence of IFNγ-MG cells.

To investigate if miR-124 modified neuron-microglia dynamics, we compared the miR-124 expression levels in both SWE-co and IFNγ-MG-co *versus* the respective monocultured counterparts (Figure 3H). Intriguingly, results indicate that while the miR-124 levels in SWE cells remained unchanged regardless of IFNγ-MG presence, the microglial miR-124 levels were highly boosted (nearly 90-fold, *p* < 0.001) in the presence of SWE cells *versus* the IFNγ-MG monocultures. Such data suggest the involvement of neuronal cells in the regulation of miR-124 levels in microglia.

### 3.4 miR-124 Inhibitor Promotes SWE Cell Demise and RAGE Overexpression, While the Mimic Reduces Neuronal Stress Biomarkers, With the Corresponding miR-124 Levels Being Recapitulated in the Secretome

**FIGURE 4:**
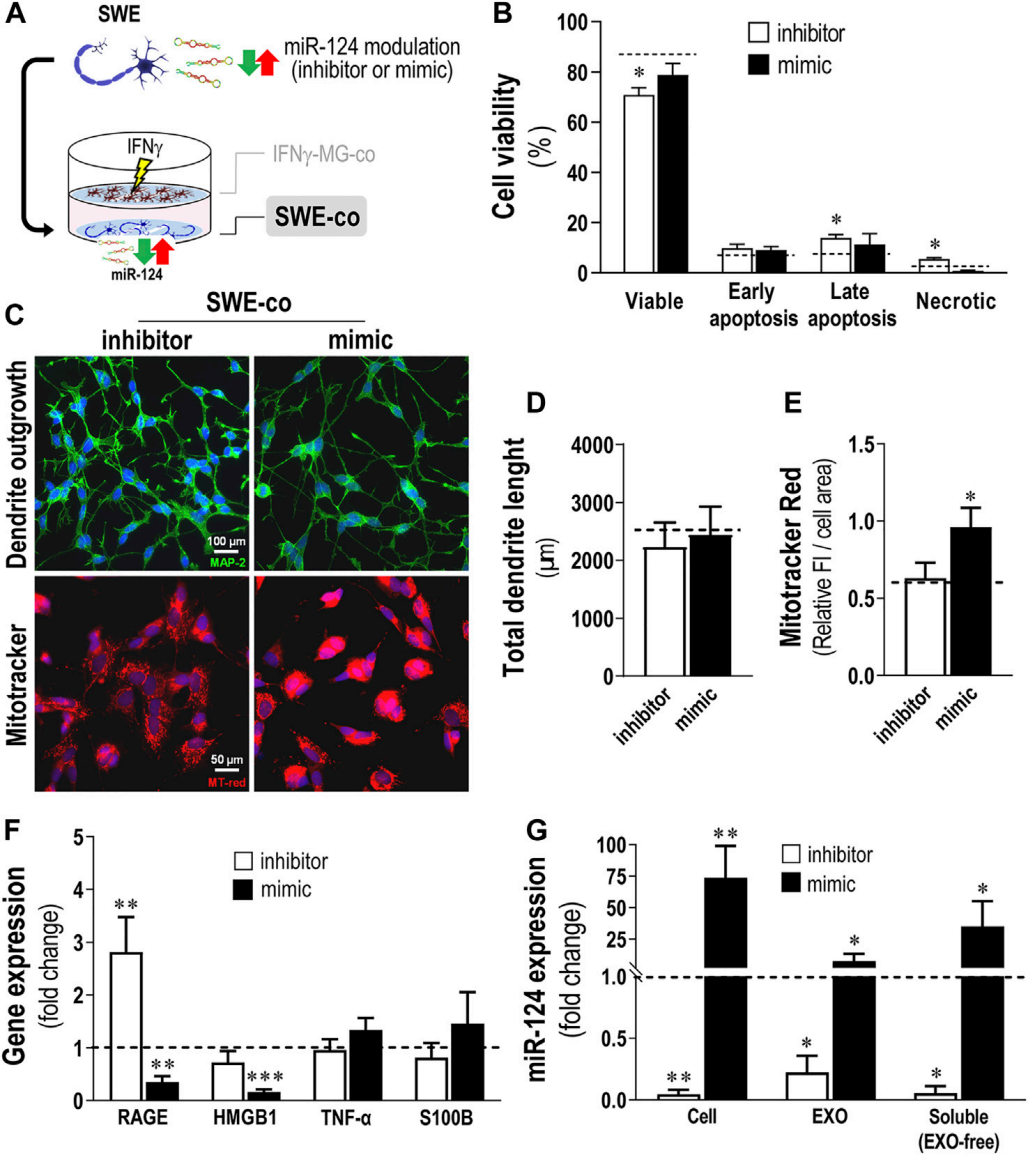
Inhibition of miR-124 in SWE cells increases cell death and RAGE gene expression, while the mimic enhances MitoTracker Red intensity and miR-124 cargo in cells and their secretomes, together with a reduction of stress-associated genes, upon coculture with IFNγ-MG. After differentiation, SWE cells were transfected with miR124 inhibitor and mimic, while CHME3 microglia (MG) were treated with IFNγ for 24 h before coculture (IFNγ-MG), as detailed in *Materials and Methods*. **(A)** Schematic representation of the miR-124 modulation, followed by the coculture establishment with IFNγ-MG. **(B)** Cell viability of SWE cells in coculture with IFNγ-MG, assessed by Nexin flow cytometry assay. Four cell populations were distinguished: viable (V-PE and 7-AAD double-negative), early apoptotic (V-PE positive and 7-AAD negative), late apoptotic (V-PE and 7-AAD double-positive), and necrotic cells/cellular debris (V-PE negative and 7-AAD positive). **(C)** Representative fluorescence images of MAP-2 (green) and MitoTracker (red) in SWE cells upon coculture with IFNγ-MG, **(D)** respective evaluation of total dendrite length, and **(E)** quantification of MitoTracker fluorescence intensity (FI). **(F)** Inflammation-associated gene expression levels in SWE cells upon coculture with IFNγ-MG. **(G)** Quantification of miR-124 expression levels in SWE cell monocultures, as well as exosomes (EXOs) and exosome-free (EXO-free, soluble) secretome, 24 h after transfection with the miR-124 inhibitor and mimic *vs*. respective mock controls (dashed line). Results are mean ± SD from at least three independent experiments. *p < 0.05, **p < 0.01, and ***p < 0.001 *vs*. mock control (dashed line), one-way ANOVA with the Bonferroni *post hoc* test. SWE, human SH-SY5Y expressing the APP695 Swedish mutant protein; IFNγ-MG, IFNγ-stimulated CHME3 human microglia cells; co, cocultured; miR, miRNA; RAGE, receptor for advanced glycation end products; HMGB1, high mobility group box 1; TNF-α, tumor necrosis; S100B, S100 calcium-binding protein B; EXO, exosomes.

To evaluate the consequences of changing the miR-124 expression levels in the SWE cells when in coculture with IFNγ-MG, we used the neuronal transfection with the miR-124 inhibitor and mimic (Figure 4A). Viability of SWE-co after 24 h was slightly but significantly decreased by the miR-124 inhibitor (Figure 4B; *p* < 0.05), with late apoptotic and necrotic increased events (*p* < 0.05). Such cytotoxic effect upon miR-124 inhibition was triggered by the presence of IFNγ-MG because no cytotoxic effects were previously detected in SWE monocultures (Garcia et al., 2021).

Many reports support that miR-124 is deeply involved in the regulation of dendrite outgrowth (Yu et al., 2008; Xue et al., 2016; Garcia et al., 2021). In addition, miR-124 was described as a master regulator of neuronal mitochondria activity and localization (Yardeni et al., 2018), capable of inhibiting mitochondrial apoptotic pathways (Xu et al., 2019). Herein, we evaluated how different levels of miR-124 affected the dendrite outgrowth and MitoTracker intensity in SWE-co cells after exposure to IFNγ-MG. Unexpectedly, no significant changes were observed in the total dendrite length, with either the inhibition or the overexpression of miR-124 (Figure 4C, top panels, Figure 4D). However, our results showed a significant boost in the MitoTracker Red intensity upon the miR-124 mimic *versus* mock control (dashed line) but no changes upon the inhibitor (*p* < 0.05, Figure 4C, bottom panels, Figure 4E). Remarkably, such upregulation of miR-124 by the mimic led to a recovery of the MitoTracker intensity (shown to be decreased with the coculture, see Figure 3G), reestablishing the original values observed in the SWE monocultures.

These effects of miR-124 over the mitochondrial activity of SWE-co cells revealed a potential role also in the neuron-microglia oxidative signaling, which is intrinsically associated with inflammation (Chen et al., 2012; Zarrouk et al., 2012). To explore such a link, we selected a subset of inflammatory-associated genes that we previously identified to be upregulated in SWE cells (Fernandes et al., 2018) and evaluated their expression upon miR-124 modulation (Figure 4F). Indeed, results indicate that different miR-124 levels promote disparate effects on SWE-co cells, with the inhibitor inducing RAGE overexpression (*p* < 0.01) and the mimic repressing the expression of both RAGE (*p* < 0.01) and its ligand HMGB1 (*p* < 0.001). Despite the tendency to increase upon the miR-124 mimic, neuronal TNF-α and S100B were not significantly affected by the miR-124 modulation.

Besides confirming the miR-124 modulation consequences in the SWE-co cells, we also assessed whether it was reflected in their EXOs and EXO-free secretome fractions (Figure 4G). SWE-co cells showed an impressive reduction of 95% with the inhibitor, while the mimic caused a 70-fold increase in miR-124 levels (*p* < 0.01, Figure 4G). Such cellular decrease/increase of miR-124 levels was recapitulated in SWE-co EXOs and EXO-free secretome (*p* < 0.05, in both), confirming a passive diffusion of the neuronal miR-124 into the secretome, previously reported by us (Garcia et al., 2021). In brief, we showed that miR-124 modulation influences SWE-co cell viability, mitochondria dynamics, RAGE-HMGB1 axis, and the amount of miR-124 released into EXOs and EXO-free secretome. Next, we intended to explore the paracrine consequences of modulating neuronal miR-124 levels for IFNγ-MG-co cells.

### 3.5 miR-124-Loaded SWE Cells Regulate the Activation State of IFNγ-MG, While miR-124 Depletion Further Enhances Cell Reactivity, Inflammatory Genes, and miRNAs

**FIGURE 5:**
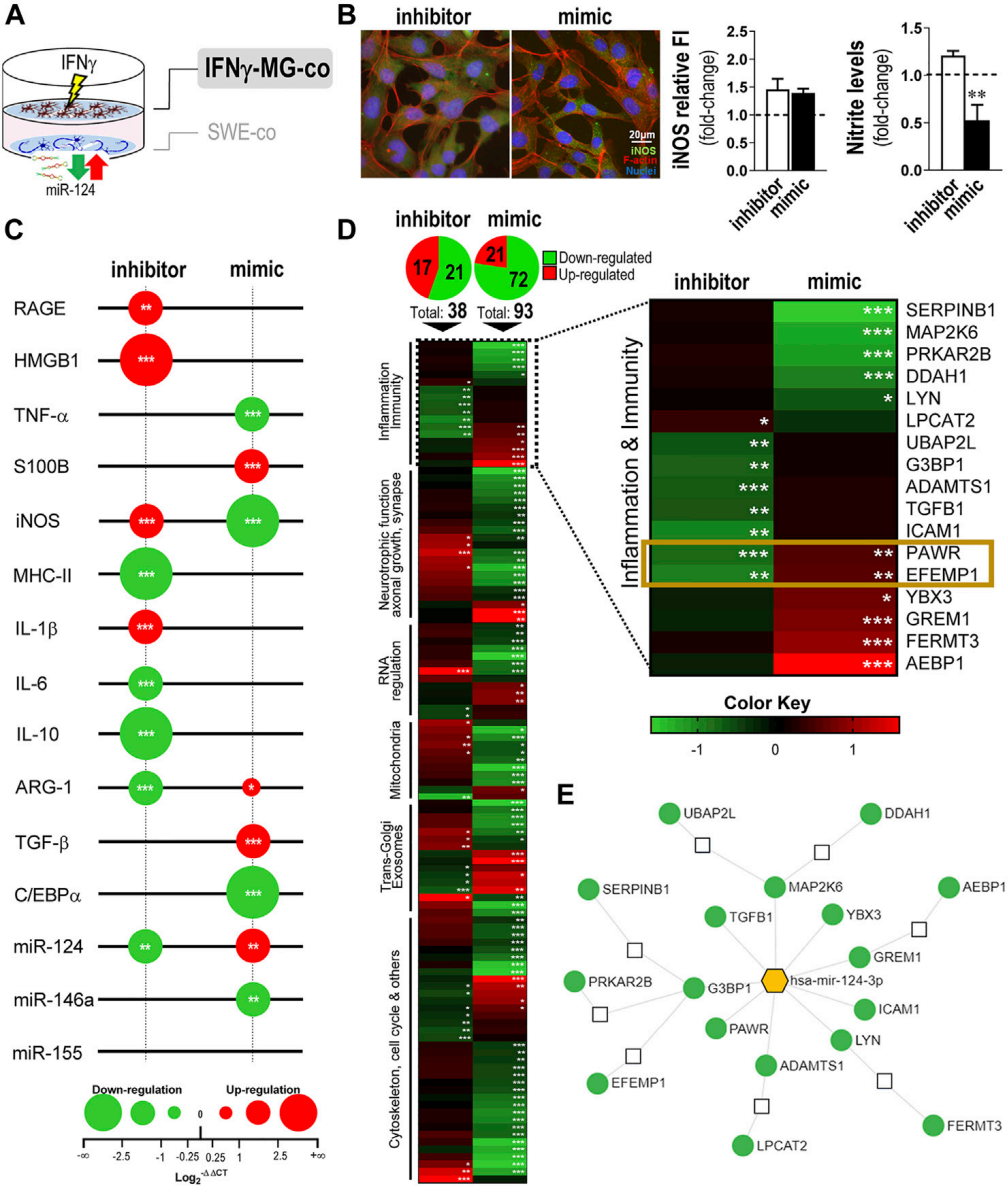
Modulation of miR-124 expression levels in SWE cells changes the reactive and proteomic profile in human IFNγ-MG upon established cocultures. After differentiation, SWE cells were transfected with miR124 inhibitor and mimic, while CHME3 microglia were pre-treated with IFNγ for 24 h (IFNγ-MG) before the coculture, as detailed in *Materials and Methods*. **(A)** Schematic representation of the SWE/IFNγ-MG coculture and subsequent indication on microglia effects. **(B)** Representative fluorescence intensity (FI) of iNOS (green) and f-actin (red) in IFNγ-MG cells with quantifications of iNOS relative to FI and nitrite accumulation in cell media, right. Nuclei were stained with Hoechst 33258 dye (blue). **(C)** Bubble plot showing the changes in inflammatory-associated gene/miRNA expression of IFNγ-MG after coculture with inhibitor- and mimic-treated SWE cells. The color of the bubbles indicates downregulated genes (green) or upregulated genes (red), and the size represents the magnitude of each down/upregulation. **(D)** Heatmap representation of 117 proteins identified and quantified by mass spectrometry-based proteomic analysis of IFNγ-MG cells cocultured with SWE (inhibitor *vs*. mimic). A subset of 17 proteins related to innate function and inflammation was highlighted based on their Gene Ontology (GO) classification. Colors indicate downregulated proteins (green) or upregulated proteins (red), and the intensity of each color represents the relative abundance of the indicated protein. Significant proteomic data are detailed in Supplementary Table S2. **(E)** Targeting network of miR-124 for each of the 17 highlighted proteins, created in the “miRnet.ca” platform. Results are mean ± SD from at least three independent experiments. *p < 0.05, **p < 0.01, and ***p < 0.001 *vs*. IFNγ-MG cocultured with SWE mock control, one-way ANOVA with the Bonferroni *post hoc* test. SWE, human SH-SY5Y expressing the APP695 Swedish mutant protein; MG, CHME3 human microglia cells; IFNγ-MG, IFNγ-stimulated CHME3 human microglia cells; co, cocultured; miR, miRNA; f-actin, filamentous-actin; iNOS, inducible nitric oxide synthase; RAGE, receptor for advanced glycation end products; HMGB1, high mobility group box protein 1; TNF-α, tumor necrosis factor α; S100B, S100 calcium-binding protein B; iNOS, inducible nitric oxide synthase; MHC-II, major histocompatibility complex–class II; IL, interleukin; ARG-1, arginase-1; TGF-β, transforming growth factor β; C/EBPα, CCAAT enhancer binding protein α.

In this section, we deeply investigated the consequences of IFNγ-MG of inhibiting/overexpressing miR-124 levels in SWE cells (Figure 5A). First, we analyzed the effects on nitrosative activity, which showed no alterations in microglia iNOS immunostaining, despite the significant reduction in nitric oxide (NO) production upon miR-124 mimic modulation (Figure 5B, *p* < 0.01). Such NO reduction indicates a direct consequence of neuronal miR-124 overexpression on IFNγ-MG immune properties, previously explored (Ponomarev et al., 2011).

Then, we analyzed the expression of a set of inflammation-associated genes and miRNAs in IFNγ-MG after coculture with SWE cells treated with an inhibitor/mimic of miR-124 (Figure 5C). Results confirmed the influence of neuronal miR-124 changes by the inhibitor and mimic on the microglia disparate gene expression profiles. The paracrine signaling by SWE miR-124 inhibition led to microglial overexpression of RAGE (*p* < 0.01), as well as of HMGB1, iNOS, and IL-1β (*p* < 0.001), while repressing microglial IL-10 and ARG-1 gene expression levels (*p* < 0.001), thus hampering pro-resolving cascades. In contrast, SWE miR-124 upregulation by the mimic inhibited the microglial expression of TNF-α and iNOS, disrupting part of the pro-inflammatory signature stimulated by IFNγ, described in Figure 2D. On top of this, the miR-124 mimic in neurons influenced the pro-resolving signature in IFNγ-MG, with significant overexpression of ARG-1 (*p* < 0.05) and TGF-β (*p* < 0.001). Regarding the inflammation-associated miRNAs, their microglial levels differed according to the dissemination of miR-124 levels from the SWE cells. While the inhibition of miR-124 in SWE cells caused its depletion (*p* < 0.01), the mimic upregulated miR-124 in IFNγ-MG cells (*p* < 0.01) with marked repression of its direct target, the C/EBPα (*p* < 0.001). Furthermore, a significant reduction (*p* < 0.01) was found in the AD-associated miR-146a upon the mimic action on SWE cells (Wang et al., 2016; Ansari et al., 2019) (Figure 5C). Surprisingly, no changes were observed in the miR-155 expression, despite being the most upregulated miRNA by the IFNγ stimulus (Figure 2E).

To deeper investigate the consequences of the neuronal miR-124 modulation on the neighboring IFNγ-MG cells, we performed a proteomic analysis in these cells after coculture with SWE treated with inhibitor and/or mimic (Figure 5D and Supplementary Figure S2). Remarkably, from a total of 3,785 quantified proteins in IFNγ-MG, more than a hundred of them were significantly affected by the SWE levels of miR-124 (Figure 5D, top and detailed in Supplementary Table S2). Curiously, the miR-124 mimic produced a higher impact in IFNγ-MG with 93 differently expressed proteins (DEPs) compared to the 38 DEPs upon the miR-124 inhibitor. From those 93 mimic-dependent DEPs, the large majority (72) was downregulated. This finding indicates that miR-124 overexpression in SWE cells has a predominant effect on inhibiting IFNγ-MG protein expression.

Gene Ontology (GO) annotation for the cell component of DEPs clearly indicates an enrichment on neuron-specific terms, including “synapse,” “dendrite,” or “growth cone,” despite showing enrichment in “extracellular EXOs” and “secretory vesicles” (Supplementary Table S3). Even so, those terms were not dominant in the full GO annotation done with the full proteome dataset of IFNγ-MG (Supplementary Datasheet 3). In this part, we specifically focused on the immune/inflammatory changes in microglia upon miR-124 modulation. Many protein IDs from the full proteome dataset were annotated in the GO analysis as involved in “immune regulation,” “inflammatory response,” and “response to stimulus” (Supplementary Datasheet 3). Then, we filtered the 17 most differentially expressed proteins changed upon the miR-124 inhibitor, the mimic, or simultaneously in both conditions (Figure 5D, right). From these, PRKC apoptosis WT1 regulator (PAWR) and EGF-containing fibulin-like extracellular matrix protein 1 (EFEMP1) were the only two proteins differently expressed with the inhibitor and/or the mimic, showing a direct response to the neuronal miR-124 levels. Both proteins were significantly co-inhibited in microglia upon neuronal miR-124 inhibition (PAWR, *p* < 0.001, and EFEMP1, *p* < 0.01) and co-expressed upon neuronal miR-124 overexpression (*p* < 0.01, for both).

Most of the significant effects were mediated by the miR-124 mimic, including the reduction (*p* < 0.001) of leukocyte elastase inhibitor (SERPINB1) and dual-specificity mitogen-activated protein kinase kinase 6 (MAP2K6), limiting the pro-inflammatory signaling. Additionally, the mimic increased the expression of: 1) Y-box-binding protein 3 (YBX3), a known Granulocyte-macrophage colony-stimulating factor (GM-CSF) regulator (*p* < 0.05) (Coles et al., 1996); 2) gremlin-1 (GREM1) (*p* < 0.001), involved in monocyte chemotaxis (Müller et al., 2014); and 3) fermitin family homolog 3 (FERMT3) (*p* < 0.001), an essential player in hematopoietic cell adhesion and potential NF-kB repressor (Wang et al., 2008; Svensson et al., 2009). In contrast, the neuronal inhibition of miR-124 led to a significant decrease in the transforming growth factor beta-1 proprotein (TGFB1) (*p* < 0.01), a direct precursor of the anti-inflammatory cytokine TGF-β, as an example.

Finally, we used a miRNA functional analysis and system biology network database “miRnet.ca” (Chang et al., 2020) to create a targeting map of miR-124 using the subset of the 17 DEPs involved in microglia innate function (Figure 5E). We found that nine DEPs are direct targets of miR-124, while the remaining seem to be indirectly affected through pathways influenced by the direct targets. In addition, we confirmed the impact of miR-124 in the full proteome dataset of IFNγ-MG using the same miRnet.ca database (Supplementary Figure S3). Remarkably, miR-124-3p was confirmed as the miRNA better explaining the full proteomic changes detected in IFNγ-MG, through the predicted involvement of several transcription factors, such as C/EBPα, NFATc1, or STAT3, which also constitute major miR-124 targets. Our coculture system was more effective in the identification of relevant microglial proteomic data compared to other studies using monoculture (Ahuja and Lazar, 2021), despite the differences to more advanced experimental models of microglia (Svoboda et al., 2019) (Supplementary Figure S4). Nevertheless, the sum of these data elucidates the multi-targeting power of neuronal miR-124 over the microglial proteome.

### 3.6 Secretion of MMP-2 and MMP-9 Decreases When IFNγ-MG is Cocultured With miR-124-Loaded SWE Cells

**FIGURE 6:**
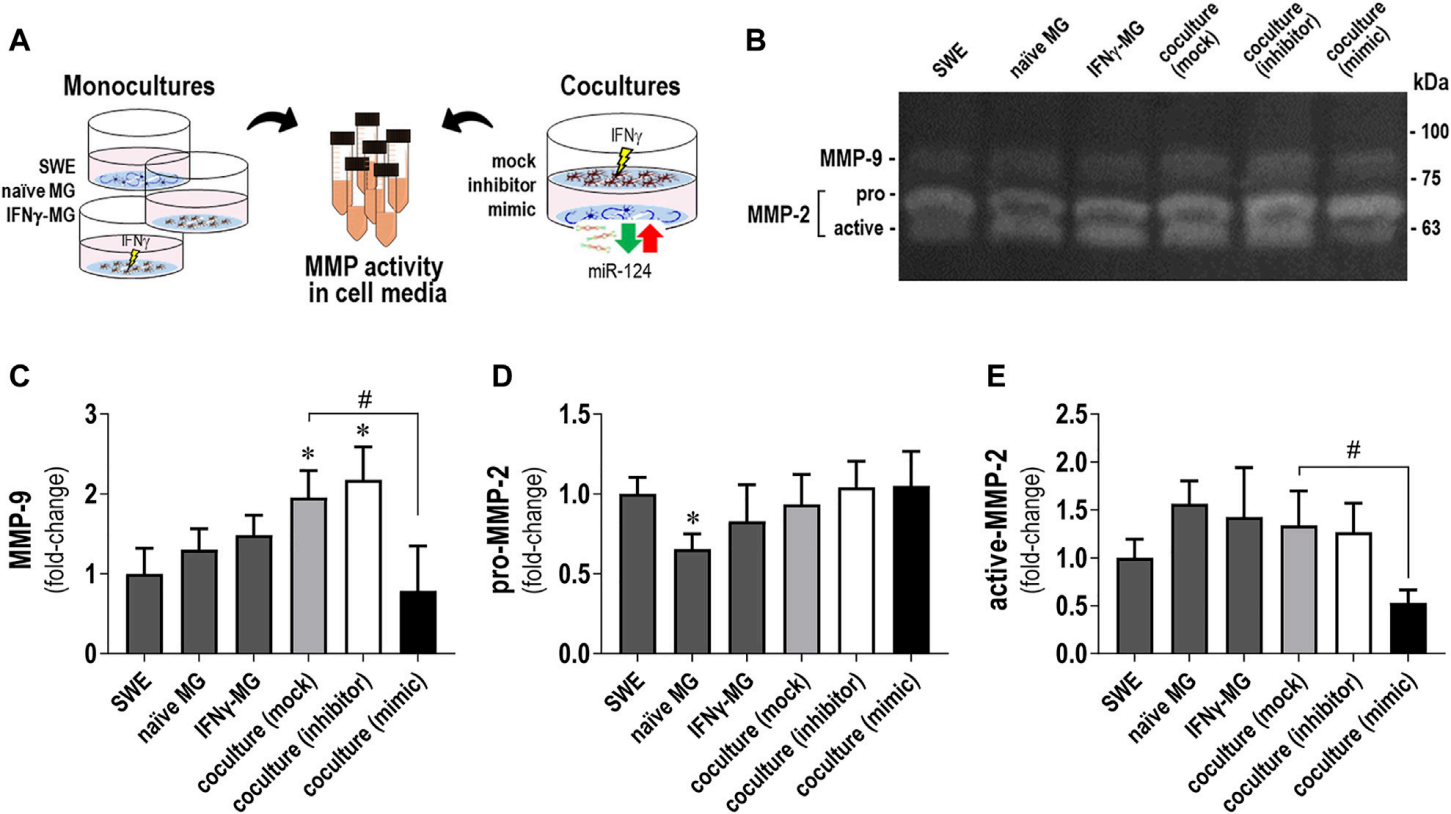
Changes in MMP-2 and MMP-9 activities by gelatin zymography in secretomes from SWE and IFNγ-MG cells, in either monocultures or cocultures, are determined by miR-124 expression levels. Cell media from the SWE and IFNγ-MG monocultures and cocultures were collected and analyzed for matrix metalloproteinase (MMP) gelatinase activities, as described in *Materials and Methods*. **(A)** Schematic representation of the experimental conditions (in either monoculture or coculture) and cell media collection. **(B)** Representative images of the gelatin zymography assay showing the gelatinolytic bands of MMP-9 and MMP-2. Discrimination between the pro- and active forms was only possible for MMP-2. **(C)** Quantification of gelatinase activity MMP-9 (±80–90 kDa), **(D)** pro-MMP-2 (72 kDa), and **(E)** active-MMP-2 (62 kDa). Results are mean ± SD from at least three independent experiments. *p < 0.05 *vs*. SWE cell secretome, one-way ANOVA with the Bonferroni *post hoc* test; #*p* < 0.05 *vs*. coculture with the SWE mock, two-tailed Student’s *t*-test. SWE, human SH-SY5Y cells expressing the APP695 Swedish mutant protein; MG, CHME3 human microglia cells; IFNγ-MG, IFNγ-stimulated CHME3 human microglia cells.

Matrix metalloproteinase (MMP) dysregulation is implicated in microglia activation, inflammation, and neurodegenerative diseases, such as AD (Könnecke and Bechmann, 2013). Previous studies have shown that low levels of miR-124 upregulated MMP-2 and MMP-9 in bladder cancer cells and favored their invasion (Xu et al., 2013). Herein, the GO analysis for IFNγ-MG-co revealed a 4.4-fold-enrichment in extracellular matrix proteins (Supplementary Table S3). Considering these aspects, we next evaluated the activity of MMP-2 and MMP-9, described to play multiple roles in AD (Candelario-Jalil et al., 2011; Wang et al., 2014; Ringland et al., 2021). We collected cell media from monocultures (SWE, naïve MG, and IFNγ-MG) and SWE/IFNγ-MG cocultures (with mock, inhibitor, and mimic of miR-124) as schematized (Figure 6A). Then, we evaluated the gelatinase activity of MMP-2 and MMP-9 using gelatin zymography under non-denaturation conditions (Figure 6B). While no significant differences were observed between monocultures, SWE/IFNγ-MG cocultures produced significant increases in MMP-9 activity upon transfection with mock and miR-124 inhibitor (*p* < 0.05 for both) compared to the SWE monoculture (Figure 6C). Oppositely, the coculture with miR-124-loaded SWE cells (mimic) led to a significant decrease (*p* < 0.05) in the MMP-9 activity compared to the mock coculture, with values close to those observed in the SWE cell monocultures (Figure 6C). Regarding MMP-2, we could distinguish between the pro- (72 kDa) and active (62 kDa) forms in our different experimental conditions (Figures 6D,E). Concerning the monocultures, we observed a reduced pro-MMP-2 accumulation in the secretome from naïve MG relative to that of SWE cells (*p* < 0.05, Figure 6D). Interestingly, the upregulation of miR-124 in the SWE cells and their coculture with IFNγ-MG determined a reduction of the active MMP-2 accumulation (Figure 6E), not observed for the pro-MMP-2. Such deactivation of the MMP-2 and MMP-9 may represent an additional benefit of miR-124 overexpression, which apparently works as an inhibitor reducing their proaggregatory and inflammatory influence in AD.

### 3.7 Elevation of miR-124 in Dicer1-Silenced IFNγ-MG in the Presence of miR-124-Loaded SWE Neurons Indicate Secretome-Mediated Signaling Between Cells

**FIGURE 7:**
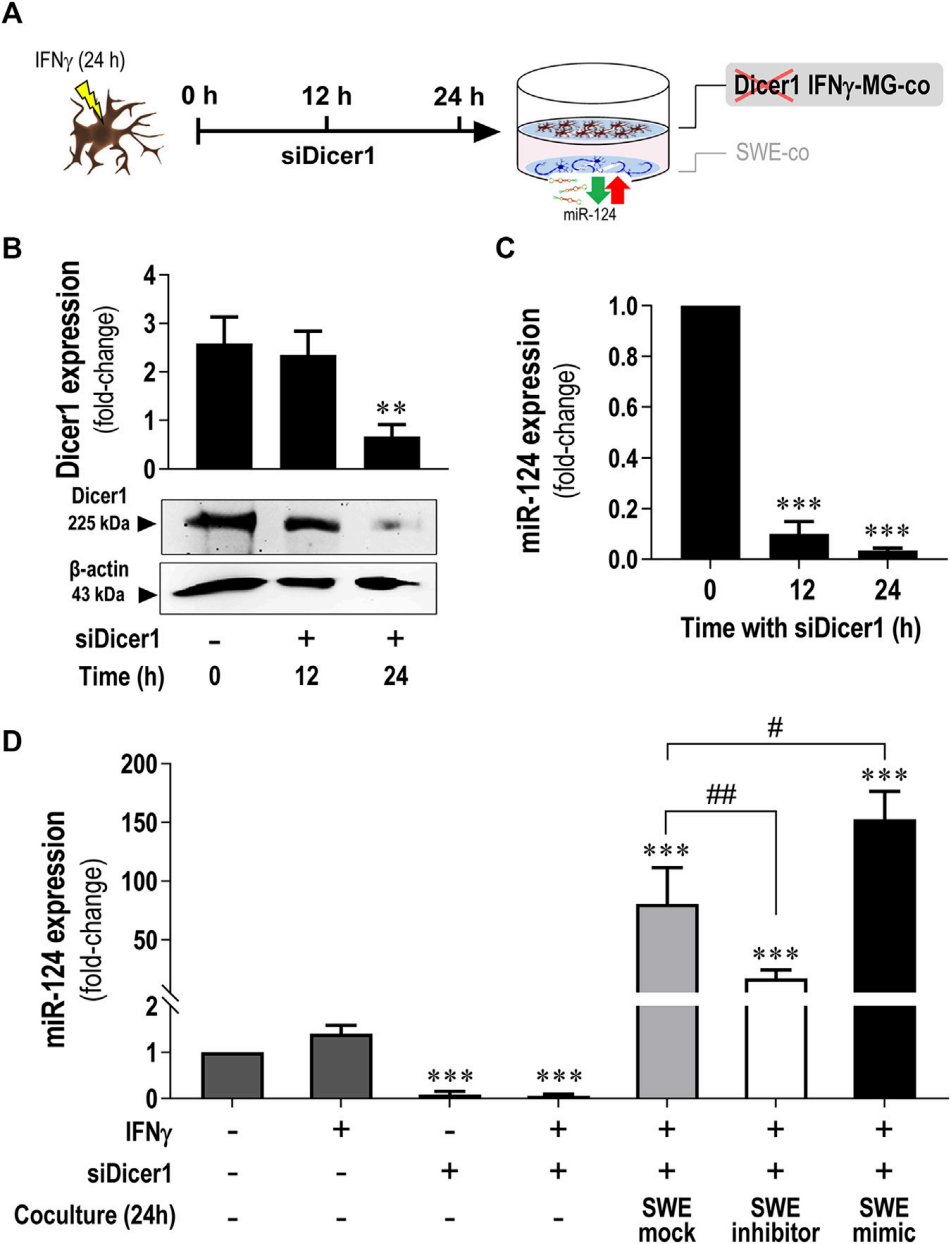
Increased miR-124 expression levels are present in Dicer1-silenced IFNγ-MG upon established cocultures with the SWE cells modulated for miR-124. After differentiation, SWE cells were transfected with mock, inhibitor, and mimic of miR-124, while CHME3 microglia were pre-treated with IFNγ (IFNγ-MG) before transfection with Dicer1 siRNAs, as detailed in *Materials and Methods*. **(A)** Schematic representation of siRNA-mediated Dicer1 silencing in IFNγ-MG cells, followed by coculture with miR-124 modulated SWE cells. **(B)** Representative western blot images and relative densitometric quantification of Dicer1 expression in IFNγ-MG after 0 (naïve), 12, and 24 h of Dicer1 siRNA incubation. **(C)** miR-124 expression levels in IFNγ-MG after 0 (naïve), 12, and 24 h of Dicer1 siRNA incubation. **(D)** miR-124 expression levels in IFNγ-MG in monocultures upon the indicated conditions or in coculture with SWE cells modulated for miR-124 after 24 h incubation. Results are mean ± SD from at least three independent experiments. **p < 0.01 and ***p < 0.001 *vs*. naïve MG; ##*p* < 0.01 *vs*.IFNγ-MG cocultured with SWE mock, one-way ANOVA with the Bonferroni *post hoc* test. SWE, human SH-SY5Y cells expressing the APP695 Swedish mutant protein; MG, CHME3 human microglial cells; IFNγ-MG, IFNγ-stimulated CHME3 human microglia cells; co, coculture; siDicer1, siRNA-mediated Dicer-1 silencing.

Once we observed a miR-124 boost in IFNγ-MG when cocultured with the SWE neuronal cells (Figure 3H) and later that miR-124 up/downregulation levels in IFNγ-MG recapitulated its levels in SWE cells modulated with the miR-124 inhibitor or mimic (Figure 5C), we put the hypothesis of a horizontal transfer of miR-124 from SWE cells to IFNγ-MG. To explore such a process, we silenced RNase III endonuclease Dicer1 in IFNγ-MG to block their miRNA production before establishing the coculture, as schematized in Figure 7A. After testing different incubation periods, the 24 h one was the most efficient in siRNA-mediated Dicer-1 silencing (siDicer1), as confirmed by western blot (Figure 7B). In this condition, we also confirmed massive repression (*p* < 0.001) of miR-124 in IFNγ-MG cells to only 3.4% of the unrepressed control levels (Figure 7C). Besides, we confirmed that Dicer1 silencing was maintained even until 72 h after transfection, thus covering the coculture period of 24 h (data not shown).

After establishing the best silencing conditions, we cocultured the SWE cells with Dicer1-silenced IFNγ-MG. Remarkably, miR-124 levels were still increased in siDicer1 microglia upon coculture with the SWE cells, regardless of the type of modulation (mock control: 80.6-fold; inhibitor: 16.7-fold; mimic: 152.4-fold; *p* < 0.001 for all *vs*. naïve microglia) (Figure 7D). Noteworthy, such miR-124 increase in siDicer1 microglia was still observed in cocultures with the SWE cells treated with the miR-124 inhibitor (*p* < 0.001). However, the effect, in this case, was inferior to that caused by the mock or mimic (at least *p* < 0.01). Notably, miR-124 in Dicer1-silenced IFNγ-MG cells cocultured with SWE cells treated with miR-124 mimic was upregulated compared to the mock, as expected (*p* < 0.05). So far, these results confirm the miR-124 paracrine trafficking from neurons to IFNγ-MG.

### 3.8 Transmission of miR-124 Into IFNγ-Microglia Is Mediated by EXOs From the SWE Neurons

**FIGURE 8:**
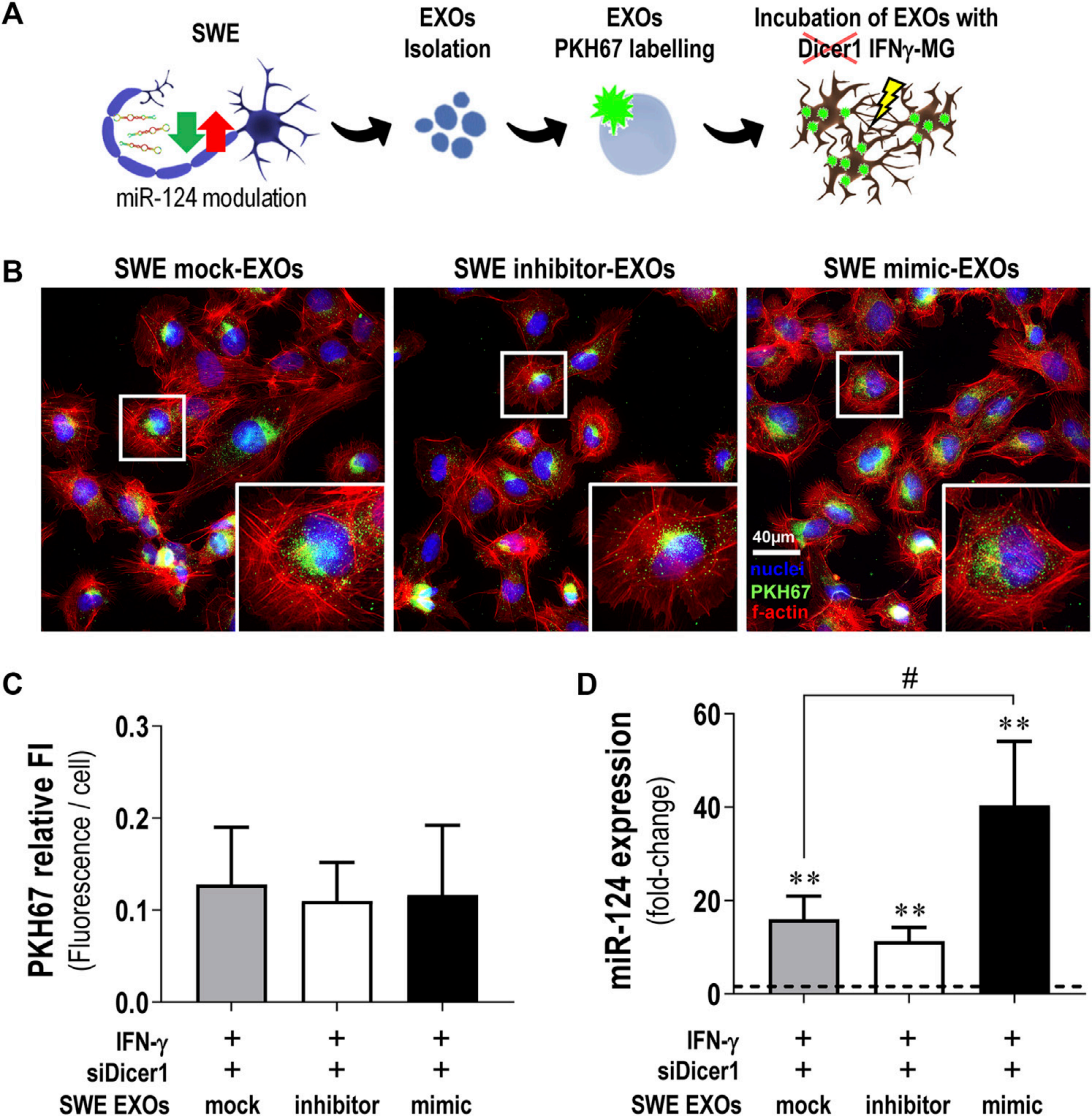
SWE-derived EXOs efficiently deliver miR-124 into Dicer1-silenced IFNγ-MG. After differentiation, SWE cells were transfected with mock or miR124 inhibitor and mimic, while CHME3 microglia were treated with IFNγ followed by Dicer-1 siRNAs, as described before. **(A)** Schematic representation of exosome (EXO) isolation from SWE cells expressing different miR-124 levels (mock, inhibitor, and mimic), labeling with the fluorescent probe PKH767, and incubation with Dicer1-silenced IFNγ-MG. **(B)** Representative fluorescence images of PKH67-labelled EXOs collected by Dicer1-silenced IFNγ-MG (green) and f-actin (red). Nuclei were stained with Hoechst 33258 dye (blue). **(C)** Quantification of PKH67 fluorescence intensity (FI) per microglial cell. **(D)** Expression of miR-124 in Dicer1-silenced IFNγ-MG after incubation with SWE-derived EXOs (mock *vs*. inhibitor *vs*. mimic). Results are mean ± SD from at least three independent experiments. **p < 0.01 *vs*. naïve MG (dashed line), one-way ANOVA with the Bonferroni *post hoc* test; #*p* < 0.01 *vs*. siDicer1 silenced IFNγ-MG cocultured with SWE mock exosomes, two-tailed Student’s *t*-test. SWE, human SH-SY5Y expressing the APP695 Swedish mutant protein; EXOs, exosomes; IFNγ-MG, IFNγ-stimulated CHME3 human microglia cells; siDicer1, siRNA-mediated Dicer-1 silencing.

EXOs have been described to participate in the clearance and dissemination of misfolded proteins in neurodegenerative disorders, including AD (Eitan et al., 2016; Yuyama and Igarashi, 2017). More recently, they were described as specialized miRNA carriers with a high impact on recipient cells’ function (Bell and Taylor, 2016; Brites, 2020). Considering the GO analysis of the cellular component, indicating that miR-124 changes relate to the terms “extracellular exosome” and “secretory vesicle” (Supplementary Table S3), along with our previous background on neuronal miR-124 EXO delivery (Fernandes et al., 2018; Garcia et al., 2021), we investigated whether EXOs were responsible for the miR-124 trafficking from SWE neurons to IFNγ-MG (Figure 8). To do so, we modulated miR-124 levels in SWE cells as before and isolated EXOs from each type of modulation (mock, inhibitor, and mimic) by sequential ultracentrifugation. Then, EXOs were labeled with PKH67 and incubated with Dicer1-silenced IFNγ-MG for 24 h, as schematized in Figure 8A. First, we checked if any miR-124 modulations modified the amount of SWE EXOs internalized by IFNγ-MG (Figures 8B,C). Results clearly show that SWE EXOs, independent of the modulation with the miR-124 inhibitor or mimic, were equally internalized by Dicer1-silenced IFNγ-MG (Figure 8C). Remarkably, after the incubation of the Dicer1-silenced IFNγ-MG with the SWE EXOs, we observed that miR-124 increased in IFNγ-MG (SWE mock-EXO, 16-fold; SWE inhibitor-EXO, 11-fold; SWE mimic-EXO, 40-fold; *p* < 0.01 for all). Such an increase attested the above-described internalization upon coculture with the SWE neuronal cells (Figure 7D). Particularly, SWE mimic-EXOs led to 2.5-fold increased levels of miR-124 in the Dicer1-silenced IFNγ-MG (*p* < 0.05) compared to the effect of EXOs from mock control SWE cells. These results confirm the transfer and delivery of miR-124 from SWE cells into the Dicer1-silenced IFNγ-MG, either in coculture (∼150-fold) or as cargo in EXOs (∼40-fold), supporting its dissemination role.

Interestingly, it seems that the ingested miR-124-enriched EXOs by the Dicer1-silenced IFNγ-MG are mainly metabolized intracellularly, considering that the EXOs released from these cells evidenced less miR-124 (∼25%, *p* < 0.001) in the case of mock, inhibitor, and mimic than those from the naïve microglia (Supplementary Figure S5). Our results indicate that the miR-124-loaded EXOs are internalized by the IFNγ-MG, where they target genes involved in the restoration of microglia’s steady state without further exosomal sorting and propagation.

## 4 Discussion

This study shows that neuronal miR-124 is a major paracrine regulator of microglia neuro-immune function. By modulating miR-124 levels in SWE cells, using a specific inhibitor and mimic, we produced consistent alterations in the inflammatory signature and polarization status of IFNγ-stimulated microglia and its secretome. Inhibition of neuronal miR-124 accentuated the pro-inflammatory signaling triggered by IFNγ to the point of compromising neuronal viability in coculture. Conversely, neuronal miR-124 overexpression switched the microglia pro-inflammatory signature into a pro-regenerative one, while also inhibiting the activity of MMP-2 and MMP-9 in the secretome of SWE + IFNγ-MG. These findings indicate a miR-124-mediated microglia depolarization in an inflammatory milieu, which must probably rescue their neuroprotective functions, compromised in AD (Könnecke and Bechmann, 2013; von Bernhardi et al., 2015; Bachiller et al., 2018). Moreover, we showed that miR-124 is delivered from neuronal cells into activated microglia (IFNγ-MG), with a substantial part of such trafficking being vehiculated by neuronal EXOs.

Although neurons are the main cells responsible for miR-124 expression in the CNS (Åkerblom et al., 2012), the role of this miRNA in AD neuropathology has always been controversial, with opposite perceptions and different levels reported (Sonntag et al., 2012). Due to these inconsistencies in the literature, there is not yet a clear idea about the role of miR-124 in the AD onset and progression, even though its implication is unquestionable (Han et al., 2020). In that matter, we believe that most of the miR-124 contradictions rely on studies performed with different experimental models, disease stages, and brain regions (Brites, 2020). There is no doubt that miR-124 is important in AD. Increasing studies point to the release of miR-124 from neuronal cells as a major signaling messenger for recipient cells (Veremeyko et al., 2019; Garcia et al., 2021). Microglia are one of those recipient cells known to acquire a homeostatic and reparative phenotype upon miR-124 uptake (Ponomarev et al., 2011; Veremeyko et al., 2019). These findings have been conducted to develop multiple therapeutic approaches in different disease contexts (Saraiva et al., 2016; Chivero et al., 2020). Herein, our objective was to examine the *modus operandi* through which miR-124 regulates neuron-microglia crosstalk. Therefore, we established a coculture system including IFNγ-MG and SWE cells, which we previously demonstrated to recapitulate important AD features and overexpress/release elevated miR-124 levels (Fernandes et al., 2018; Garcia et al., 2021).

The decision of choosing IFNγ to stimulate CHME3 microglia was supported by the involvement of this cytokine in AD pathology, reported in both mild- and severe-stage patients compared to individuals with mild cognitive impairment (MCI) (Belkhelfa et al., 2014). Moreover, IFNγ is involved in microglia activation upon Aβ (Meda et al., 1995) and the expression of multiple chemokines, MHC, signal transducing elements, increased ROS and NO generation (Rock et al., 2005; Spencer et al., 2016). While other stimuli, such as bacterial lipopolysaccharide (LPS), simulate infection-associated defense mechanisms and trigger microglia-mediated neurotoxicity (Chien et al., 2016), IFNγ is a sterile stimulus actively involved in AD pathology, with a neuroimmune role over microglia activation and synaptic activity (Ta et al., 2019). Apart from the above-mentioned rationale, IFNγ was also reported as one of the most suitable cytokines to stimulate the CHME3 microglial cells (aliased as HMC3) (Dello Russo et al., 2018). Accordingly, our data depict much of those aspects of microglial IFNγ stimulation, with increased nitrosative activity, and upregulation of pro-inflammatory cytokines and receptors. Upregulation of MHC-II, IL-6, and iNOS upon IFNγ stimulation was expected considering previous studies (Ta et al., 2019; Zhang et al., 2020). However, iNOS activation may not be sufficient to mediate neurotoxicity, considering the relatively benign outcomes of iNOS if not coactivated with NOX (Mander and Brown, 2005). Moreover, despite the predominance of pro-inflammatory markers, IFNγ also induced a modest reparative/anti-inflammatory signature (elevated TGF-β and IL-10), a consequence that has long been discussed and described as the dual-role of IFNγ in inflammation (Mühl and Pfeilschifter, 2003).

Not surprisingly, the coculture led to mutual adaptations in SWE cells and IFNγ-MG. While microglial iNOS and its end-product NO were described as neuronal killers by inhibiting the neuronal respiratory chain (Bal-Price and Brown, 2001), our data indicate that microglial iNOS is hampered upon coculture. Nevertheless, our results suggest that coculture with IFNγ-MG may compromise mitochondria membrane potential in the SWE cells, thus affecting their oxidative capacity. However, the most interesting finding was the marked miR-124 elevation in IFNγ-MG upon coculture with the SWE cells, strongly suggestive of miR-124 microglial uptake, herein confirmed with Dicer1 knock-down experiments in the activated microglial cells.

Major insights on the relevance of miR-124 levels in neuronal-microglial signaling were further revealed after transfection of the SWE cells with the inhibitor and mimic of miR-124. We showed that miR-124 levels secreted by SWE cells (either *via* EXOs or as a soluble factor) recapitulate intracellular ones, pointing to the passive release of miR-124 into EXOs or EXO-free secretome, as reported in our previous study (Garcia et al., 2021), and also observed in other disease-associated miRNAs (Bell and Taylor, 2016). Moreover, we showed that miR-124 overexpression (with mimic) protects neurons from mitochondrial oxidative injury and stress-associated mediators imposed by the coculture with IFNγ-MG. Such a neuroprotective activity was previously explored in a neurodegenerative context, indicating that miR-124 promotes neurite outgrowth under an inflammatory environment associated with activated macrophages (Hartmann et al., 2015).

On the microglia side, Ponomarev et al. were pioneers in showing that peripheral administration of miR-124 in the mouse model of experimental autoimmune encephalomyelitis (EAE) resulted in systemic deactivation of macrophages, microglia quiescence (a disused designation), reduced activation of myelin-specific T cells, and disease suppression, but miR-124 inhibition caused microglia activation (Ponomarev et al., 2011). They further demonstrated the C/EBPα as a direct target of miR-124. Herein, we demonstrate that miR-124 paracrine signaling from the SWE neuronal cells sustained microglia physiology in the IFNγ context and the mimic repressed the expression of C/EBPα. Upregulation of miR-124 in SWE cells with the mimic also repressed the IFNγ-MG overexpression of iNOS and TNF-α, reduced NO release and inhibited the MMP-2 and MMP-9 activities in the secretome from the coculture, while further stimulating the gene expression of ARG-1 and TGF-β, important for repair. Additionally, TGF-β increase is also correlated with the expression of miR-124 (Lu et al., 2019), corroborating its increased gene expression in IFNγ-MG in coculture with the SWE cells treated with the miR-124 mimic.

Herein, we further demonstrate that miR-124 paracrine signaling from SWE neuronal cells sustained microglia physiology in the IFNγ context. By upregulating miR-124 in SWE cells with the mimic, we repressed the IFNγ-MG overexpression of iNOS and TNF-α, reduced NO release, and inhibited the MMP-2 and MMP-9 activities in the secretome from the coculture, while further stimulating the gene expression of ARG-1 and TGF-β, important for repair.

Although iNOS inhibition *per se* may not confirm neuroprotection due to its synergistic dependence on NADPH oxidase (Mander and Brown, 2005), its combination with TNF-α and MMP inhibitions clearly supports a protective role of miR-124 regulation, whenever its expression levels are downregulated. Such neuroprotective potential of miR-124 was confirmed in the proteomic study by the repression of pro-inflammatory and disease-associated mediators. For instance, MAP2K6 and SERPINB1 were downregulated in IFNγ-MG upon the miR-124 mimic influence. For example, MAP2K6 consists of a direct mediator of p38 phosphorylation, which mediates MAPK pathway activation (Zhu et al., 2001), while SERPINB1 regulates innate immune responses and controls the activity of inflammatory caspases (Choi et al., 2019).

Conversely, the inhibition of miR-124 in SWE cells further induced RAGE, HMGB1, iNOS, and IL-1β in the IFNγ-MG when using the coculture system and inhibited IL-10 and ARG-1, thus favoring a predominant reactive phenotype. Such effects were also confirmed by the proteomic analysis of the DEPs that identified: 1) pro-resolving associated proteins as TGFB1; 2) NF-kB inhibitors as PAWR; and 3) disease-associated markers as EFEMP1. All of them were decreased upon miR-124 inhibition. PAWR (aliased as PAR-4) is a tumor suppressor protein linked to NF-κB and BCL2 direct inhibition (Chakraborty et al., 2001; Yang et al., 2016), also indicated as an AD biomarker involved in the regulation of BACE1-mediated APP processing (Xie and Guo, 2005; Greco et al., 2012). In turn, EFEMP1 (known as fibulin-3) was reported to be involved in glial cell migration and neurite outgrowth in the olfactory nerve (Vukovic et al., 2009), while it decreased in the cerebrospinal fluid (CSF) of AD patients (Khoonsari et al., 2016). Intriguingly, PAWR and EFEMP1 were the only two proteins (from the inflammation/immunity cluster) that showed a direct co-regulation by miR-124 levels. Considering their potential in AD, it becomes crucial to deeply explore their involvement in miR-124 expression levels and their mechanisms of action in future studies.

To highlight that the anti-inflammatory and pro-resolving effects of miR-124 in microglia are at least in part mediated by the C/EBPα and the cytokine receptor IL6R (Hatziapostolou et al., 2011; Ponomarev et al., 2011; Brites, 2020), while C/EBPα was reported to regulate the expression of acute-phase response proteins and the histone deacetylase inhibitor, as well as NF-κB and STAT-3 (Ko et al., 2015). However, further mechanistic studies are needed. By repressing IL6R, miR-124 prevents the priming of microglia by IL-6 (Garner et al., 2018), a multifunctional cytokine that when chronically produced, is involved in the pathogenesis of inflammatory disorders (West et al., 2019). In the case of AD, miR-124, besides influencing the microglia activation state, prevents Aβ production, while reducing tau phosphorylation and synaptic loss, thus having potential as a treatment strategy in AD (Zhao et al., 2021).

By interrogating the miRnet.ca database with the 17 DEPs participating in immune regulation, inflammatory response, and response to stimulus, we found that nine of those are direct targets of miR-124, which confirms the relevance of neuronal-derived miR-124 on the microglia function in an inflammatory and pathological milieu. Furthermore, using the full dataset of proteins identified in our proteomic analysis, it was clear that miR-124 was, by far, the miRNA better explaining the proteomic changes in IFNγ-MG (Supplementary Figure S4). In addition, several transcription factors were predicted to be implicated, including C/EBPα, NFATc1, or STAT3, three major miR-124-3p direct targets. In the case of C/EBPα, such a prediction was confirmed in IFNγ-MG by RT-qPCR, showing a marked downregulation upon coculture with mimic-treated SWE cells. Besides miR-124, other miRNAs were also predicted to be engaged, such as miR-1-3p, miR-155-5p, miR-16-5p, miR-125b-5p, or miR-21, and thus of interest in further studies.

Concerning the GO annotation of DEPs, we found an enrichment in neuron-specific terms, such as “synapse,” “dendrite,” and “growth cone” in the cellular component and “response to axon injury,” “regulation of axon extension,” and “axon development” in biological processes (Supplementary Table S3). Because the coculture setup used in our study prevents neuron-microglia direct contact (Phatnani et al., 2013; Wasilewski et al., 2022), we were surprised to find these neuron-specific GO terms. Interestingly, the induction of neuronal gene expression in microglia was previously recognized, though it remains unclear and would be interesting to address (Veremeyko et al., 2019). However, such terms were not found in the GO annotation of the full proteomic dataset (Supplementary Datasheet 3). The comparison of both GO analyses (full dataset *vs*. DEPs) suggests that the enrichment in neuron-specific terms in the DEP dataset is a consequence of the statistical filtering, and despite being significant, such neuron-specific terms do not represent the most predominant signature in microglia full proteome. Nevertheless, we hypothesize that those terms appeared as a consequence of the microglial involvement in synaptic function (Ta et al., 2019) or simply by the paracrine influence of neuronal cells, which are known to profoundly change the microglial gene expression (Baxter et al., 2021). Alternatively, considering that synaptic proteins are released by neuronal EXOs (Fauré et al., 2006), we cannot discard the hypothesis of microglial uptake of such proteins, a phenomenon described in different studies (Paolicelli et al., 2011; Brioschi et al., 2020). It was suggested to be also associated with epigenetic changes occurring in both cell types during development, and soluble neuronal factors may modify microglial phenotypes and functions and be involved in the AD pathology (Veremeyko et al., 2019).

In the AD context, MMP-2 has been reported to participate in the degradation of the blood–brain barrier (BBB), facilitating the infiltration of inflammatory cells (Candelario-Jalil et al., 2011), while MMP-9 was pointed to accentuate the neurobehavioral deficits in a mouse model of AD (Ringland et al., 2021). Although neither MMP-2 nor MMP-9 are described as miR-124 targets, this study provides the first line of evidence showing that miR-124 overexpression reduces their activity in an AD *in vitro* cell model. However, while the inhibition of MMP-2 and MMP-9 activities was indicated to compromise their potential to degrade Aβ toxic species (Hernandez-Guillamon et al., 2015), their elevation may act as inflammatory components and have a proaggregatory influence on tau oligomerization (Wang et al., 2014).

In the last part of this study, we suppressed the new formation of miRNAs in the IFNγ-MG, including miR-124, by silencing the RNase III endonuclease Dicer1, responsible for canonical miRNA processing. Such silencing allowed us to confirm that most of the miR-124 observed in the cocultures of SWE cells + IFNγ-MG derive from neurons. With the GO enrichment in terms of “extracellular exosome” and “secretory vesicle,” we further confirmed that SWE-derived EXOs are major shuttles involved in miR-124 trafficking into microglia. The use of EXOs as miR-124 vehicles has been successfully explored in ischemia (Yang et al., 2017), traumatic brain injury (Yang et al., 2019), and traumatic spinal cord injury (Jiang et al., 2020), as well as in cocaine-mediated microglial activation (Chivero et al., 2020) and Huntington disease (Lee et al., 2017). All these studies converged on the benefits of miR-124, especially by redirecting microglia into a neuroprotective phenotype.

We believe that our study draws attention to the complexity of miRNA-based therapies but overall clarifies their potential as neuropharmacological approaches in the AD field. However, by only using immortalized cell lines, this study must be understood as an opportunity to explore further the potential of miR-124-based strategies in AD, namely, the possibility of developing miRNA-loaded EXOs (de Abreu et al., 2021) and their future prospects as therapeutics (Munir et al., 2020). Although our coculture system with immortalized cell lines is more advanced than monoculture systems, a big gap still exists between each of them and iPSCs-derived models and/or primary human cells, as depicted in Supplementary Figure S3. Hence, it is essential to validate the neuroprotective potential of regulating miR-124 expression levels in more advanced human personalized AD models, as the three-dimensional spheroids and organoids (Pomeshchik et al., 2020; Chen et al., 2021), not forgetting the possibility of new animal models (Baglietto-Vargas et al., 2021; Le Bras, 2021). Future studies should also characterize the entire EXO cargo and use miR-124 cellular probing (fluorescent *in situ* hybridization) to assess its subcellular localization. It is also important to investigate the consequences of Dicer1 silencing on EXO biogenesis and release by microglia because little is known in this regard. Finally, it should be considered that miR-124 has hundreds of direct and indirect targets, as evidenced in our proteomic analysis, which may have off-target effects and benefit as a multitarget approach.

## 5 Conclusion

**FIGURE 9:**
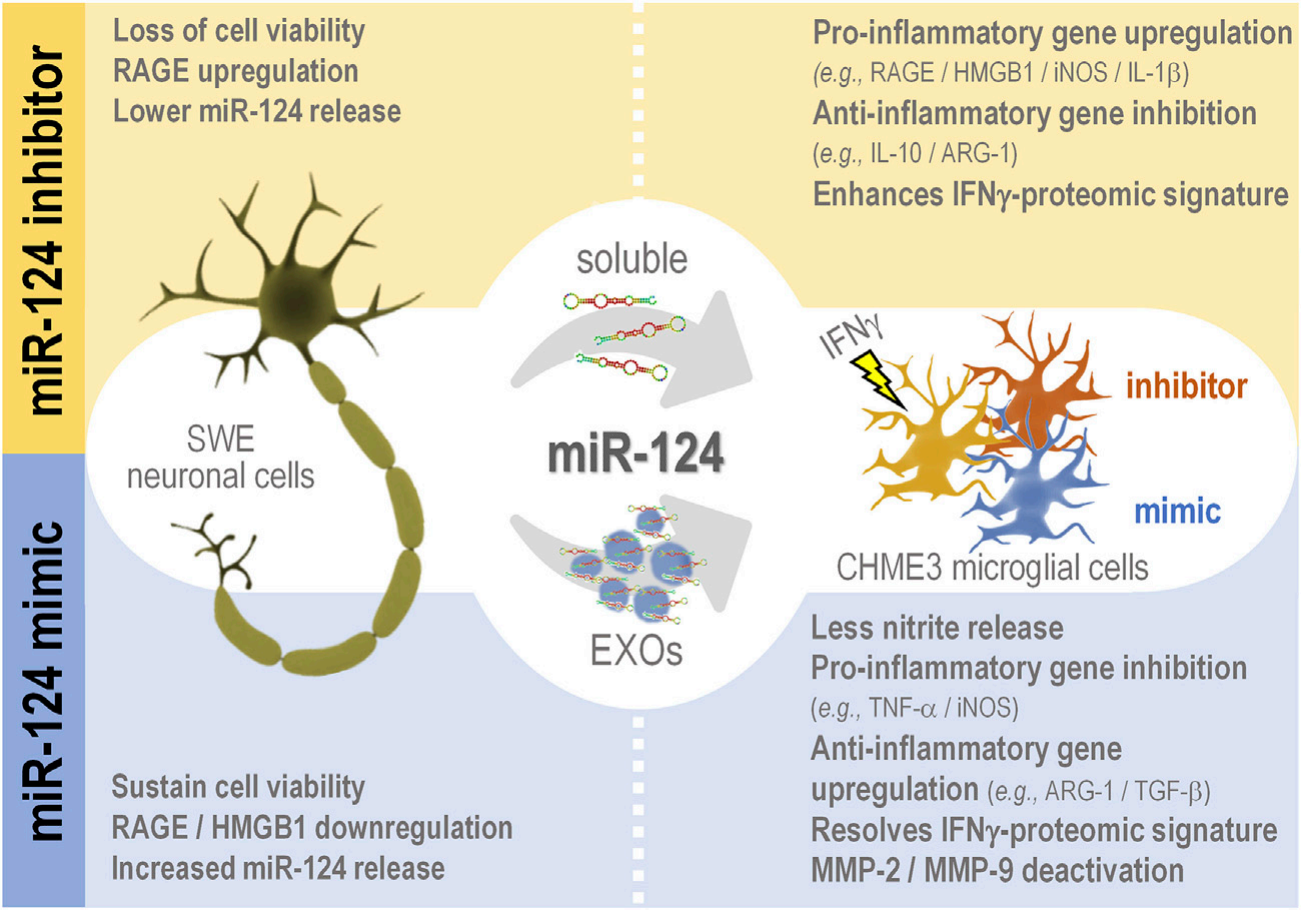
Schematic representation of the miR-124 trafficking between SWE cells and IFNγ-MG and consequences associated with miR-124 inhibition (inhibitor) and upregulation (mimic) in each cell type. miR-124 is transferred from the SWE cells to IFNγ-MG *via* neuronal secretome (either free or in EXOs). The neuronal miR-124 inhibition, apart from reducing its levels in SWE cells and their secretome, also compromises cell viability and induces RAGE upregulation upon coculture with IFNγ-MG. Besides the effects in the SWE cells, the miR-124 inhibition leads to paracrine effects in IFNγ-MG, including pro-inflammatory gene expression and IFN-associated proteomic signature. On the contrary, the miR-124 mimic upregulates its expression/release by SWE cells, while sustaining cell viability in the presence of IFNγ-MG cells, which in turn manifest a homeostatic state profile with less nitrosative activity, predominant anti-inflammatory gene/proteomic signature, and reduced extracellular matrix degradation. Considering the progress in the field of EXOs as miRNA carriers to alleviate pathological cellular states in several diseases, EXOs enriched in miR-124 may open a new avenue in therapeutic interventions in AD. miR, miRNA; RAGE, receptor for advanced glycation end products; HMGB1, high mobility group box protein 1; iNOS, inducible nitric oxide synthase; IL, interleukin; ARG-1, arginase-1; TNF-α, tumor necrosis factor α; TGF-β, transforming growth factor β; MMP, metalloproteinase.

This study provides novel insights into the relevance of miR-124 to sustain nerve cell homeostatic balance in pathological neurodegeneration and associated inflammation. Besides being important to prevent neuronal dysfunction, as shown in advanced AD cell models (Garcia et al., 2021), we established miR-124 as a key player in the paracrine signaling between the SWE neuronal and microglial cells after stimulation with IFNγ (IFNγ-MG), to mimic neuro-immune dysregulation. A schematic summary of the results obtained is depicted in Figure 9. Herein, we reveal that low levels of miR-124, upon modulation with its inhibitor, aggravate the reactivity of IFNγ-MG and compromise their neighboring SWE cell viability, as demonstrated by their coculture. In contrast, the upregulation of miR-124 with its mimic, apart from preserving the SWE cell viability in the presence of IFNγ-MG, contributes to prevent excessive microglia activation and plays an inhibitory role in MMP-2 and MMP-9 activation associated with AD progression. Moreover, besides identifying multiple targets of miR-124 in the activated microglia, our proteomic analysis reveals deep changes in the expression of proteins involved in vesicular trafficking, extracellular matrix, and neuro-immune function, as well as of several proteins involved in synaptic function, dendrite outgrowth, and cytoskeleton remodeling. Of note, some proteins involving a neuro-immune function (e.g., EFEMP1 and PAWR) are reported in AD, thus opening a new field for future research.

Together, our results validate the relevance of miR-124 in the maintenance of neuron-microglia homeostatic balance in AD conditions, namely, in the presence of inflammation and microglia activation. Furthermore, we show that SWE-derived EXOs are substantial players in translating miR-124 into IFNγ-MG cells, reprogramming microglia signature, and influencing microglia local responses in health and disease. As therapeutic miRNA-enriched EXOs have been tested as promising therapies in neurodegenerative diseases, we anticipate that miR-124-loaded EXOs may constitute an advance in future AD treatment strategies.

## Data Availability

The datasets presented in this study can be found in online repositories. The names of the repository/repositories and accession number(s) can be found in the article/Supplementary Material.

## Footnotes

1
https://www.ebi.ac.uk/pride/archive/projects/PXD030315.
