# Supplementary material for: Protective Signature of IFNγ-Stimulated Microglia Relies on miR-124-3p Regulation From the Secretome Released by Mutant APP Swedish Neuronal Cells
Source: Front Pharmacol. 2022 May 10;13:833066. doi: 10.3389/fphar.2022.833066 (PMC9127204; doi:10.3389/fphar.2022.833066)
Supplement: Supplementary file 1 [file DataSheet2.PDF]

PANTHER will be unavailable on February 23rd, 2022 for maintenance starting at 10:00AM PDT for approximately 6 hours

Analysis Summary: Please report in publication

Analysis Type: PANTHER Overrepresentation Test (Released 20220202)

Annotation Version and Release Date: PANTHER version 16.0 Released 2020-12-01

Analyzed List: PANTHER FULL DATASET.txt (Homo sapiens) Change

Reference List: Homo sapiens (all genes in database) Change

Annotation Data Set: PANTHER GO-Slim Biological Process

Test Type: (•) Fisher's Exact ( ) Binomial

Correction: Calculate False Discovery Rate Use the Bonferroni correction for multiple testing No correction

Results

	Reference list	PANTHER FULL DATASET.txt
Uniquely Mapped IDS:	20595 out of 20595	3666 out of 3747
Unmapped IDs:	0	119
Multiple mapping information:	0	205

Bonferroni count: 1810

Export Table XML with user input ids JSON with user input ids View: -- Please select a chart to display --

Displaying only results for Bonferroni-corrected for P < 0.05, click here to display all results

	Homo sapiens (REF)	PANTHER FULL DATASET.txt (Hierarchy NEW!)				
PANTHER GO-Slim Biological Process	#	#	expected	Fold Enrichment	+/-	P value
maturation of SSU-rRNA from tricistronic rRNA transcript (SSU-rRNA, 5.8S rRNA, LSU-rRNA)	28	21	5.09	4.12	+	7.08E-03
↳maturation of SSU-rRNA	37	28	6.73	4.16	+	1.59E-04
↳ribosomal small subunit biogenesis	54	40	9.82	4.07	+	4.63E-07
↳ribosome biogenesis	178	123	32.38	3.80	+	1.53E-23
↳ribonucleoprotein complex biogenesis	235	158	42.76	3.70	+	7.73E-30
↳cellular component biogenesis	1040	380	189.21	2.01	+	1.21E-26
↳cellular component organization or biogenesis	2750	843	500.33	1.68	+	3.42E-40

↳cellular process	9951	2175	1810.46	1.20	+	1.06E-24
↳rRNA processing	117	81	21.29	3.81	+	6.71E-15
↳ncRNA processing	210	112	38.21	2.93	+	1.12E-14
↳ncRNA metabolic process	260	143	47.30	3.02	+	2.90E-20
↳nucleobase-containing compound metabolic process	3150	714	573.10	1.25	+	2.61E-05
↳heterocycle metabolic process	3198	730	581.84	1.25	+	5.78E-06
↳cellular metabolic process	5684	1331	1034.13	1.29	+	6.35E-19
↳metabolic process	6178	1398	1124.01	1.24	+	3.03E-15
↳cellular aromatic compound metabolic process	3214	730	584.75	1.25	+	1.27E-05
↳cellular nitrogen compound metabolic process	3483	851	633.69	1.34	+	1.80E-13
↳nitrogen compound metabolic process	5350	1211	973.36	1.24	+	4.44E-12
↳primary metabolic process	5603	1263	1019.40	1.24	+	1.89E-12
↳organic cyclic compound metabolic process	3262	737	593.48	1.24	+	2.05E-05
↳organic substance metabolic process	5910	1346	1075.25	1.25	+	3.28E-15
↳macromolecule metabolic process	5051	1112	918.97	1.21	+	8.44E-08
↳RNA processing	485	275	88.24	3.12	+	4.45E-43
↳gene expression	3048	687	554.55	1.24	+	1.19E-04
↳rRNA metabolic process	124	87	22.56	3.86	+	2.11E-16
ribosomal large subunit biogenesis	61	45	11.10	4.05	+	3.96E-08
mRNA export from nucleus	32	22	5.82	3.78	+	1.15E-02
↳mRNA-containing ribonucleoprotein complex export from nucleus	32	22	5.82	3.78	+	1.15E-02
↳ribonucleoprotein complex export from nucleus	44	34	8.01	4.25	+	4.68E-06
↳RNA export from nucleus	61	43	11.10	3.87	+	3.06E-07
↳nuclear export	71	48	12.92	3.72	+	7.75E-08
↳nucleocytoplasmic transport	105	69	19.10	3.61	+	1.31E-11
↳nuclear transport	105	69	19.10	3.61	+	1.31E-11
↳intracellular transport	759	286	138.09	2.07	+	7.38E-21
↳transport	1927	449	350.59	1.28	+	2.19E-03
↳establishment of localization	1961	460	356.78	1.29	+	7.95E-04
↳localization	2410	546	438.47	1.25	+	2.30E-03
↳cellular localization	1108	371	201.59	1.84	+	3.31E-20
↳establishment of localization in cell	892	311	162.29	1.92	+	1.33E-18
↳RNA transport	64	44	11.64	3.78	+	3.35E-07
↳nucleic acid transport	64	44	11.64	3.78	+	3.35E-07
↳nucleobase-containing compound transport	73	44	13.28	3.31	+	6.73E-06

↳organic substance transport	887	260	161.38	1.61	+	5.18E-08
↳nitrogen compound transport	758	245	137.91	1.78	+	4.96E-11
↳establishment of RNA localization	64	44	11.64	3.78	+	3.35E-07
↳RNA localization	69	46	12.55	3.66	+	2.73E-07
↳macromolecule localization	943	301	171.57	1.75	+	1.51E-13
↳protein-containing complex localization	210	71	38.21	1.86	+	3.35E-02
↳protein localization	829	286	150.83	1.90	+	2.44E-16
↳ribonucleoprotein complex localization	44	34	8.01	4.25	+	4.68E-06
↳protein export from nucleus	54	40	9.82	4.07	+	4.63E-07
↳intracellular protein transport	536	204	97.52	2.09	+	1.41E-14
↳protein transport	625	231	113.71	2.03	+	1.72E-15
↳establishment of protein localization	637	234	115.89	2.02	+	1.47E-15
↳peptide transport	630	232	114.62	2.02	+	1.57E-15
↳amide transport	644	233	117.17	1.99	+	7.36E-15
↳cellular protein localization	738	262	134.27	1.95	+	4.00E-16
↳cellular macromolecule localization	740	263	134.63	1.95	+	2.98E-16
↳mRNA transport	33	23	6.00	3.83	+	6.02E-03
alternative mRNA splicing, via spliceosome	49	33	8.91	3.70	+	8.68E-05
↳mRNA splicing, via spliceosome	180	116	32.75	3.54	+	2.96E-20
↳RNA splicing, via transesterification reactions with bulged adenosine as nucleophile	180	116	32.75	3.54	+	2.96E-20
↳RNA splicing, via transesterification reactions	180	116	32.75	3.54	+	2.96E-20
↳RNA splicing	220	135	40.03	3.37	+	2.28E-22
↳mRNA processing	235	145	42.76	3.39	+	2.42E-24
↳mRNA metabolic process	315	177	57.31	3.09	+	1.86E-26
cytoplasmic translation	54	36	9.82	3.66	+	2.62E-05
↳translation	236	132	42.94	3.07	+	4.87E-19
↳peptide biosynthetic process	239	134	43.48	3.08	+	2.03E-19
↳peptide metabolic process	307	150	55.85	2.69	+	1.40E-17
↳cellular amide metabolic process	407	179	74.05	2.42	+	1.55E-17
↳organonitrogen compound metabolic process	2699	685	491.05	1.39	+	6.15E-13
↳organonitrogen compound biosynthetic process	657	230	119.53	1.92	+	3.10E-13
↳amide biosynthetic process	276	143	50.21	2.85	+	1.63E-18
↳cellular protein metabolic process	1906	514	346.77	1.48	+	1.26E-12
↳protein metabolic process	2236	542	406.81	1.33	+	9.52E-07
regulation of alternative mRNA splicing, via spliceosome	45	30	8.19	3.66	+	4.09E-04

↳regulation of mRNA splicing, via spliceosome	65	43	11.83	3.64	+	1.28E-06
↳regulation of mRNA processing	75	50	13.65	3.66	+	4.42E-08
↳regulation of metabolic process	3093	463	562.73	.82	-	3.07E-02
↳regulation of biological process	5324	764	968.63	.79	-	1.04E-09
↳biological regulation	5797	860	1054.69	.82	-	5.66E-08
↳regulation of mRNA metabolic process	105	57	19.10	2.98	+	8.15E-07
↳regulation of RNA metabolic process	2113	276	384.43	.72	-	3.42E-05
↳regulation of nucleobase-containing compound metabolic process	2161	287	393.17	.73	-	9.56E-05
↳regulation of cellular metabolic process	2835	405	515.79	.79	-	1.05E-03
↳regulation of cellular process	5092	711	926.43	.77	-	1.76E-11
↳regulation of nitrogen compound metabolic process	2715	387	493.96	.78	-	1.69E-03
↳regulation of primary metabolic process	2743	388	499.05	.78	-	7.12E-04
↳regulation of RNA splicing	88	55	16.01	3.44	+	2.86E-08
protein import into nucleus	52	33	9.46	3.49	+	2.41E-04
↳protein localization to nucleus	57	36	10.37	3.47	+	7.20E-05
↳protein localization to organelle	358	126	65.13	1.93	+	1.87E-06
↳import into nucleus	52	33	9.46	3.49	+	2.41E-04
↳establishment of protein localization to organelle	171	87	31.11	2.80	+	3.85E-10
↳protein import	85	46	15.46	2.97	+	5.00E-05
protein depolymerization	40	24	7.28	3.30	+	2.40E-02
↳cellular protein complex disassembly	50	31	9.10	3.41	+	8.30E-04
↳protein-containing complex disassembly	71	39	12.92	3.02	+	3.37E-04
↳protein-containing complex subunit organization	516	224	93.88	2.39	+	7.06E-22
↳cellular component organization	2624	751	477.40	1.57	+	8.27E-27
↳cellular component disassembly	116	54	21.10	2.56	+	1.50E-04
ribonucleoprotein complex assembly	104	60	18.92	3.17	+	4.26E-08
↳ribonucleoprotein complex subunit organization	107	62	19.47	3.18	+	1.74E-08
↳cellular protein-containing complex assembly	407	169	74.05	2.28	+	1.85E-14
↳protein-containing complex assembly	433	182	78.78	2.31	+	4.07E-16
↳cellular component assembly	909	284	165.38	1.72	+	1.24E-11
protein folding	87	50	15.83	3.16	+	2.20E-06
translational elongation	236	132	42.94	3.07	+	4.87E-19
endoplasmic reticulum to Golgi vesicle-mediated transport	88	45	16.01	2.81	+	3.05E-04
↳Golgi vesicle transport	281	104	51.12	2.03	+	6.39E-06
↳vesicle-mediated transport	803	227	146.10	1.55	+	1.53E-05

mitotic sister chromatid segregation	61	31	11.10	2.79	+	2.52E-02
↳chromosome organization	418	137	76.05	1.80	+	1.55E-05
↳organelle organization	1979	603	360.05	1.67	+	3.59E-26
↳mitotic nuclear division	267	86	48.58	1.77	+	1.81E-02
↳mitotic cell cycle process	267	86	48.58	1.77	+	1.81E-02
↳mitotic cell cycle	267	86	48.58	1.77	+	1.81E-02
↳organelle fission	340	102	61.86	1.65	+	3.38E-02
RNA catabolic process	104	51	18.92	2.70	+	1.15E-04
↳nucleobase-containing compound catabolic process	142	59	25.84	2.28	+	1.03E-03
↳organic cyclic compound catabolic process	183	69	33.29	2.07	+	1.81E-03
↳organic substance catabolic process	753	246	137.00	1.80	+	1.54E-11
↳catabolic process	921	278	167.56	1.66	+	6.75E-10
↳cellular nitrogen compound catabolic process	163	66	29.66	2.23	+	4.72E-04
↳cellular catabolic process	816	254	148.46	1.71	+	5.82E-10
↳aromatic compound catabolic process	167	63	30.38	2.07	+	5.84E-03
↳heterocycle catabolic process	165	66	30.02	2.20	+	5.54E-04
↳cellular macromolecule catabolic process	441	163	80.23	2.03	+	2.46E-10
↳macromolecule catabolic process	479	169	87.15	1.94	+	2.52E-09
actin polymerization or depolymerization	84	41	15.28	2.68	+	2.11E-03
↳actin filament organization	184	76	33.48	2.27	+	2.13E-05
↳actin cytoskeleton organization	268	105	48.76	2.15	+	4.88E-07
↳actin filament-based process	280	105	50.94	2.06	+	3.81E-06
↳cytoskeleton organization	649	211	118.08	1.79	+	2.58E-09
↳supramolecular fiber organization	274	110	49.85	2.21	+	4.93E-08
regulation of actin filament polymerization	72	35	13.10	2.67	+	1.73E-02
↳regulation of actin polymerization or depolymerization	74	37	13.46	2.75	+	3.98E-03
↳regulation of actin filament organization	82	38	14.92	2.55	+	1.74E-02
↳regulation of actin cytoskeleton organization	105	44	19.10	2.30	+	1.96E-02
↳regulation of cytoskeleton organization	151	64	27.47	2.33	+	1.81E-04
↳regulation of organelle organization	275	103	50.03	2.06	+	4.53E-06
↳regulation of cellular component organization	410	143	74.59	1.92	+	2.20E-07
↳regulation of actin filament-based process	106	44	19.29	2.28	+	2.24E-02
↳regulation of supramolecular fiber organization	94	44	17.10	2.57	+	2.15E-03
↳regulation of actin filament length	74	37	13.46	2.75	+	3.98E-03
↳regulation of protein polymerization	79	37	14.37	2.57	+	1.34E-02

↳regulation of protein-containing complex assembly	96	48	17.47	2.75	+	1.41E-04
↳regulation of cellular component biogenesis	161	64	29.29	2.18	+	1.04E-03
protein polymerization	105	49	19.10	2.56	+	7.67E-04
tRNA metabolic process	108	50	19.65	2.54	+	5.88E-04
generation of precursor metabolites and energy	126	56	22.92	2.44	+	2.67E-04
cellular amino acid metabolic process	137	54	24.93	2.17	+	1.22E-02
↳carboxylic acid metabolic process	338	120	61.49	1.95	+	3.29E-06
↳oxoacid metabolic process	357	122	64.95	1.88	+	1.64E-05
↳organic acid metabolic process	375	123	68.23	1.80	+	9.59E-05
↳small molecule metabolic process	654	215	118.99	1.81	+	5.66E-10
nucleotide biosynthetic process	117	46	21.29	2.16	+	4.86E-02
↳nucleotide metabolic process	196	81	35.66	2.27	+	7.48E-06
↳nucleoside phosphate metabolic process	202	83	36.75	2.26	+	7.17E-06
↳organophosphate metabolic process	388	120	70.59	1.70	+	1.93E-03
↳nucleobase-containing small molecule metabolic process	235	95	42.76	2.22	+	8.07E-07
↳nucleoside phosphate biosynthetic process	119	47	21.65	2.17	+	3.55E-02
↳nucleobase-containing compound biosynthetic process	2297	315	417.91	.75	-	5.73E-04
↳heterocycle biosynthetic process	2322	324	422.46	.77	-	2.18E-03
↳aromatic compound biosynthetic process	2323	324	422.64	.77	-	2.18E-03
↳organic cyclic compound biosynthetic process	2350	328	427.55	.77	-	1.86E-03
purine nucleotide metabolic process	166	65	30.20	2.15	+	1.46E-03
↳purine-containing compound metabolic process	178	66	32.38	2.04	+	4.84E-03
ribonucleotide metabolic process	155	58	28.20	2.06	+	1.59E-02
↳ribose phosphate metabolic process	163	64	29.66	2.16	+	1.28E-03
proteasome-mediated ubiquitin-dependent protein catabolic process	194	69	35.30	1.95	+	1.12E-02
↳ubiquitin-dependent protein catabolic process	283	94	51.49	1.83	+	2.17E-03
↳modification-dependent protein catabolic process	292	98	53.13	1.84	+	1.05E-03
↳proteolysis involved in cellular protein catabolic process	322	109	58.58	1.86	+	1.44E-04
↳cellular protein catabolic process	323	109	58.77	1.85	+	1.53E-04
↳protein catabolic process	352	112	64.04	1.75	+	1.22E-03
↳organonitrogen compound catabolic process	480	147	87.33	1.68	+	1.54E-04
↳proteolysis	678	187	123.35	1.52	+	1.30E-03
↳modification-dependent macromolecule catabolic process	297	100	54.04	1.85	+	6.33E-04
↳proteasomal protein catabolic process	206	74	37.48	1.97	+	2.95E-03
oxidation-reduction process	228	77	41.48	1.86	+	1.20E-02

organelle localization	282	94	51.31	1.83	+	2.05E-03
cellular response to stress	500	153	90.97	1.68	+	8.19E-05
↳response to stimulus	3027	402	550.72	.73	-	7.61E-08
↳cellular response to stimulus	2522	354	458.85	.77	-	1.12E-03
membrane organization	587	160	106.80	1.50	+	1.61E-02
Unclassified	9793	1478	1781.71	.83	-	0.00E00
regulation of cell communication	813	93	147.92	.63	-	1.13E-02
regulation of signaling	813	93	147.92	.63	-	1.13E-02
ion transmembrane transport	446	42	81.14	.52	-	1.54E-02
↳ion transport	660	55	120.08	.46	-	7.60E-07
cell-cell signaling	425	39	77.32	.50	-	1.61E-02
↳signaling	2218	242	403.54	.60	-	4.97E-14
↳cell communication	2230	247	405.72	.61	-	2.74E-13
transcription by RNA polymerase II	1635	149	297.47	.50	-	9.83E-17
↳transcription, DNA-templated	2047	225	372.43	.60	-	2.93E-12
↳nucleic acid-templated transcription	2047	225	372.43	.60	-	2.93E-12
↳RNA biosynthetic process	2054	232	373.70	.62	-	4.08E-11
regulation of transcription by RNA polymerase II	1591	142	289.46	.49	-	4.30E-17
↳regulation of transcription, DNA-templated	1984	211	360.96	.58	-	2.22E-13
↳regulation of cellular macromolecule biosynthetic process	2112	248	384.25	.65	-	1.14E-09
↳regulation of macromolecule biosynthetic process	2120	248	385.71	.64	-	6.37E-10
↳regulation of biosynthetic process	2141	251	389.53	.64	-	6.45E-10
↳regulation of cellular biosynthetic process	2134	251	388.25	.65	-	9.44E-10
↳regulation of nucleic acid-templated transcription	1984	211	360.96	.58	-	2.22E-13
↳regulation of RNA biosynthetic process	1984	211	360.96	.58	-	2.22E-13
animal organ development	385	33	70.05	.47	-	1.00E-02
↳developmental process	1271	162	231.24	.70	-	1.04E-02
↳multicellular organismal process	1385	138	251.98	.55	-	1.33E-10
innate immune response	232	16	42.21	.38	-	4.83E-02
↳response to other organism	364	29	66.23	.44	-	3.55E-03
↳response to external biotic stimulus	364	29	66.23	.44	-	3.55E-03
↳response to external stimulus	611	56	111.16	.50	-	1.12E-04
↳response to biotic stimulus	365	29	66.41	.44	-	3.60E-03
↳interspecies interaction between organisms	376	33	68.41	.48	-	2.14E-02
↳defense response	395	32	71.87	.45	-	1.70E-03
↳immune response	526	25	95.70	.26	-	1.19E-12

↳immune system process	676	41	122.99	.33	-	1.11E-12
metal ion transport	320	18	58.22	.31	-	2.22E-05
↳cation transport	444	35	80.78	.43	-	1.63E-04
lymphocyte activation	176	6	32.02	.19	-	4.40E-04
↳leukocyte activation	189	9	34.39	.26	-	4.26E-03
↳cell activation	198	12	36.02	.33	-	3.58E-02
positive regulation of lymphocyte activation	125	4	22.74	.18	-	2.19E-02
↳positive regulation of leukocyte activation	130	5	23.65	.21	-	3.88E-02
↳regulation of leukocyte activation	153	5	27.84	.18	-	2.12E-03
↳regulation of cell activation	154	5	28.02	.18	-	1.44E-03
↳regulation of immune system process	297	15	54.04	.28	-	9.06E-06
↳positive regulation of immune system process	234	12	42.57	.28	-	5.32E-04
↳positive regulation of cell activation	130	5	23.65	.21	-	3.88E-02
↳regulation of lymphocyte activation	144	4	26.20	.15	-	1.58E-03
inflammatory response	128	4	23.29	.17	-	1.53E-02
defense response to bacterium	150	4	27.29	.15	-	4.88E-04
↳response to bacterium	213	5	38.75	.13	-	3.26E-07
cell recognition	125	3	22.74	.13	-	3.32E-03
activation of adenylate cyclase activity	102	2	18.56	.11	-	1.51E-02
adenylate cyclase-activating G protein-coupled receptor signaling pathway	102	2	18.56	.11	-	1.51E-02
↳second-messenger-mediated signaling	239	14	43.48	.32	-	3.04E-03
↳signal transduction	2062	223	375.15	.59	-	3.57E-13
↳G protein-coupled receptor signaling pathway, coupled to cyclic nucleotide second messenger	164	8	29.84	.27	-	2.98E-02
↳G protein-coupled receptor signaling pathway	448	16	81.51	.20	-	1.17E-13
adaptive immune response based on somatic recombination of immune receptors built from immunoglobulin superfamily domains	120	2	21.83	.09	-	1.47E-03
↳adaptive immune response	142	3	25.84	.12	-	3.23E-04
regulation of membrane potential	127	2	23.11	.09	-	4.17E-04
sensory perception of chemical stimulus	89	1	16.19	.06	-	2.02E-02
↳sensory perception	129	1	23.47	.04	-	2.43E-05
↳nervous system process	220	3	40.03	.07	-	1.86E-09
↳system process	302	14	54.95	.25	-	1.50E-06
B cell receptor signaling pathway	112	1	20.38	.05	-	4.69E-04
↳antigen receptor-mediated signaling pathway	142	4	25.84	.15	-	1.53E-03
↳immune response-activating cell surface receptor signaling pathway	154	5	28.02	.18	-	1.44E-03
↳immune response-activating signal transduction	154	5	28.02	.18	-	1.44E-03

↳activation of immune response	165	7	30.02	.23	-	6.50E-03
↳positive regulation of immune response	191	9	34.75	.26	-	3.00E-03
↳positive regulation of response to stimulus	504	49	91.70	.53	-	9.76E-03
↳regulation of response to stimulus	1004	101	182.67	.55	-	9.25E-07
↳regulation of immune response	205	9	37.30	.24	-	5.37E-04
↳immune response-regulating signaling pathway	158	5	28.75	.17	-	9.97E-04
↳immune response-regulating cell surface receptor signaling pathway	158	5	28.75	.17	-	9.97E-04
↳cell surface receptor signaling pathway	1003	84	182.48	.46	-	2.25E-11
lymphocyte mediated immunity	120	1	21.83	.05	-	1.36E-04
↳leukocyte mediated immunity	127	1	23.11	.04	-	3.73E-05

About | Release Information | Contact Us | System Requirements | Privacy Policy | Disclaimer
© Copyright 2022 Paul Thomas All Rights Reserved.

PANTHER will be unavailable on February 23rd, 2022 for maintenance starting at 10:00AM PDT for approximately 6 hours

Analysis Summary: Please report in publication

Analysis Type: PANTHER Overrepresentation Test (Released 20220202)

Annotation Version and Release Date: PANTHER version 16.0 Released 2020-12-01

**Analyzed List:**	PANTHER FULL DATASET.txt (Homo sapiens)	Change
**Reference List:**	Homo sapiens (all genes in database)	Change

Annotation Data Set: PANTHER GO-Slim Cellular Component

Test Type: (●) Fisher's Exact ( ) Binomial

Correction: Calculate False Discovery Rate Use the Bonferroni correction for multiple testing No correction

Results

	Reference list	PANTHER FULL DATASET.txt
Uniquely Mapped IDS:	20595 out of 20595	3666 out of 3747
Unmapped IDs:	0	119
Multiple mapping information:	0	205

Bonferroni count: 438

Export Table XML with user input ids JSON with user input ids View: -- Please select a chart to display --

Displaying only results for Bonferroni-corrected for P < 0.05, click here to display all results

	Homo sapiens (REF)	PANTHER FULL DATASET.txt (Hierarchy NEW! ?)				
PANTHER GO-Slim Cellular Component	#	#	expected	Fold Enrichment	+/-	P value
proteasome regulatory particle	16	16	2.91	5.50	+	2.13E-03
↳proteasome accessory complex	16	16	2.91	5.50	+	2.13E-03
↳proteasome complex	38	32	6.91	4.63	+	7.72E-07
↳endopeptidase complex	42	33	7.64	4.32	+	1.45E-06
↳peptidase complex	60	39	10.92	3.57	+	2.69E-06
↳catalytic complex	858	285	156.10	1.83	+	3.71E-15
↳protein-containing complex	2876	985	523.25	1.88	+	2.91E-69
↳intracellular	8253	2254	1501.53	1.50	+	1.42E-111
small-subunit processome	34	29	6.19	4.69	+	3.63E-06
↳preribosome	71	58	12.92	4.49	+	5.70E-13
↳ribonucleoprotein complex	413	270	75.14	3.59	+	9.84E-52
preribosome, large subunit precursor	20	17	3.64	4.67	+	4.60E-03
t-UTP complex	47	38	8.55	4.44	+	5.38E-08
↳nucleolus	176	105	32.02	3.28	+	6.00E-17
↳intracellular non-membrane-bounded organelle	1959	548	356.42	1.54	+	5.30E-17
↳non-membrane-bounded organelle	1959	548	356.42	1.54	+	5.30E-17
↳organelle	6781	1766	1233.72	1.43	+	2.54E-58

↳cellular anatomical entity	11122	2420	2023.51	1.20	+	6.22E-31
↳intracellular organelle	6633	1752	1206.79	1.45	+	1.23E-61
↳nuclear lumen	1398	330	254.35	1.30	+	7.67E-03
↳nucleus	3956	953	719.74	1.32	+	6.06E-15
↳intracellular membrane-bounded organelle	5903	1499	1073.98	1.40	+	1.71E-39
↳membrane-bounded organelle	5999	1514	1091.44	1.39	+	8.46E-39
↳intracellular organelle lumen	1494	368	271.81	1.35	+	6.43E-05
↳organelle lumen	1494	368	271.81	1.35	+	6.43E-05
↳membrane-enclosed lumen	1494	368	271.81	1.35	+	6.43E-05
cytosolic large ribosomal subunit	52	41	9.46	4.33	+	1.74E-08
↳cytosolic ribosome	91	71	16.56	4.29	+	2.15E-15
↳cytosol	802	348	145.91	2.38	+	2.93E-36
↳cytoplasm	4722	1475	859.11	1.72	+	2.96E-90
↳ribosome	138	97	25.11	3.86	+	3.72E-19
↳large ribosomal subunit	76	53	13.83	3.83	+	7.86E-10
↳ribosomal subunit	127	91	23.11	3.94	+	2.71E-18
90S preribosome	31	24	5.64	4.26	+	2.37E-04
cytosolic small ribosomal subunit	38	29	6.91	4.19	+	2.00E-05
↳small ribosomal subunit	51	38	9.28	4.10	+	2.77E-07
U2-type spliceosomal complex	35	24	6.37	3.77	+	1.10E-03
↳spliceosomal complex	124	75	22.56	3.32	+	6.71E-12
U2 snRNP	33	22	6.00	3.66	+	3.95E-03
↳spliceosomal snRNP complex	82	53	14.92	3.55	+	6.41E-09

↳small nuclear ribonucleoprotein complex	83	53	15.10	3.51	+	8.94E-09
↳Sm-like protein family complex	91	55	16.56	3.32	+	2.80E-08
vesicle coat	40	26	7.28	3.57	+	8.78E-04
↳membrane coat	55	35	10.01	3.50	+	2.33E-05
↳membrane protein complex	615	163	111.89	1.46	+	1.16E-02
↳coated membrane	55	35	10.01	3.50	+	2.33E-05
↳coated vesicle membrane	46	30	8.37	3.58	+	1.39E-04
↳cytoplasmic vesicle	520	151	94.61	1.60	+	4.06E-04
↳intracellular vesicle	522	152	94.97	1.60	+	3.24E-04
↳vesicle	602	166	109.53	1.52	+	1.52E-03
↳organelle membrane	817	230	148.64	1.55	+	3.76E-06
↳coated vesicle	93	53	16.92	3.13	+	2.06E-07
↳whole membrane	336	107	61.13	1.75	+	6.28E-04
↳bounding membrane of organelle	430	128	78.23	1.64	+	1.06E-03
catalytic step 2 spliceosome	48	31	8.73	3.55	+	1.04E-04
Prp19 complex	50	32	9.10	3.52	+	7.80E-05
MCM core complex	30	19	5.46	3.48	+	2.65E-02
MCM complex	30	19	5.46	3.48	+	2.65E-02
U5 snRNP	67	42	12.19	3.45	+	1.57E-06
Golgi-associated vesicle membrane	37	23	6.73	3.42	+	5.75E-03
↳Golgi-associated vesicle	70	39	12.74	3.06	+	6.36E-05
nuclear pore	50	29	9.10	3.19	+	2.05E-03
↳nuclear envelope	110	44	20.01	2.20	+	1.17E-02

↳organelle envelope	330	112	60.04	1.87	+	2.33E-05
↳envelope	330	112	60.04	1.87	+	2.33E-05
↳endomembrane system	1277	366	232.33	1.58	+	9.27E-12
SWI/SNF superfamily-type complex	40	22	7.28	3.02	+	4.86E-02
↳ATPase complex	51	28	9.28	3.02	+	4.84E-03
↳nuclear chromatin	704	65	128.08	.51	-	3.48E-06
↳nuclear chromosome	787	93	143.18	.65	-	1.20E-02
↳chromatin	738	78	134.27	.58	-	4.10E-04
COPII-coated ER to Golgi transport vesicle	44	24	8.01	3.00	+	2.36E-02
mitochondrial matrix	68	31	12.37	2.51	+	2.80E-02
↳mitochondrion	570	187	103.70	1.80	+	6.12E-09
mitochondrial protein complex	136	54	24.74	2.18	+	1.80E-03
actin cytoskeleton	192	72	34.93	2.06	+	3.17E-04
↳cytoskeleton	667	195	121.35	1.61	+	5.36E-06
nucleoplasm	504	167	91.70	1.82	+	4.66E-08
endosome	237	74	43.12	1.72	+	4.21E-02
polymeric cytoskeletal fiber	304	92	55.31	1.66	+	1.83E-02
↳supramolecular fiber	356	102	64.77	1.57	+	3.63E-02
↳supramolecular complex	477	142	86.78	1.64	+	2.62E-04
vacuole	1374	389	249.98	1.56	+	5.68E-12
Unclassified	9302	1269	1692.38	.75	-	0.00E00
leaflet of membrane bilayer	292	24	53.13	.45	-	1.74E-02
cilium	203	14	36.93	.38	-	3.98E-02

extracellular space	1051	59	191.22	.31	-	6.35E-24
↳extracellular region	1203	75	218.87	.34	-	2.24E-24
integral component of plasma membrane	786	36	143.00	.25	-	1.36E-21
↳intrinsic component of plasma membrane	798	36	145.19	.25	-	3.37E-22
↳intrinsic component of membrane	1180	97	214.69	.45	-	5.29E-15
↳integral component of membrane	1151	93	209.41	.44	-	4.17E-15
external side of plasma membrane	217	6	39.48	.15	-	2.58E-07
↳cell surface	325	16	59.13	.27	-	2.30E-07
↳side of membrane	292	24	53.13	.45	-	1.74E-02
cation channel complex	121	3	22.01	.14	-	1.79E-03
↳ion channel complex	131	6	23.83	.25	-	3.16E-02

About | Release Information | Contact Us | System Requirements | Privacy Policy | Disclaimer
© Copyright 2022 Paul Thomas All Rights Reserved.

LOGIN REGISTER CONTACT US

Home About PANTHER Data PANTHER Tools PANTHER Services Workspace Downloads Help/Tutorial

PANTHER will be unavailable on February 23rd, 2022 for maintenance starting at 10:00AM PDT for approximately 6 hours

Analysis Summary: Please report in publication ?

Analysis Type: PANTHER Overrepresentation Test (Released 20220202)

Annotation Version and Release Date: PANTHER version 16.0 Released 2020-12-01

Analyzed List: PANTHER FULL DATASET.txt (Homo sapiens) Change

Reference List: Homo sapiens (all genes in database) Change

Annotation Data Set: PANTHER GO-Slim Molecular Function ?

Test Type: (•) Fisher's Exact ( ) Binomial

Correction: ( ) Calculate False Discovery Rate (•) Use the Bonferroni correction for multiple testing ? ( ) No correction

Results ?

	Reference list	PANTHER FULL DATASET.txt
Uniquely Mapped IDS:	20595 out of 20595	3666 out of 3747
Unmapped IDs:	0	119
Multiple mapping information:	0	205

Bonferroni count: 510

Export Table XML with user input ids JSON with user input ids View: -- Please select a chart to display --

Displaying only results for Bonferroni-corrected for P < 0.05, click here to display all results

	Homo sapiens (REF)	PANTHER FULL DATASET.txt (Hierarchy NEW! ?)				
PANTHER GO-Slim Molecular Function	#	#	expected	Fold Enrichment	+/-	P value

translation initiation factor activity	32	23	5.82	3.95	+	1.17E-03
↳translation regulator activity	71	40	12.92	3.10	+	4.20E-05
↳RNA binding	599	325	108.98	2.98	+	5.57E-49
↳binding	5963	1253	1084.89	1.15	+	2.33E-05
structural constituent of ribosome	112	79	20.38	3.88	+	2.26E-15
↳structural molecule activity	242	109	44.03	2.48	+	7.02E-11
rRNA binding	26	18	4.73	3.81	+	2.07E-02
ribonucleoprotein complex binding	39	24	7.10	3.38	+	4.95E-03
↳protein-containing complex binding	332	130	60.40	2.15	+	8.38E-10
helicase activity	54	30	9.82	3.05	+	2.31E-03
↳ATPase activity	306	124	55.67	2.23	+	5.01E-10
↳nucleoside-triphosphatase activity	592	227	107.71	2.11	+	3.01E-17
↳pyrophosphatase activity	624	237	113.53	2.09	+	9.88E-18
↳hydrolase activity, acting on acid anhydrides, in phosphorus-containing anhydrides	626	237	113.89	2.08	+	1.17E-17
↳hydrolase activity, acting on acid anhydrides	626	237	113.89	2.08	+	1.17E-17
↳hydrolase activity	1760	482	320.21	1.51	+	4.32E-13
↳catalytic activity	3989	1056	725.75	1.46	+	8.20E-30
DNA-dependent ATPase activity	49	27	8.91	3.03	+	7.96E-03
ligase activity	119	63	21.65	2.91	+	7.13E-08
unfolded protein binding	55	29	10.01	2.90	+	6.31E-03
↳protein binding	2640	588	480.31	1.22	+	1.57E-03
mRNA binding	174	88	31.66	2.78	+	9.51E-11
isomerase activity	75	35	13.65	2.56	+	7.29E-03
actin filament binding	119	55	21.65	2.54	+	3.55E-05
↳actin binding	170	73	30.93	2.36	+	4.70E-06
↳cytoskeletal protein binding	377	145	68.59	2.11	+	1.71E-10
catalytic activity, acting on a tRNA	82	37	14.92	2.48	+	8.50E-03
↳catalytic activity, acting on RNA	215	96	39.12	2.45	+	3.09E-09
microtubule binding	139	54	25.29	2.14	+	3.92E-03
↳tubulin binding	170	62	30.93	2.00	+	4.52E-03

molecular adaptor activity	119	45	21.65	2.08	+	3.78E-02
GTPase activity	285	103	51.85	1.99	+	7.27E-06
purine nucleotide binding	210	71	38.21	1.86	+	9.45E-03
↳nucleotide binding	254	87	46.21	1.88	+	5.45E-04
↳small molecule binding	397	113	72.23	1.56	+	2.62E-02
↳nucleoside phosphate binding	254	87	46.21	1.88	+	5.45E-04
oxidoreductase activity	436	120	79.32	1.51	+	4.27E-02
transferase activity	1572	358	286.01	1.25	+	4.61E-02
Unclassified	10792	1664	1963.47	.85	-	0.00E00
RNA polymerase II cis-regulatory region sequence-specific DNA binding	1054	37	191.76	.19	-	1.98E-36
↳cis-regulatory region sequence-specific DNA binding	1057	38	192.31	.20	-	5.30E-36
↳transcription regulatory region sequence-specific DNA binding	1377	60	250.53	.24	-	3.02E-41
↳regulatory region nucleic acid binding	1377	60	250.53	.24	-	3.02E-41
↳sequence-specific double-stranded DNA binding	1403	71	255.26	.28	-	1.11E-36
↳sequence-specific DNA binding	1436	82	261.26	.31	-	7.58E-33
↳DNA binding	1648	132	299.83	.44	-	4.81E-23
↳double-stranded DNA binding	1451	84	263.99	.32	-	1.11E-32
↳RNA polymerase II transcription regulatory region sequence-specific DNA binding	1337	48	243.25	.20	-	2.87E-46
DNA-binding transcription repressor activity, RNA polymerase II-specific	121	2	22.01	.09	-	2.59E-04
↳DNA-binding transcription repressor activity	121	2	22.01	.09	-	2.59E-04
↳DNA-binding transcription factor activity	1313	38	238.88	.16	-	5.85E-52
↳transcription regulator activity	1559	109	283.64	.38	-	2.04E-27
↳molecular function regulator	2358	230	429.01	.54	-	4.22E-22
↳DNA-binding transcription factor activity, RNA polymerase II-specific	1226	34	223.06	.15	-	1.95E-49
G protein-coupled receptor activity	275	4	50.03	.08	-	1.07E-12
↳transmembrane signaling receptor activity	669	18	121.72	.15	-	2.67E-26
↳signaling receptor activity	783	20	142.46	.14	-	6.56E-32
↳molecular transducer activity	783	20	142.46	.14	-	6.56E-32
voltage-gated cation channel activity	99	1	18.01	.06	-	1.06E-03

↳cation channel activity	254	7	46.21	.15	-	9.63E-09
↳ion channel activity	320	14	58.22	.24	-	5.55E-08
↳inorganic molecular entity transmembrane transporter activity	558	49	101.52	.48	-	3.61E-05
↳transmembrane transporter activity	717	67	130.45	.51	-	5.51E-06
↳transporter activity	811	93	147.55	.63	-	3.18E-03
↳ion transmembrane transporter activity	587	55	106.80	.51	-	1.49E-04
↳channel activity	343	17	62.40	.27	-	1.09E-07
↳passive transmembrane transporter activity	343	17	62.40	.27	-	1.09E-07
↳inorganic cation transmembrane transporter activity	396	28	72.05	.39	-	2.10E-05
↳cation transmembrane transporter activity	428	31	77.87	.40	-	1.07E-05
↳voltage-gated ion channel activity	110	4	20.01	.20	-	3.81E-02
↳voltage-gated channel activity	110	4	20.01	.20	-	3.81E-02
↳gated channel activity	204	7	37.12	.19	-	1.22E-05
potassium channel activity	108	1	19.65	.05	-	1.93E-04
↳potassium ion transmembrane transporter activity	133	4	24.20	.17	-	2.00E-03
↳monovalent inorganic cation transmembrane transporter activity	261	19	47.49	.40	-	9.17E-03
↳metal ion transmembrane transporter activity	313	19	56.95	.33	-	3.02E-05
neurotransmitter receptor activity	114	1	20.74	.05	-	8.48E-05
neurotransmitter binding	121	1	22.01	.05	-	2.40E-05
cytokine activity	129	1	23.47	.04	-	6.85E-06
↳receptor ligand activity	245	6	44.57	.13	-	6.49E-09
↳signaling receptor binding	680	49	123.72	.40	-	2.97E-10
↳signaling receptor activator activity	247	6	44.94	.13	-	4.36E-09
↳receptor regulator activity	276	6	50.21	.12	-	5.93E-11

About | Release Information | Contact Us | System Requirements | Privacy Policy | Disclaimer
© Copyright 2022 Paul Thomas All Rights Reserved.
