# Supplementary material for: Protective Signature of IFNγ-Stimulated Microglia Relies on miR-124-3p Regulation From the Secretome Released by Mutant APP Swedish Neuronal Cells
Source: Front Pharmacol. 2022 May 10;13:833066. doi: 10.3389/fphar.2022.833066 (PMC9127204; doi:10.3389/fphar.2022.833066)
Supplement: Supplementary file 3 [file DataSheet3.docx]

Supplementary Material

***Supplementary Tables:***

**Table S1**. List of primers used for mRNA/miRNA analysis by RT-qPCR.

**Table S2**. Proteomic analysis: List of proteins differentially expressed by the IFNγ-stimulated microglia after coculture with the SWE cells modulated with miR-124 inhibitor or mimic, relatively to those in the microglia incubated with the SWE mock controls.

**Table S3**. Gene Ontology (GO) analysis of proteomic data, using the PANTHER platform: Summary and results.

***Supplementary Figures:***

**Figure S1.** Protein distribution and correlation between replicates after normalization of proteomic data.

**Figure S2.** Relative quantification of the proteomic dataset of IFNγ-stimulated CHME3 microglia upon coculture with the SWE cells treated with either the inhibitor or the mimic of miR-124 *vs*. the mock control

**Figure S3.** Integrative analysis of the full proteomic data in miRNA database (miRnet.ca).

**Figure S4.** Venn diagram comparing our microglia proteomic data with other either similar or more advanced experimental models.

**Figure S5.** Exosomes released from Dicer1-silenced IFNγ-MG show low miR-124 content after microglia processing of exosomes from miR-124 modulated SWE cells.

**Table S1.** - List of primers used for mRNA/miRNA analysis by RT-qPCR.

| **miRNAs** | | **Target sequence** |
| --- | --- | --- |
| hsa-miR-124-3p | | UAAGGCACGCGGUGAAUGCC |
| hsa-miR-125b-5p | | UCCCUGAGACCCUAACUUGUGA |
| hsa-miR-21-5p | | UAGCUUAUCAGACUGAUGUUGA |
| hsa-miR-146a-5p | | UGAGAACUGAAUUCCAUGGGUU |
| hsa-miR-155-5p | | UUAAUGCUAAUCGUGAUAGGGGU |
| UniSp6 (spike-in) | | (Reference sequence) |
| U6 | | (Reference sequence) |
| **mRNAs** | | **Primer sequence (5′ to 3′)** |
| *RAGE* | **FWD:** TTCACGAAGTTCCAAACAGGT | |
|  | **REV:** GTTCTAGGACGACTGGGGTG | |
| *HMGB1* | **FWD:** CATCTCAGGGCCAAACCGATA | |
|  | **REV:** AGCAGACATGGTCTTCCACC | |
| *TNF-α* | **FWD:** AACCTCCTCTCTGCCATC | |
|  | **REV:** ATGTTCGTCCTCCTCACA | |
| *S100b* | **FWD:** TGTAGACCCTAACCCGGAGG | |
|  | **REV:** TGCATGGATGAGGAACGCAT | |
| *iNOS* | **FWD:** TCCGAGGCAAACAGCACATTCA | |
|  | **REV:** GGGTTGGGGGTGTGGTGATGT | |
| *MHC-II* | **FWD:** AGGGATTGCGCAAAAGCA | |
|  | **REV:** TCACCTCCATGTGCCTTACAGA | |
| *IL-1β* | **FWD:** GGGCCTCAAGGAAAAGAATC | |
|  | **REV:** TTCTGCTTGAGAGGTGCTGA | |
| *IL-6* | **FWD:** ATGAACTCCTTCTCCACAAGC | |
|  | **REV:** GTTTTCTGCCAGTGCCTCTTTG | |
| *IL-10* | **FWD:** CCTGGAGGAGGTGATGCCCCA | |
|  | **REV:** CCTGCTCCACGGCCTTGCTC | |
| *ARG1* | **FWD:** TGGAAACTTGCATGGACA | |
|  | **REV:** AAGTCCGAAACAAGCCAA | |
| *TGF-β* | **FWD:** TGCGCTTGAGATCTTCAAA | |
|  | **REV:** GGGCTAGTCGCACAGAACT | |
| *C/EBPα* | **FWD:** CAAAGCCAAGAAGTCGGTGGACAA | |
|  | **REV:** TCATTGTGACTGGTCAACTCCAGC | |
| *GAPDH* | **FWD:** CGCTCTCTGCTCCTCCTGTT | |
|  | **REV:** CCATGGTGTCTGAGCGATGT | |

hsa, *Homo sapiens*; miR, microRNA; mRNA, messenger RNA; RT-qPCR, real-time quantitative polymerase chain reaction; FWD, forward; REV, reverse; *RAGE*, receptor for advanced glycation end-products coding gene; *HMGB1*, high mobility group box protein 1 coding gene; *TNF*-α, tumor necrosis factor-α coding gene; *S100b*, S100 calcium-binding protein B coding gene; *iNOS*, nitric oxide synthase coding gene; *MHC-II*, major histocompatibility complex class II coding gene; *IL-1β*, interleukin 1 beta coding gene; *IL-6*, interleukin 6 coding gene; *IL-10*, interleukin 10 coding gene; *ARG1*, arginase-1 coding gene; *TGF-β*, transforming growth factor beta coding gene; *C/EBP*α, CCAAT enhancer binding protein alpha coding gene; *GAPDH*, glyceraldehyde-3-phosphate dehydrogenase coding gene.

**Table S2**. Proteomic analysis: List of proteins differentially expressed by the IFNγ-stimulated microglia after coculture with the SWE cells modulated with miR-124 inhibitor or mimic, relatively to those in the microglia incubated with the SWE mock controls.

| **Gene symbol** | **Protein ID** | **Description** | **Hit annotation** | **miR-124 inhibitor** | | **miR-124 mimic** | |
| --- | --- | --- | --- | --- | --- | --- | --- |
|  |  |  |  | **Log2 (FC)** | ***p* value** | **Log2 (FC)** | ***p* value** |
| SERPINB1 | P30740 | Leukocyte elastase inhibitor | hit | 0.16 | 3.92E-01 | -1.19 | 1.13E-09 |
| MAP2K6 | P52564 | Dual specificity mitogen-activated protein kinase kinase 6 | hit | 0.13 | 5.81E-01 | -0.97 | 6.63E-06 |
| PRKAR2B | P31323 | cAMP-dependent protein kinase type II-beta regulatory subunit | hit | 0.20 | 2.12E-01 | -0.93 | 4.33E-10 |
| DDAH1 | O94760 | N(G),N(G)-dimethylarginine dimethylaminohydrolase 1 | candidate | 0.15 | 2.70E-01 | -0.76 | 1.74E-09 |
| LYN | P07948\|P07948-2 | Tyrosine-protein kinase Lyn | candidate | 0.08 | 6.50E-01 | -0.51 | 1.39E-03 |
| LPCAT2 | Q7L5N7 | Lysophosphatidylcholine acyltransferase 2 | candidate | 0.34 | 2.02E-02 | -0.30 | 2.22E-02 |
| UBAP2L | F8W726\|Q14157\|Q14157-1\|Q14157-3\|Q14157-4\|Q14157-5 | Ubiquitin-associated protein 2-like | candidate | -0.50 | 5.18E-03 | 0.12 | 4.69E-01 |
| G3BP1 | Q13283 | Ras GTPase-activating protein-binding protein 1 | candidate | -0.57 | 5.18E-03 | 0.13 | 4.84E-01 |
| ADAMTS1 | Q9UHI8 | A disintegrin and metalloproteinase with thrombospondin motifs 1 | candidate | -0.52 | 2.30E-04 | 0.19 | 1.44E-01 |
| TGFB1 | P01137 | Transforming growth factor beta 1 | candidate | -0.52 | 3.93E-03 | 0.19 | 2.51E-01 |
| ICAM1 | P05362 | Intercellular adhesion molecule 1 | candidate | -0.76 | 6.77E-03 | 0.19 | 4.58E-01 |
| PAWR | Q96IZ0 | PRKC apoptosis WT1 regulator protein | hit | -0.64 | 2.43E-07 | 0.51 | 3.00E-04 |
| EFEMP1 | Q12805\|Q12805-2\|Q12805-3\|Q12805-4 | EGF-containing fibulin-like extracellular matrix protein 1 | hit | -0.74 | 2.75E-03 | 0.58 | 9.91E-03 |
| YBX3 | P16989\|P16989-2 | Y-box-binding protein 3 | candidate | -0.20 | 4.18E-01 | 0.71 | 1.81E-03 |
| GREM1 | O60565 | Gremlin-1 | candidate | -0.21 | 3.08E-01 | 0.79 | 1.65E-05 |
| FERMT3 | Q86UX7\|Q86UX7-2 | Fermitin family homolog 3 | candidate | 0.18 | 2.34E-01 | 0.95 | 2.20E-12 |
| AEBP1 | Q8IUX7\|Q8IUX7-2 | Adipocyte enhancer-binding protein 1 | hit | -0.20 | 4.33E-01 | 1.64 | 4.70E-13 |
| GAP43 | P17677\|P17677-2 | Neuromodulin | hit | 0.00 | 9.90E-01 | -2.29 | 9.19E-11 |
| VGF | O15240 | Neurosecretory protein VGF | candidate | -0.23 | 5.25E-01 | -1.61 | 1.02E-06 |
| NEFL | P07196 | Neurofilament light polypeptide | hit | -0.05 | 7.83E-01 | -1.46 | 2.22E-16 |
| NEFM | E7EMV2\|E7ESP9\|P07197 | Neurofilament medium polypeptide | hit | 0.08 | 6.57E-01 | -1.31 | 1.33E-15 |
| NCAM2 | H9KV31\|O15394 | Neural cell adhesion molecule 2 | hit | 0.09 | 5.44E-01 | -1.10 | 1.11E-15 |
| SV2A | Q7L0J3\|Q7L0J3-2 | Synaptic vesicle glycoprotein 2A | candidate | 0.09 | 4.90E-01 | -0.72 | 7.40E-09 |
| ENO2 | F5H0C8\|P09104\|P09104-2 | Gamma-enolase | candidate | 0.04 | 8.08E-01 | -0.59 | 9.03E-05 |
| DCX | A0A140LJL1\|H3BLV5\|O43602\|O43602-2 | Neuronal migration protein doublecortin | candidate | 0.15 | 3.68E-01 | -0.77 | 4.69E-07 |
| RUFY3 | Q7L099\|Q7L099-2\|Q7L099-3\|Q7L099-4 | Protein RUFY3 | hit | 0.21 | 1.27E-01 | -1.09 | 2.22E-16 |
| MAB21L1 | Q13394 | Protein mab-21-like 1 | hit | 0.40 | 4.90E-02 | -0.68 | 2.32E-04 |
| MAP1B | P46821 | Microtubule-associated protein 1B | candidate | 0.42 | 4.70E-02 | -0.30 | 1.15E-01 |
| SYNPO2 | H0Y9Y3\|Q9UMS6\|Q9UMS6-2\|Q9UMS6-3\|Q9UMS6-4 | Synaptopodin-2 (Fragment) | hit | 0.52 | 2.49E-04 | -1.15 | 2.22E-16 |
| MAP2 | P11137-4 | Isoform 4 of microtubule-associated protein 2 | hit | 0.41 | 2.41E-01 | -1.34 | 2.50E-05 |
| DPYSL5 | Q9BPU6 | Dihydropyrimidinase-related protein 5 | hit | 0.39 | 1.86E-02 | -2.03 | 2.22E-16 |
| DPYSL3 | Q14195-2 | Isoform LCRMP-4 of Dihydropyrimidinase-related protein 3 | hit | 0.32 | 9.33E-02 | -1.64 | 2.22E-16 |
| CRMP1 | E9PD68\|Q14194\|Q14194-2 | Dihydropyrimidinase-related protein 1 | hit | 0.29 | 5.44E-02 | -1.58 | 2.22E-16 |
| DPYSL2 | Q16555 | Dihydropyrimidinase-related protein 2 | candidate | 0.23 | 1.57E-01 | -0.63 | 1.62E-05 |
| STXBP1 | P61764\|P61764-2 | Syntaxin-binding protein 1 | candidate | 0.19 | 1.71E-01 | -0.46 | 3.46E-04 |
| CHURC1 | A0A0C4DGJ7\|H0YIM9\|Q8WUH1\|Q8WUH1-2 | Protein Churchill | candidate | -0.28 | 9.90E-02 | 0.34 | 2.78E-02 |
| AKR1C2\|AKR1C3\|AKR1C1\|AKR1C4 | A0A0A0MSD5\|A0A0A0MSS8\|A0A0A0MT30\|B4DK69\|H0Y804\|P17516\|P42330\|P52895\|Q04828\|S4R3Z2 | Aldo-keto reductase family 1 member C1-C4 | candidate | -0.04 | 7.83E-01 | 0.67 | 4.60E-07 |
| SNAP25 | P60880 | Synaptosomal-associated protein 25 | candidate | -0.04 | 2.51E-01 | 0.67 | 5.34E-03 |
| ERI1 | Q8IV48 | 3'-5' exoribonuclease 1 | candidate | 0.18 | 2.19E-01 | -0.43 | 1.54E-03 |
| SRSF4 | Q08170 | Serine/arginine-rich splicing factor 4 | candidate | 0.21 | 3.30E-01 | -0.53 | 7.65E-03 |
| TROVE2 | P10155 | 60 kDa SS-A/Ro ribonucleoprotein | candidate | 0.09 | 5.71E-01 | -0.58 | 3.24E-05 |
| TAGLN3 | Q9UI15 | Transgelin-3 | hit | 0.15 | 2.76E-01 | -1.17 | 2.22E-16 |
| ELAVL4 | A0A0R4J2E6\|B1APY8\|B1APY9\|P26378\|P26378-2\|P26378-3\|P26378-4\|P26378-5 | ELAV-like protein | hit | 0.15 | 3.70E-01 | -1.76 | 2.22E-16 |
| ZKSCAN1 | B3KRF7\|E9PC66\|P17029 | Zinc finger protein with KRAB and SCAN domains 1 | hit | 0.25 | 9.06E-02 | -0.96 | 7.26E-13 |
| SRSF12 | Q8WXF0 | Serine/arginine-rich splicing factor 12 | hit | 0.84 | 3.52E-07 | -0.93 | 5.18E-10 |
| RSRC2 | Q7L4I2\|Q7L4I2-2 | Arginine/serine-rich coiled-coil protein 2 | candidate | 0.35 | 1.24E-01 | -0.39 | 6.01E-02 |
| WBP4 | O75554\|O75554-2 | WW domain-binding protein 4 | candidate | -0.22 | 1.84E-01 | 0.38 | 1.13E-02 |
| EEF1D | E9PK01\|P29692 | Elongation factor 1-delta (Fragment) | candidate | -0.20 | 1.94E-01 | 0.43 | 2.69E-03 |
| NUFIP2 | Q7Z417 | Nuclear fragile X mental retardation-interacting protein 2 | candidate | -0.16 | 3.12E-01 | 0.44 | 2.44E-03 |
| FUS | H3BPE7\|P35637\|P35637-2 | RNA-binding protein FUS | candidate | -0.57 | 1.09E-02 | 0.15 | 4.42E-01 |
| RBMX | P38159\|P38159-2 | RNA-binding motif protein, X chromosome | candidate | -0.56 | 1.24E-02 | 0.18 | 3.64E-01 |
| HSDL1 | Q3SXM5 | Inactive hydroxysteroid dehydrogenase-like protein 1 | candidate | 0.49 | 1.27E-02 | -0.23 | 1.88E-01 |
| GDAP1 | Q8TB36 | Ganglioside-induced differentiation-associated protein 1 | candidate | 0.41 | 2.04E-01 | -0.73 | 1.24E-02 |
| CPT1A | P50416\|P50416-2 | Carnitine O-palmitoyltransferase 1 | candidate | 0.35 | 2.52E-02 | -0.53 | 1.72E-04 |
| FASTKD2 | Q9NYY8 | FAST kinase domain-containing protein 2 | candidate | 0.40 | 5.14E-03 | -0.33 | 1.10E-02 |
| KIF5C | O60282 | Kinesin heavy chain isoform 5C | candidate | 0.31 | 4.83E-02 | -0.33 | 1.97E-02 |
| SLC25A13 | Q9UJS0\|Q9UJS0-2 | Calcium-binding mitochondrial carrier protein Aralar2 | candidate | 0.26 | 9.97E-02 | -0.40 | 4.40E-03 |
| ALDH2 | P05091\|P05091-2 | Aldehyde dehydrogenase, mitochondrial | candidate | 0.21 | 1.74E-01 | -0.78 | 3.74E-08 |
| SFXN3 | A0A0A0MS41\|Q9BWM7 | Sideroflexin | candidate | 0.19 | 1.61E-01 | -0.53 | 2.15E-05 |
| MFN1 | A0A0C4DFN1\|Q8IWA4\|Q8IWA4-3 | Mitofusin-1 | candidate | 0.10 | 5.38E-01 | -0.56 | 7.88E-05 |
| BNIP3 | Q12983 | BCL2/adenovirus E1B 19 kDa protein-interacting protein 3 | candidate | -0.27 | 1.21E-01 | 0.35 | 2.70E-02 |
| HEBP2 | Q9Y5Z4 | Heme-binding protein 2 | candidate | -0.72 | 4.56E-03 | 0.28 | 2.27E-01 |
| INA | A0A087WYG8\|Q16352 | Alpha-internexin | candidate | 0.08 | 6.05E-01 | -0.90 | 1.94E-11 |
| PDXP | Q96GD0 | Pyridoxal phosphate phosphatase | candidate | 0.05 | 7.34E-01 | -0.54 | 8.48E-05 |
| TES | Q9UGI8\|Q9UGI8-2 | Testin | candidate | 0.25 | 1.83E-01 | -0.63 | 1.83E-04 |
| FSCN1 | Q16658 | Fascin | candidate | 0.25 | 8.15E-02 | -0.65 | 1.07E-06 |
| MICAL1 | Q8TDZ2\|Q8TDZ2-2\|Q8TDZ2-4 | Protein-methionine sulfoxide oxidase MICAL1 | candidate | 0.43 | 3.50E-02 | -0.52 | 4.84E-03 |
| AFAP1 | Q8N556\|Q8N556-2 | Actin filament-associated protein 1 | candidate | 0.35 | 1.89E-02 | -0.31 | 2.43E-02 |
| DNM1 | A0A0D9SFB1\|A0A0U1RQP1\|Q05193\|Q05193-2\|Q05193-3\|Q05193-5 | Dynamin-1 | candidate | 0.39 | 5.69E-03 | -0.25 | 5.07E-02 |
| COL6A1 | A0A087X0S5\|P12109 | Collagen alpha-1(VI) chain | candidate | -0.22 | 1.58E-01 | 0.51 | 3.39E-04 |
| CCDC80 | Q76M96\|Q76M96-2 | Coiled-coil domain-containing protein 80 | candidate | -0.15 | 4.05E-01 | 0.74 | 5.42E-06 |
| LAMP1 | P11279 | Lysosome-associated membrane glycoprotein 1 | candidate | -0.34 | 2.93E-02 | 0.25 | 7.44E-02 |
| PTRF | Q6NZI2 | Polymerase I and transcript release factor | candidate | -0.44 | 1.24E-02 | 0.41 | 1.04E-02 |
| DNAJB11 | Q9UBS4 | DnaJ homolog subfamily B member 11 | candidate | -0.51 | 4.75E-02 | 0.38 | 1.04E-01 |
| CLEC3B | P05452 | Tetranectin | hit | -0.67 | 6.43E-04 | 0.46 | 9.69E-03 |
| ATP7A | Q04656\|Q04656-2\|Q04656-3\|Q04656-5 | Copper-transporting ATPase 1 | hit | 0.52 | 1.46E-02 | -0.56 | 3.77E-03 |
| SOGA3 | E9PJP2\|Q5TF21 | Protein SOGA3 | hit | 0.27 | 1.91E-01 | -1.48 | 4.22E-15 |
| RAB3D | O95716 | Ras-related protein Rab-3D | hit | 0.18 | 2.08E-01 | -1.05 | 2.44E-15 |
| GDI1 | P31150 | Rab GDP dissociation inhibitor alpha | candidate | 0.20 | 2.05E-01 | -0.40 | 5.86E-03 |
| ALDOC | P09972 | Fructose-bisphosphate aldolase C | candidate | 0.17 | 3.62E-01 | -0.65 | 8.94E-05 |
| ATL1 | Q8WXF7\|Q8WXF7-2 | Atlastin-1 | candidate | 0.16 | 2.66E-01 | -0.68 | 2.73E-07 |
| TBC1D5 | Q92609\|Q92609-2 | TBC1 domain family member 5 | candidate | 0.12 | 4.26E-01 | -0.47 | 9.95E-04 |
| ASAP2 | O43150\|O43150-2 | Arf-GAP with SH3 domain, ANK repeat and PH domain-containing protein 2 | candidate | 0.00 | 9.93E-01 | -0.64 | 1.63E-05 |
| PRAF2 | A6NP52\|O60831 | PRA1 family protein 2 | candidate | -0.18 | 2.07E-01 | -0.89 | 4.91E-12 |
| CHGB | A0A0A0MT66\|P05060 | Secretogranin-1 (Fragment) | hit | 0.06 | 8.06E-01 | -1.49 | 3.23E-10 |
| SCG2 | P13521 | Secretogranin-2 | candidate | -0.46 | 1.51E-01 | -1.57 | 7.17E-08 |
| CTGF | P29279 | Connective tissue growth factor | hit | -0.32 | 1.01E-01 | 0.74 | 2.28E-05 |
| COL5A1 | A0A087WXW9\|P20908 | Collagen alpha 1(V) chain | candidate | -0.44 | 4.37E-02 | 0.54 | 6.91E-03 |
| PCNP | Q8WW12 | PEST proteolytic signal-containing nuclear protein | candidate | -0.62 | 3.60E-02 | 0.48 | 7.42E-02 |
| COMP | G3XAP6\|P49747\|P49747-2 | Cartilage oligomeric matrix protein | candidate | -0.42 | 8.43E-02 | 0.44 | 4.60E-02 |
| UBE2C | A0A087WVK1\|O00762\|O00762-3\|O00762-4 | Ubiquitin-conjugating enzyme E2 C | candidate | -0.33 | 4.72E-02 | 0.30 | 4.73E-02 |
| SRFBP1 | Q8NEF9 | Serum response factor-binding protein 1 | candidate | -0.42 | 1.97E-02 | 0.22 | 1.75E-01 |
| APOL3 | O95236\|O95236-2 | Apolipoprotein L3 | candidate | -0.49 | 1.63E-03 | 0.13 | 3.51E-01 |
| RSU1 | Q15404 | Ras suppressor protein 1 | candidate | -0.66 | 5.82E-03 | 0.15 | 4.96E-01 |
| REXO2 | F5GYG5\|H0YG54\|H0YGR4\|Q9Y3B8\|Q9Y3B8-2\|Q9Y3B8-3 | Oligoribonuclease, mitochondrial | candidate | -0.61 | 4.32E-05 | 0.06 | 6.43E-01 |
| C9orf64 | Q5T6V5 | UPF0553 protein C9orf64 | candidate | 0.12 | 4.17E-01 | -0.50 | 1.20E-04 |
| IMPA1 | E5RG13\|E5RIP7\|H0YBL1\|P29218\|P29218-3 | Inositol monophosphatase 1 (Fragment) | candidate | 0.13 | 4.47E-01 | -0.52 | 1.25E-03 |
| H2AFY2 | Q9P0M6 | Core histone macro-H2A.2 | candidate | 0.16 | 3.81E-01 | -0.53 | 1.33E-03 |
| CDK6 | Q00534 | Cyclin-dependent kinase 6 | candidate | 0.17 | 2.74E-01 | -0.62 | 1.54E-05 |
| CDK5 | Q00535\|Q00535-2 | Cyclin-dependent-like kinase 5 | candidate | 0.17 | 2.77E-01 | -0.62 | 1.14E-05 |
| INPP4A | Q96PE3\|Q96PE3-2\|Q96PE3-3\|Q96PE3-4 | Type I inositol 3,4-bisphosphate 4-phosphatase | candidate | 0.01 | 9.54E-01 | -0.65 | 1.70E-04 |
| QPRT | Q15274 | Nicotinate-nucleotide pyrophosphorylase [carboxylating] | candidate | 0.05 | 6.96E-01 | -0.71 | 1.17E-08 |
| CTSB | P07858 | Cathepsin B | candidate | -0.15 | 3.77E-01 | -0.95 | 8.97E-10 |
| ADAM22 | F8WAD8\|Q9P0K1\|Q9P0K1-2\|Q9P0K1-3\|Q9P0K1-4\|Q9P0K1-5 | Disintegrin and metalloproteinase domain-containing protein 22 (Fragment) | hit | 0.00 | 9.85E-01 | -1.04 | 4.88E-15 |
| CRABP1 | P29762 | Cellular retinoic acid-binding protein 1 | hit | 0.10 | 5.47E-01 | -1.18 | 1.55E-15 |
| CACNA2D2 | C9JE82\|C9JVC9\|Q9NY47\|Q9NY47-2\|Q9NY47-3\|Q9NY47-4\|Q9NY47-5 | Voltage-dependent calcium channel subunit alpha-2/delta-2 | hit | 0.09 | 6.43E-01 | -1.13 | 5.63E-11 |
| CKB | P12277 | Creatine kinase B-type | candidate | 0.06 | 6.73E-01 | -0.90 | 1.29E-12 |
| RBP1 | A0A0A0MQT0\|P09455 | Retinol binding protein 1, cellular | hit | 0.22 | 1.10E-04 | -0.87 | 5.90E-01 |
| PHOX2A | O14813 | Paired mesoderm homeobox protein 2A | hit | 0.15 | 4.05E-01 | -1.68 | 2.22E-16 |
| GSK3B | P49841\|P49841-2 | Glycogen synthase kinase-3 beta | hit | -0.32 | 6.76E-02 | -1.47 | 2.22E-16 |
| C11ORF96 | A0A087WW59\|Q7Z7L8 | Uncharacterized protein C11orf96 | hit | 0.20 | 2.85E-01 | -1.62 | 2.22E-16 |
| RTL1 | A6NKG5 | Retrotransposon-like protein 1 | hit | 0.36 | 1.68E-02 | -1.75 | 2.22E-16 |
| NES | P48681 | Nestin | hit | 0.64 | 5.92E-03 | -1.41 | 1.78E-11 |
| DNMT3A | A0A0C4DG02\|F8WE91\|Q9Y6K1\|Q9Y6K1-2 | DNA (cytosine-5)-methyltransferase 3A | hit | 0.76 | 8.02E-06 | -0.27 | 7.93E-02 |

Gene Symbol, protein identification (ID) and description are according with the UniprotKB database and the computationally mapped potential isoforms are indicated in Protein ID column. Expression is presented as Log2 fold change (FC). Proteomic data presented in this table was uploaded to the PANTHER platform under the name “upload_1” for further functional classification based on Gene Ontology (GO) analysis against Homo sapiens database (Table S3).

**Table S3**. Gene Ontology (GO) analysis of proteomic data, using the PANTHER platform: Summary and results.

| **Analysis Type:** | PANTHER Overrepresentation Test (Released 2021.02.24) | |
| --- | --- | --- |
| **Annotation Version and Release Date:** | GO Ontology database DOI: 10.5281/zenodo.4495804 Released 2021-02-01 | |
| **Analyzed List:** | upload_1 (Homo sapiens) | |
| **Reference List:** | Homo sapiens (all genes in database) | |
| **Annotation Data Set:** | GO cellular component; GO biological process  GO molecular function | |
| **Test Type:** | Fisher's Exact | |
| **Correction:** | Bonferroni correction for multiple testing (*p* < 0.05) | |
| **Bonferroni counts:** | 1466 | |
|  |  | |
|  | **Reference list**  20595 out of 20595  0  0 | **Table_S2**  147 out of 154  8  5 |
| **Uniquely Mapped IDS:** |  |  |
| **Unmapped IDs:** |  |  |
| **Multiple mapping information:** |  |  |

|  | ***Homo sapiens*** (Reference) | **upload_1**  (Hierarchy) | | | | |
| --- | --- | --- | --- | --- | --- | --- |
| **GO cellular component** | **#** | **#** | **Expected** | **Fold enrichment** | **+/-** | ***p* value** |
| **Unclassified** | 1755 | 3 | 13.12 | 0.23 | - | 0.00E+00 |
| **Extracellular exosome** | 2098 | 38 | 15.69 | 2.42 | + | 4.18E-04 |
| extracellular vesicle | 2118 | 39 | 15.84 | 2.46 | + | 2.10E-04 |
| extracellular organelle | 2120 | 39 | 15.85 | 2.46 | + | 2.13E-04 |
| organelle | 13844 | 131 | 103.52 | 1.27 | + | 9.68E-04 |
| vesicle | 3919 | 55 | 29.3 | 1.88 | + | 2.10E-03 |
| extracellular space | 3391 | 47 | 25.36 | 1.85 | + | 2.39E-02 |
| **Synapse** | 1344 | 27 | 10.05 | 2.69 | + | 4.25E-03 |
| cell junction | 2097 | 43 | 15.68 | 2.74 | + | 1.05E-06 |
| **Secretory vesicle** | 1022 | 21 | 7.64 | 2.75 | + | 4.46E-02 |
| intracellular anatomical structure | 14773 | 138 | 110.47 | 1.25 | + | 1.67E-04 |
| cytoplasm | 11908 | 123 | 89.04 | 1.38 | + | 1.41E-05 |
| **Polymeric cytoskeletal fiber** | 770 | 19 | 5.76 | 3.3 | + | 9.42E-03 |
| supramolecular fiber | 998 | 22 | 7.46 | 2.95 | + | 9.69E-03 |
| supramolecular polymer | 1006 | 22 | 7.52 | 2.92 | + | 1.10E-02 |
| supramolecular complex | 1325 | 27 | 9.91 | 2.73 | + | 3.27E-03 |
| **Dendrite** | 640 | 20 | 4.79 | 4.18 | + | 1.47E-04 |
| dendritic tree | 642 | 20 | 4.8 | 4.17 | + | 1.54E-04 |
| neuron projection | 1376 | 37 | 10.29 | 3.6 | + | 1.43E-08 |
| plasma membrane  bounded cell projection | 2229 | 40 | 16.67 | 2.4 | + | 2.15E-04 |
| cell projection | 2325 | 41 | 17.39 | 2.36 | + | 2.12E-04 |
| Somatodendritic compartment | 870 | 28 | 6.51 | 4.3 | + | 1.49E-07 |
| **Collagen-containing extracellular matrix** | 427 | 17 | 3.19 | 5.32 | + | 5.28E-05 |
| extracellular matrix | 572 | 19 | 4.28 | 4.44 | + | 1.26E-04 |
| external encapsulating structure | 573 | 19 | 4.28 | 4.43 | + | 1.29E-04 |
| **Perikaryon** | 158 | 10 | 1.18 | 8.46 | + | 7.64E-04 |
| neuronal cell body | 512 | 22 | 3.83 | 5.75 | + | 1.08E-07 |
| cell body | 584 | 26 | 4.37 | 5.95 | + | 7.64E-10 |
| **Growth cone** | 184 | 13 | 1.38 | 9.45 | + | 4.07E-06 |
| distal axon | 300 | 16 | 2.24 | 7.13 | + | 2.70E-06 |
| axon | 650 | 26 | 4.86 | 5.35 | + | 7.79E-09 |
| site of polarized growth | 190 | 13 | 1.42 | 9.15 | + | 5.86E-06 |
|  |  |  |  |  |  |  |
| **GO biological process** | **#** | **#** | **Expected** | **Fold enrichment** | **+/-** | ***p* value** |
| **Unclassified** | 2815 | 5 | 15.86 | 0.32 | - | 0.00E+00 |
| **Response to axon injury** | 53 | 6 | .30 | 20.10 | + | 8.78E-03 |
| **Regulation of axon extension** | 96 | 7 | .54 | 12.95 | + | 1.68E-02 |
| regulation of extent of cell growth | 111 | 7 | .63 | 11.20 | + | 4.17E-02 |
| regulation of cellular component organization | 2274 | 31 | 12.81 | 2.42 | + | 3.24E-02 |
| cellular component organization | 5314 | 53 | 29.93 | 1.77 | + | 4.97E-02 |
| cellular component organization or biogenesis | 5517 | 55 | 31.07 | 1.77 | + | 2.31E-02 |
| **Axon development** | 387 | 13 | 2.18 | 5.96 | + | 3.57E-03 |
| neuron projection development | 654 | 18 | 3.68 | 4.89 | + | 3.35E-04 |
| neuron development | 817 | 19 | 4.60 | 4.13 | + | 1.77E-03 |
| cell development | 1649 | 26 | 9.29 | 2.80 | + | 1.35E-02 |
| cell differentiation | 3483 | 40 | 19.62 | 2.04 | + | 4.44E-02 |
| cellular developmental process | 3548 | 41 | 19.98 | 2.05 | + | 2.74E-02 |
| anatomical structure development | 5062 | 52 | 28.51 | 1.82 | + | 2.05E-02 |
| neuron differentiation | 1013 | 21 | 5.71 | 3.68 | + | 2.41E-03 |
| generation of neurons | 1248 | 26 | 7.03 | 3.70 | + | 6.15E-05 |
| neurogenesis | 1370 | 28 | 7.72 | 3.63 | + | 2.14E-05 |
| nervous system development | 2195 | 32 | 12.36 | 2.59 | + | 3.35E-03 |
| system development | 4222 | 47 | 23.78 | 1.98 | + | 1.18E-02 |
| multicellular organism development | 4564 | 49 | 25.71 | 1.91 | + | 1.42E-02 |
| **Neuron projection morphogenesis** | 463 | 15 | 2.61 | 5.75 | + | 6.73E-04 |
| plasma membrane bounded cell projection morphogenesis | 467 | 15 | 2.63 | 5.70 | + | 7.50E-04 |
| cell projection morphogenesis | 471 | 16 | 2.65 | 6.03 | + | 1.25E-04 |
| cell morphogenesis | 690 | 19 | 3.89 | 4.89 | + | 1.33E-04 |
| cell part morphogenesis | 490 | 16 | 2.76 | 5.80 | + | 2.14E-04 |
| cellular component morphogenesis | 581 | 16 | 3.27 | 4.89 | + | 2.06E-03 |
| **Cell morphogenesis involved in neuron differentiation** | 423 | 13 | 2.38 | 5.46 | + | 9.42E-03 |
| cell morphogenesis involved in differentiation | 535 | 14 | 3.01 | 4.65 | + | 2.23E-02 |
| **Regulation of neuron projection development** | 429 | 13 | 2.42 | 5.38 | + | 1.10E-02 |
| regulation of plasma membrane bounded cell projection organization | 620 | 17 | 3.49 | 4.87 | + | 8.80E-04 |
| regulation of cell projection organization | 636 | 17 | 3.58 | 4.75 | + | 1.25E-03 |
| **Positive regulation of cellular component organization** | 1028 | 20 | 5.79 | 3.45 | + | 1.27E-02 |
|  |  |  |  |  |  |  |
| **GO molecular function** | **#** | **#** | **Expected** | **Fold enrichment** | **+/-** | ***p* value** |
| **Unclassified** | 2328 | 4 | 13.11 | 0.31 | - | 0.00E+00 |
| **Cytoskeletal protein binding** | 998 | 20 | 5.62 | 3.56 | + | 2.61E-03 |
| protein binding | 14397 | 104 | 81.09 | 1.28 | + | 1.94E-03 |
| binding | 16551 | 112 | 93.22 | 1.20 | + | 1.35E-03 |

GO annotation for cellular component, biological process and molecular function of the proteomic data described in Table S2 (Table_S2) using the PANTHER Overrepresentation Test (Released 20210224), with Homo sapiens as reference. Summary of the analysis is showed in the top, and the full results (Hierarchically distributed) on the bottom, with respective fold enrichment and p value. Bonferroni correction for multiple testing (p < 0.05) was used as post-hoc for Fisher’s Exact test, as indicated in the platform. GO, gene ontology.

**
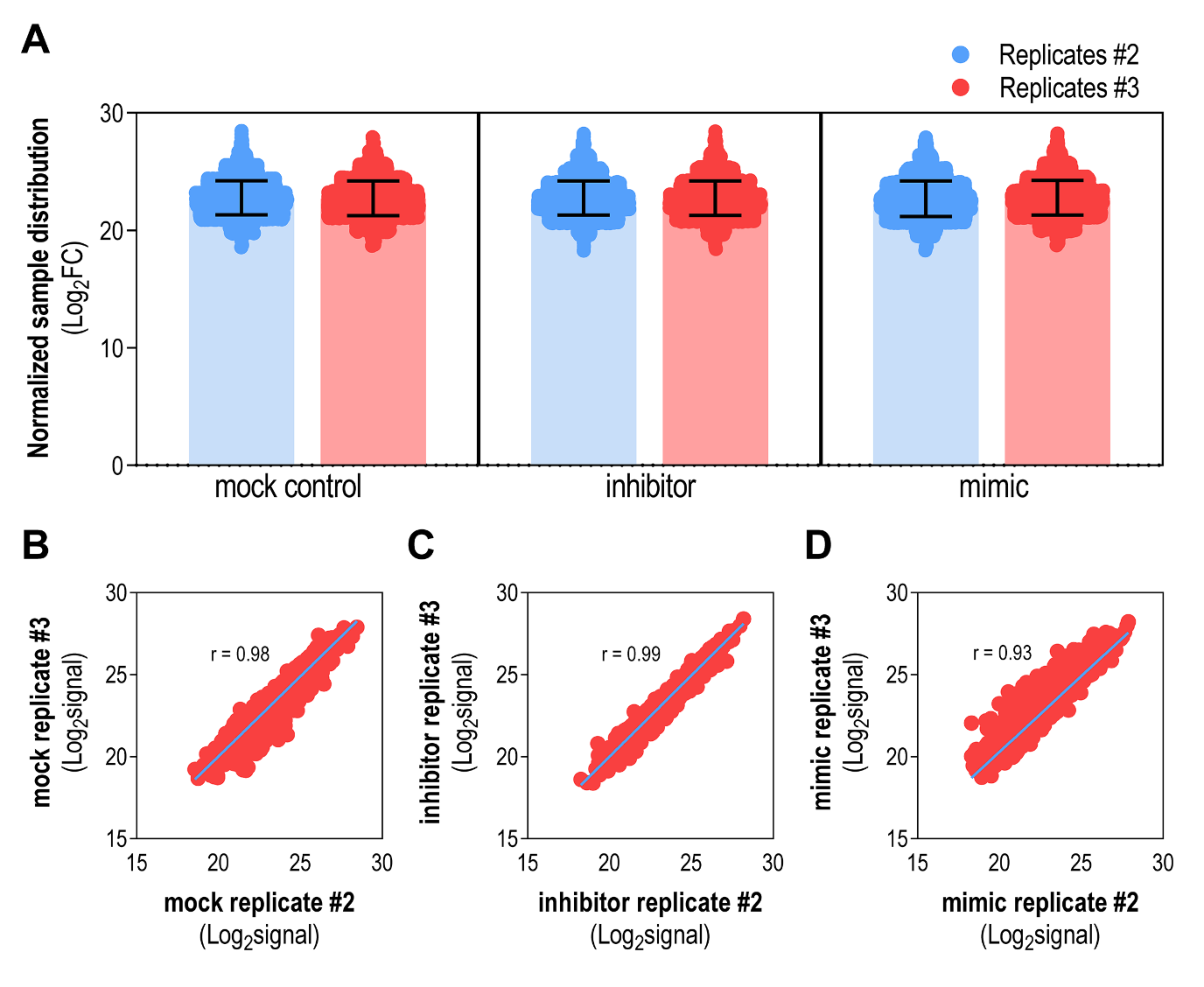
Figure S1.** Protein distribution and correlation between replicates after normalization of proteomic data. (**A**) Normalization overview of the protein distribution in each replicate of IFNγ-stimulated microglia cocultured with the miR-124 modulated SWE cells in 3 different conditions (mock, inhibitor and mimic). Correlation plots of the identified proteins between the 2 replicates from (**B**) mock control, (**C**) inhibitor and (**D**) mimic of miR-124. Correlation index in indicated as “r” in each plot.

**
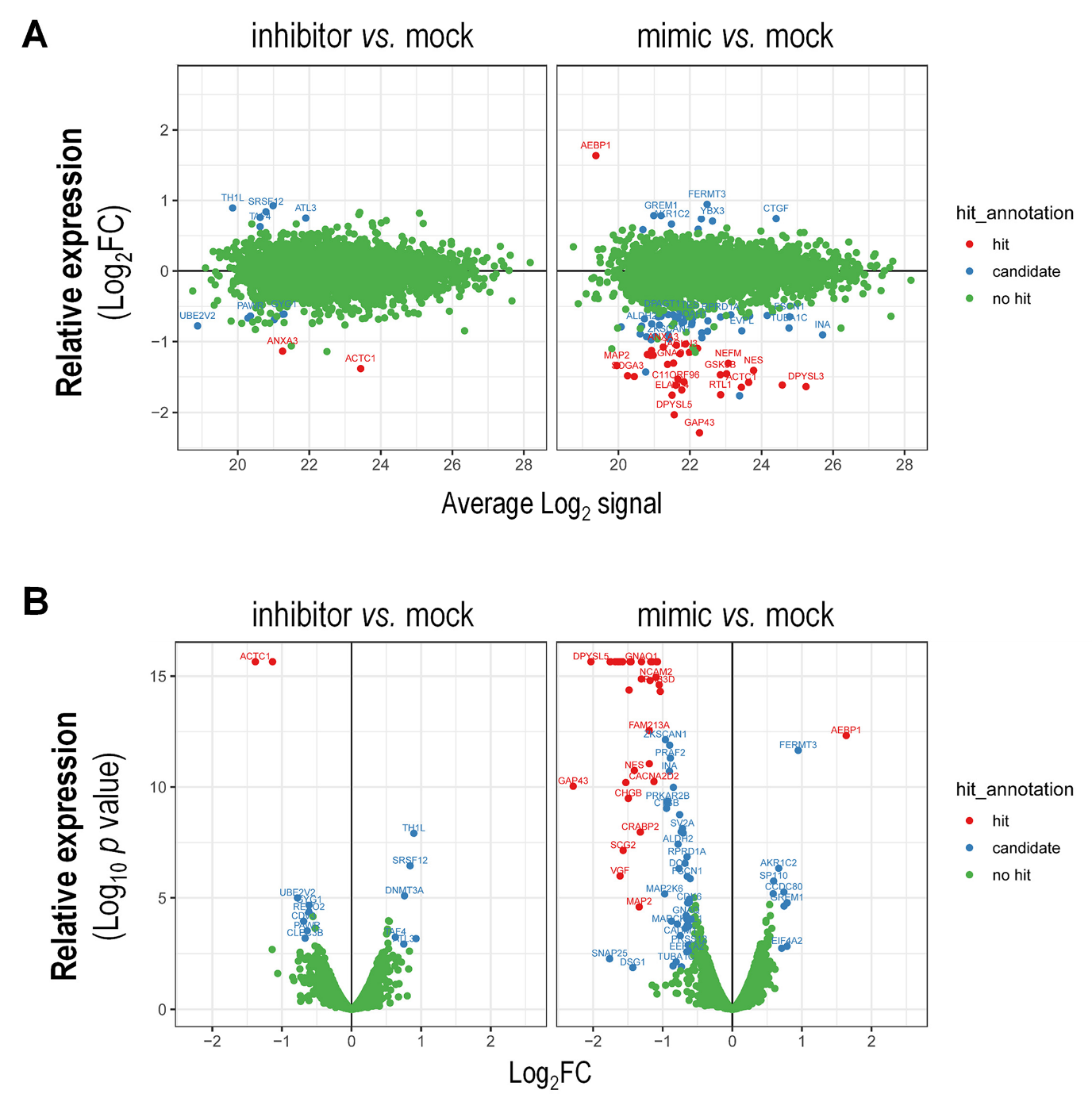
Figure S2.** Relative quantification of the proteomic dataset of IFNγ-stimulated CHME3 microglia upon coculture with the SWE cells treated with either the inhibitor or the mimic of miR-124 *vs*. the mock control. (**A**) MA plots and (**B**) volcano plots based on proteomic changes in CHME3 microglia in the 2 types of cocultures resultant from the miR-124 modulation. In the MA plots, the upper proteins represent the most upregulated, while the lower ones correspond to the downregulated ones. In volcano plots, the most upregulated proteins are towards the right and the most downregulated proteins are towards the left, while the most statistically significant proteins are towards the top. Hit proteins (red dots), candidate proteins (blue dots) and no-hit proteins (green dots) are displayed in both graphical representations.


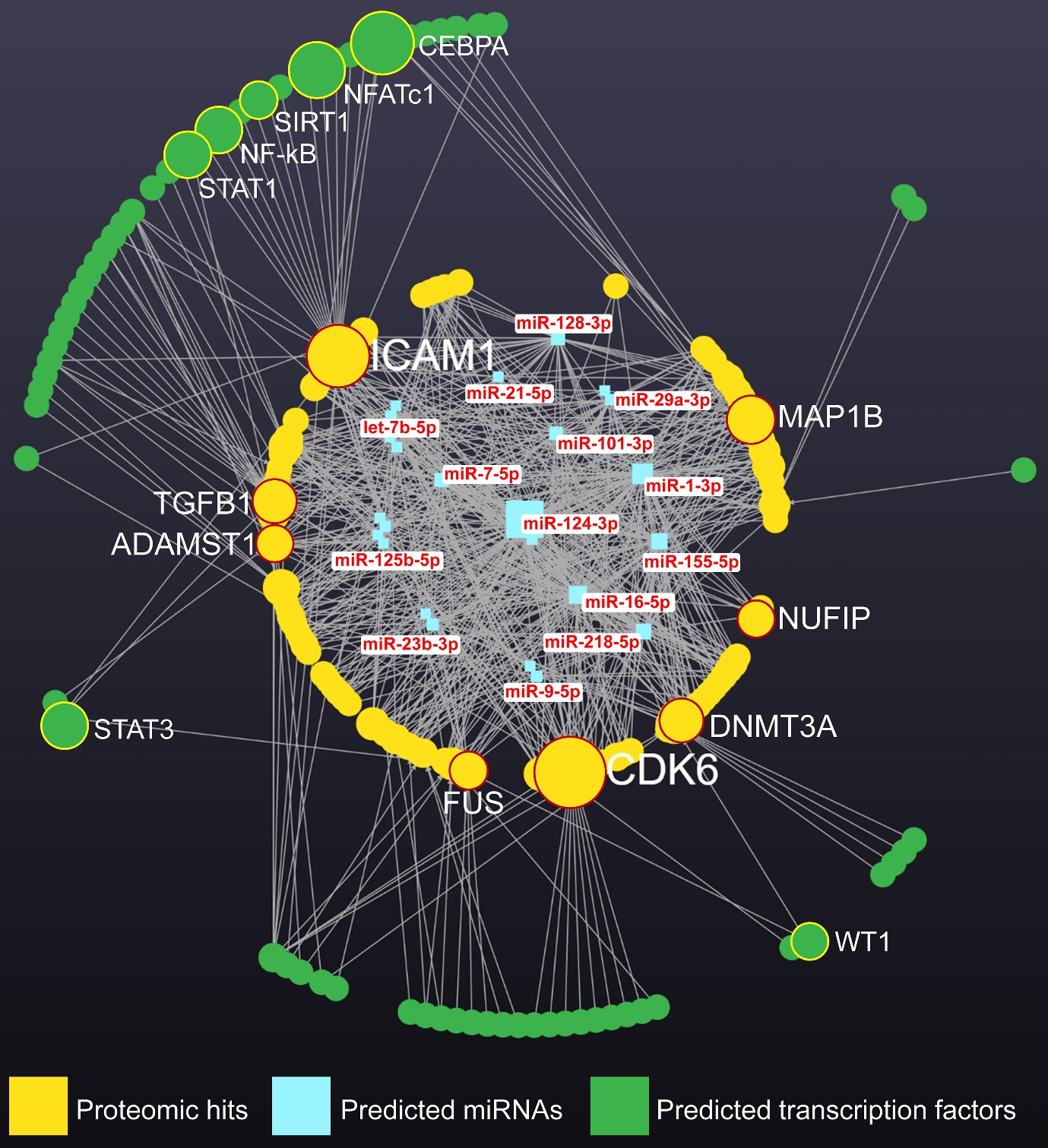


**Figure S3.** Integrative analysis of the full proteomic data in miRNA database (miRnet.ca). The full list of proteins differentially expressed in proteomic analysis (Table S2) was searched in miRnet.ca to identify the most potentially involved miRNAs and transcription factors. In the middle, 14 of the most discriminating miRNAs are shown in light blue squares, with miR-124-3p leading with a total betweenness value of 4297.024. Bigger squares indicate higher betweenness value. Closely grouped squares indicate alternative miRNA entries for the same miRNA (3-p, 5-p, a or b forms). The yellow circles indicate the proteomic data, with outlined circles (in red) representing those more susceptible of being regulated by these miRNAs. In green circles, it is shown the predicted transcription factors that may be involved. Some of the most involved ones are outlined (in yellow). In miRnet.ca platform, we used the KEGG database for pathway enrichment, based on the hypergeometric algorithm for both miRNAs and proteomic data.

**
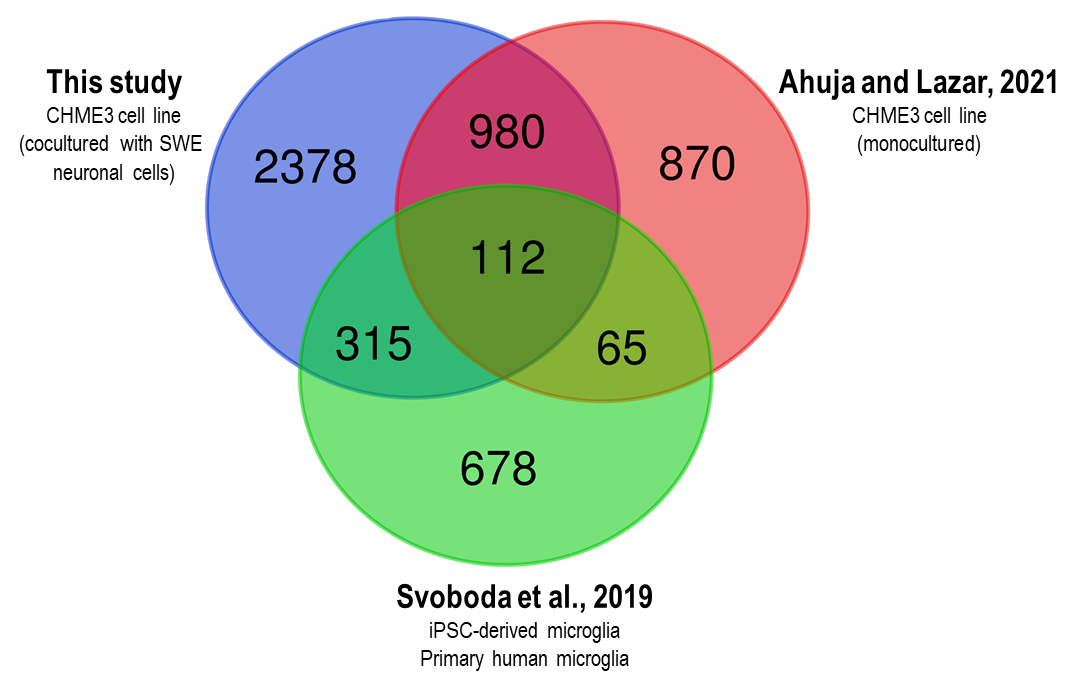
Figure S4.** Venn diagram comparing our microglia proteomic data with other either similar or more advanced experimental models. Proteomic full data from the present study (blue circle), proteomic data from a previous study using the same microglia cell line (aliased as HMC3) in a monoculture setup (red circle), and the consensus dataset from single-cell RNAseq data from induced pluripotent stem cell (iPSCS)-derived microglia and primary human microglia (green circle) are shown. Dataset comparison was performed from original research studies ([doi.org/10.3389/fimmu.2021.646043](https://doi.org/10.3389/fimmu.2021.646043) and [doi.org/10.1073/pnas.1913541116](https://doi.org/10.1073/pnas.1913541116)).


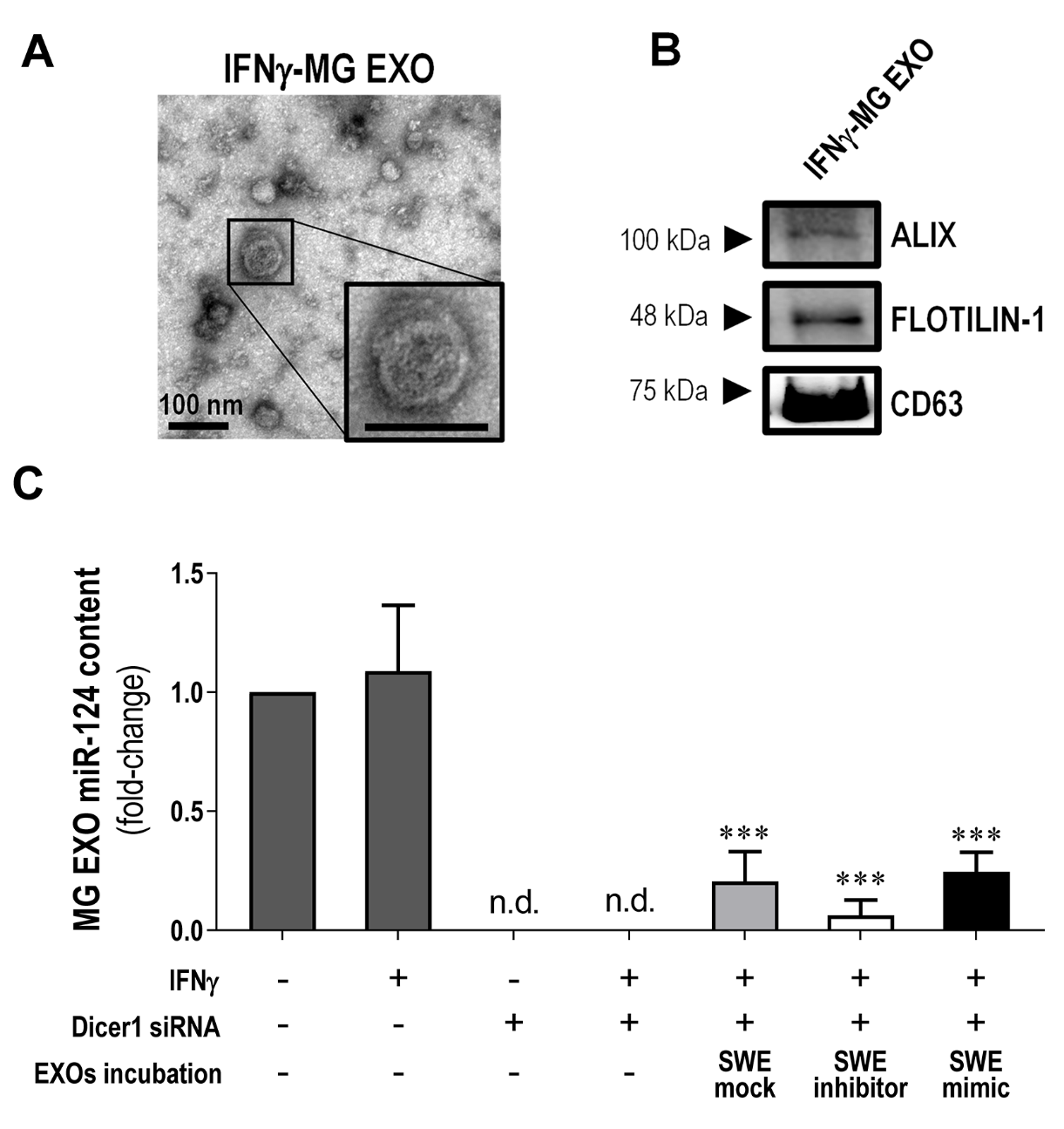
**Figure S5.** Exosomes released from Dicer1-silenced IFNγ-MG show low miR-124 content after microglia processing of exosomes from miR-124 modulated SWE cells. Exosomes were isolated by differential ultracentrifugation. (**A**) Representative transmission electron microscopy (TEM) images of exosomes for morphology and size. (**B**) Representative Western blots for the common exosomal protein markers, Alix, CD63 and Flotilin‐1 (FLOT‐1). (**C**) miR-124 levels in the exosomes derived from IFNγ-MG with or without incubation with exosomes from miR-124 modulated SWE cells (mock, inhibitor and mimic), during 24 h. IFNγ-MG cells were treated with Dicer1 siRNAs before incubation with SWE-derived EXOs. IFNγ-MG cells were washed, and fresh media was added for additional 24 h to allow *de novo* exosome sorting. Results are mean ± SD, from three independent experiments. *** *p* < 0.001 *vs.* naïve MG, one-way ANOVA with Bonferroni post-hoc test. SWE, human SH‐SY5Y expressing the APP695 Swedish mutant protein; IFNγ-MG, IFNγ-stimulated CHME3 microglia cells; EXO, exosomes; n.d., not detected.
